# Physical activity across midlife and health-related quality of life in Australian women: A target trial emulation using a longitudinal cohort

**DOI:** 10.1371/journal.pmed.1004384

**Published:** 2024-05-02

**Authors:** Binh Nguyen, Philip Clare, Gregore I. Mielke, Wendy J. Brown, Ding Ding

**Affiliations:** 1 Prevention Research Collaboration, Sydney School of Public Health, Faculty of Medicine and Health, The University of Sydney, Camperdown, Australia; 2 Charles Perkins Centre, The University of Sydney, Camperdown, Australia; 3 National Drug and Alcohol Research Centre, UNSW Sydney, Sydney, Australia; 4 School of Public Health, Faculty of Medicine, University of Queensland, Brisbane, Australia; 5 Faculty of Health Sciences and Medicine, Bond University, Gold Coast, Australia; 6 School of Human Movement and Nutrition Sciences, Faculty of Health and Behavioural Sciences, University of Queensland, Brisbane, Australia

## Abstract

**Background:**

There is little long-term causal evidence on the effect of physical activity on health-related quality of life. This study aimed to examine the associations between longitudinal patterns of physical activity over 15 years and health-related quality of life in both the physical and mental health domains, in a cohort of middle-aged Australian women.

**Methods and findings:**

We used data collected at 3-year intervals (1998 to 2019) from 11,336 participants in the Australian Longitudinal Study on Women’s Health (ALSWH) (1946 to 1951 birth cohort). Primary outcomes were the physical (PCS) and mental health component summary (MCS) scores (range from 0 to 100; higher scores indicate higher perceived physical/mental health) from the SF-36 in 2019 (when women aged 68 to 73 years). Using target trial emulation to imitate a randomized controlled trial (RCT), we tested 2 interventions: (1) meeting the World Health Organization (WHO) physical activity guidelines consistently throughout the 15-year “exposure period” (2001 to 2016; when women aged 50–55 to 65–70 years; physical activity assessed every 3 years); and (2) not meeting the guidelines at the beginning of the exposure period but starting to first meet the guidelines at age 55, 60, or 65; against the control of not meeting the guidelines throughout the exposure period. Analysis controlled for confounding using marginal structural models which were adjusted for sociodemographic and health variables and conditions. Consistent adherence to guidelines during the exposure period (PCS: 46.93 [99.5% confidence interval [CI]: 46.32, 47.54]) and first starting to meet the guidelines at age 55 (PCS: 46.96 [99.5% CI: 45.53, 48.40]) were associated with three-point higher PCS (mean score difference: 3.0 [99.5% CI: 1.8, 4.1] and 3.0 [99.5% CI:1.2, 4.8]) than consistent non-adherence (PCS: 43.90 [99.5% CI: 42.79, 45.01]). We found a similar pattern for most SF-36 subscales but no significant effects of the interventions on MCS. The main limitations of the study were that it may not account for all underlying health conditions and/or other unmeasured or insufficiently measured confounders, the use of self-reported physical activity and that findings may not be generalizable to all mid-age women.

**Conclusions:**

Results from the emulated RCT suggest women should be active throughout mid-age, ideally increasing activity levels to meet the guidelines by age 55, to gain the most benefits for physical health in later life.

## Abstract 2

Binh Nguyen and colleagues, use longitudinally collected data and target trial emulation methods to explore the impact of mid-life physical activity on the health outcomes of Australian women later in life.

## Author summary

**Why was this study done?:**

○ The evidence for an association between physical activity and health-related quality of life has been based primarily on cross-sectional studies and randomized controlled trials (RCTs) with short-term outcomes.
  ○ To date, few longitudinal studies have examined the associations between long-term patterns of physical activity and health-related quality of life, measured physical activity at more than one time point, and examined the causal effects of physical activity.

**What did the researchers do and find?:**

○ We leveraged data on >11,000 women enrolled in the Australian Longitudinal Study on Women’s Health (ALSWH) and applied targeted maximum likelihood estimation (TMLE) to emulate various target trials of physical adherence regimes and explore how physical activity in midlife impacts health outcomes in later life.
  ○ We found that consistent adherence to physical activity guidelines over 15 years was associated with better health-related quality of life in the physical health domain, especially physical functioning.
  ○ Meeting physical activity guidelines earlier in midlife and especially by age 55 resulted in better physical health-related quality of life outcomes in later life but the same impact on mental health domain outcomes was less evident.

**What do these findings mean?:**

○ This study provides insights into the long-term effects of different patterns of physical activity during mid-age on later health-related quality of life in Australian women.
○ Combined with existing evidence, it contributes to the growing evidence concerning the benefits of maintaining or adopting an active lifestyle in mid-age.
○ The study presents an important message for public health which encourages women to be active throughout mid-age, even if they start meeting physical activity guidelines in their mid-50s, to gain the most benefits for physical health-related quality of life latterly.
○ Limitations of the study included reliance on self-reported physical activity, an inability to account for all contributing factors and findings may not be generalizable to all mid-age Australian women or to women outside of Australia.

## Introduction

The health benefits of physical activity are well established [1–4]. The World Health Organization (WHO) recommends 150 to 300 min of moderate-intensity aerobic physical activity or 75 to 150 min of vigorous activity per week (or an equivalent combination of both) for health benefits [2]. Although maintaining an active lifestyle is optimal for health, levels of physical activity may change across the lifespan. For women, major transitions in life, such as marriage, childbirth, children leaving home, retirement, and bereavement, can be associated with changes in physical activity [5]. To date, most studies on physical activity and health have relied on physical activity measured at one point in time, not accounting for changes in activity patterns over time. Relying on a single static measure not only introduces measurement biases but also fails to answer an important question “what would happen if one starts to be physically active in later life?” The health outcomes of longitudinal patterns of meeting physical activity guidelines in women have seldom been explored [6–9].

Physical activity is important for healthy aging, defined by the WHO as “the process of developing and maintaining the functional ability that enables wellbeing in older age” [10]. An important measure of healthy aging is health-related quality of life, a multidimensional concept that captures perceived functioning and wellbeing in the physical, mental, and social domains of health [11]. Health-related quality of life is a strong predictor of mortality risk [12] and is widely used as a population health indicator [13]. Evidence suggests a link between physical activity and health-related quality of life in adults, but this evidence is primarily based on cross-sectional studies, which have inherent biases, and randomized controlled trials (RCTs), which tend to focus on short-term outcomes [14,15]. Few studies have examined the associations between long-term patterns of physical activity and health-related quality of life [16]. As it is impractical to conduct a physical activity RCT over many years, we use causal inference methods to emulate a long-term RCT from observational data [17,18]. This analysis approach can produce valid causal inferences under some assumptions [19]. Previous studies have not considered complex forms of bias that can arise in longitudinal data or examined causal effects.

The aim of this study was to examine the associations between longitudinal patterns of physical activity over 15 years and health-related quality of life, including physical and mental health domains and individual subscales, in a cohort of middle-aged Australian women. We hypothesized that adherence to physical activity guidelines in mid-age would lead to improved quality of life, in both physical and mental health domains. Specifically, we used target trial emulation [20] to test the following hypotheses:

1. Consistently meeting physical activity guidelines in all surveys of the exposure period (2001 to 2016, when women aged from 50–55 to 65–70) (“consistent adherence” thereafter) will result in better health-related quality of life in 2019 (when women aged 68 to 73) than not meeting guidelines in any survey (control: “consistent non-adherence”).
2. Starting to meet guidelines during the exposure period will result in better health-related quality of life than the control condition of consistent non-adherence, with earlier initiation associated with better outcomes.

## Methods

### Target trial emulation

A target trial emulation approach enables the use of observational data to emulate a “target” trial (i.e., the RCT that would have been conducted, were it possible to do so), with exposures framed as hypothetical interventions (e.g., the intervention group following a particular pattern of physical activity over the treatment/exposure period) [20]. We emulated a target trial in which the “intervention” was conceptualized as participants being assigned to meet the guidelines following different patterns across the course of the study with 100% adherence, compared with a control defined as meeting guidelines in none of the surveys (Table 1).

**Table 1:** Comparison of target trial and emulated trial using ALSWH data.

	Target trial	Emulation with ALSWH
Eligibility criteria	Women aged 47–52 years with unimpaired physical functioning and prepared to accept an intervention to meet physical activity guidelines	Women in the ALSWH cohort aged 47–52 years with unimpaired physical functioning who either did or did not meet physical activity guidelines
Trial arms	Intervention: Women adhering to meet physical activity guidelines over a 15-year period	Intervention: Women meeting physical activity guidelines over a 15-year period (referred to hereafter as “exposed”)
	Control: Individuals caused to not meet physical activity guidelines	Control: Individuals not meeting physical activity guidelines
Assignment procedures	Random assignment to either intervention or control arm at recruitment	Adjustment for confounding using TMLE
Follow-up period	15 years of intervention period (6 triennial waves of follow-up), plus 3 years postexposure for measurement of outcome	15 years of exposure (6 triennial waves of follow-up), plus 3 years postexposure for measurement of outcome
Outcomes	Health-related quality of life assessed using the SF-36	Health-related quality of life assessed using the SF-36
Causal effect measure	The difference in means of outcomes in the intervention versus the control arms of the study	ATE estimated as the difference in conditional expected mean under treatment versus conditional mean under control

ALSWH, Australian Longitudinal Study on Women’s Health; ATE, average treatment effect; SF-36, 36-item Medical Outcomes Study short-form survey; TMLE, targeted maximum likelihood estimation.

### Study population

**Fig 1:**
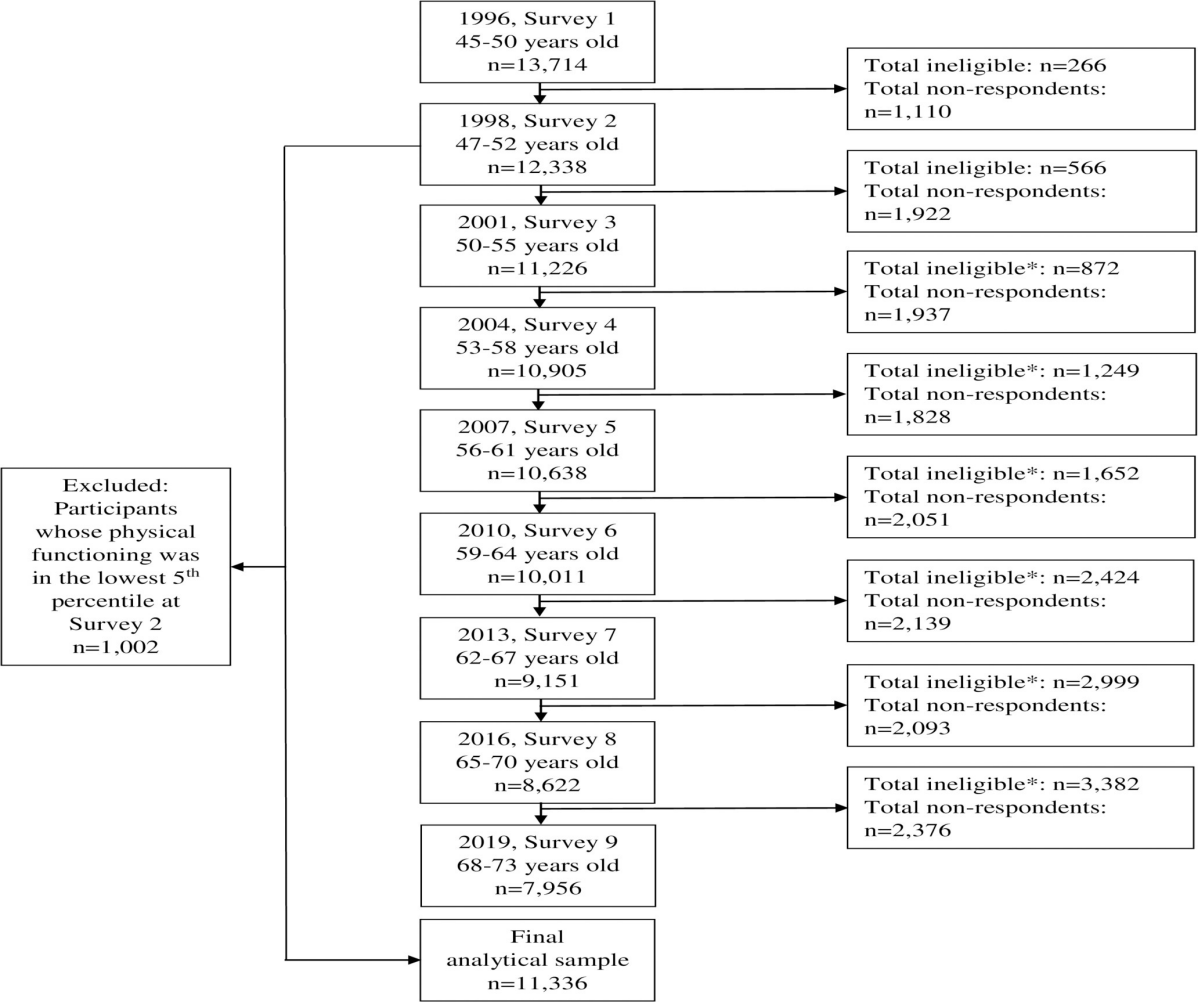
Study flow chart. *Ineligible comprises: deceased, frailty and withdrawn.

We used data from the 9 surveys (1996 to 2019) of the 1946 to 1951 birth cohort of the Australian Longitudinal Study on Women’s Health (ALSWH), a population-based prospective cohort study. More information can be found on the ALSWH website (http://www.alswh.org.au), and details of the sample, study design, and recruitment are provided elsewhere [21,22]. The analytical sample includes 11,336 women born in 1946 to 1951 who were 45 to 50 years of age when they first completed the baseline survey in 1996. Women were randomly selected from the Medicare Australia national insurance database and followed up approximately every 3 years (1998, 2001, 2004, 2007, 2010, 2013, 2016, and 2019) with mailed surveys offered initially and web-based surveys more recently. The response rate for initial recruitment was estimated to be 53% to 56% [21]. The ALSWH study has ongoing ethical approval from the Human Research Ethics Committees of the Universities of Newcastle and Queensland (approval numbers H-076-0795 and 2004000224). All participants provided signed consent before participation. Participants whose physical functioning was in the lowest 5th percentile at the study “baseline” in 1998 (immediately prior to the exposure period) were excluded from this study as their physical functioning may have been too impaired to participate in physical activity. The Strengthening the Reporting of Observational Studies in Epidemiology (STROBE) checklist is included in S1 STROBE Checklist, and study flow chart is presented in Fig 1.

### Outcome variables

The primary outcomes are the physical health component (PCS) and mental health component (MCS) summary scores, derived from the 36-item Medical Outcomes Study short-form survey (SF-36) [23], a validated questionnaire covering various aspects of health-related quality of life. All except one of the 36 items were used to define 8 subscales: general health, physical functioning, physical role (role limitations from physical problems), bodily pain, vitality, social functioning, emotional role (role limitations from emotional problems), and mental health. These subscales were standardized and combined to form the PCS and MCS [24]. Scores range between 0 and 100 and higher scores indicate higher perceived physical/mental health. For this analysis, scales were standardized against data for Australian women of a similar age (age 45 to 54 years) [25]. We used the individual subscale scores as secondary outcomes. All outcomes were from survey 9 (2019) of the study.

### Exposure measurement

Physical activity was measured using a validated and reliable modified version of the Active Australia survey questions [26]. Reported weekly minutes of activity (walking, moderate leisure activity, and vigorous leisure activity) were summed, with vigorous activity weighted double [27]. We dichotomized total physical activity based on reports of at least 150 min of weighted activity, as recommended by the WHO [28]. The exposure window was defined as a 15-year period, from 2001 to 2016, corresponding to study surveys 3 to 8. We did not use surveys 1 and 2 because the physical activity questions were different.

#### Emulated interventions

We emulated the following 2 interventions against the control of consistent non-adherence to physical activity guidelines:

1. Consistent adherence to guidelines.
2. Starting to meet the guidelines on reaching the ages of 55, 60, or 65. Age interventions were distinct from each other and not cumulative.

More details on the methods used to define and evaluate the intervention regimes are included in S1 Text.

### Confounders

To illustrate the expected causal relationships among confounders, exposures, and outcomes over time, we developed a directed acyclic graph (DAG) (S1A and S1B Fig). A DAG is a conceptual presentation of the causal relationships between variables and can be used to inform research design and analysis [29]. In the case of the current study, the DAG helped us select confounders that could be common causes of both exposures and outcomes but not an intermediate outcome on the causal pathway (as the confounders were collected prior to the exposure). Baseline time-constant confounders, including highest level of education completed and country of birth, were from surveys 1 (1996) and 2 (1998). Time-varying confounders were from surveys 2 to 7 (1998 to 2016), including age (continuous), employment status (employed; not employed), area-level socioeconomic status, measured using the Index of Relative Socio-economic Disadvantage (IRSD) [30] (tertiles), geographical remoteness based on the postcode-level Accessibility-Remoteness Index of Australia Plus (ARIA+) [31] (major city; regional; remote), living with children (yes; no), marital status (married/de facto; separated/divorced/never married), diagnosis/treatment history coronary heart disease, stroke, arthritis, any cancer, anxiety, and depression (all yes; no), Center for Epidemiological Studies-Depression (CES-D) scale [32] (continuous), perceived stress scale [33] (continuous), SF-36 subscale scores, body mass index (BMI) (underweight; healthy; overweight; obese), lifetime risky alcohol consumption (>10 alcoholic drinks/week) based on the 2020 National Health Medical Research Council guidelines [34] (yes; no), heavy episodic alcohol consumption (more than 4 drinks on an occasion at least once a month) (yes; no), smoking status (never smoker; ex-smoker; current smoker), vegetable intake (<1; 1; 2; 3; 4; 5; ≥6 vegetables/day), and fruit intake (0; <1; 1; 2; 3; ≥4 pieces of fruit/day). S2 Text presents detail of the confounders.

### Statistical analysis

We preregistered the analysis (https://doi.org/10.17605/OSF.IO/24T6Z). We used models to emulate interventions, following the counterfactual framework, where each participant is considered to have a number of potential outcomes (corresponding to different exposures), only one of which is observed (because each person can only have 1 actual exposure history). Analytically, counterfactual methods use all available information to estimate the expected outcome had all participants followed a particular exposure pattern. More details about the counterfactual framework and its implementation are included in S1 Text.

Because the exposure group (e.g., adherence to physical activity guidelines) in the ALSWH is not randomly allocated, emulating the target trial requires controlling for confounding [35]. Because some of the confounding variables may themselves be affected by past exposure (e.g., *BMI_t−1_* is a confounder of the exposure time t, but the exposure may also likely affect subsequent observations of BMI; as indicated in S1A Fig by the arrows connecting *Exposure_t_* to *Time−varying confounders_t_*), specialized analytical methods are required to produce unbiased causal effect estimates [19]. As such, we conducted all analyses using targeted maximum likelihood estimation (TMLE) [36], a consistent, doubly robust method for estimating causal effects, providing valid causal inference under the assumptions of conditional exchangeability [37], positivity [38], consistency [39], and no interference [40]. We conducted all analysis in R 4.2.1 via the “ltmle” package with models estimated via ensemble machine learning [41]. All analyses were weighted based on the probability of selection into the study [21]. Results are reported as expected mean scores, as well as mean difference versus control, and their 99.5% confidence intervals (CIs). This conservative alpha level (0.005) was selected based on the recommendations by Benjamin and colleagues [42]. Standard errors were estimated using the higher of the TMLE or influence-curve variance. Further details of the estimation methods and target estimands are included in S1 Text. Analysis code is available online (https://www.philipclare.com/code/alswh).

#### Sensitivity analyses

Additionally, we conducted 5 sets of sensitivity analyses:

1. We tested the hypothetical interventions of “starting the exposure period meeting the guidelines but stopping meeting them upon reaching 55, 60, and 65 years.”
2. Analyses using a lower cut-point of 75 min instead of 150, to address the generally curvilinear relationship between physical activity and health-related outcomes, in which being active below the recommended levels could convey significant health benefits [2,3].
3. Analyses using a higher cut-point of 300 min to reflect the upper cut-point of the physical activity guidelines.
4. Analyses excluding a small number of variables that were wholly missing in a given survey because they were not included in that survey wave (S3 Text).
5. E-value analysis (S4 Text) to assess the potential impact of unmeasured confounding [43].
6. Post hoc sensitivity analyses excluding mental health confounders (depression, anxiety, and stress) in order to test for possible overadjustment.

#### Missing data

The data contained missing values from 2 scenarios: (1) intermittent missing data within surveys due to non-response to individual questions; and (2) missing data due to non-response to any entire survey (including loss to follow-up defined as those who did not complete a survey wave (or subsequent survey waves) of the study). The proportion of missing physical activity data ranged from 16% (survey 3) to 34% (survey 8) (Table A in S3 Text). The median (interquartile range) number of waves completed was 5 (3, 7). We handled intermittent missing data using multiple imputation [19] using chained equations, with M = 40 imputations [19], and data imputed using random forests [19]. Missing waves was handled using inverse probability of censoring weights [16]. Further details of the missing data and the procedures used to address them are included in S3 Text. A post hoc sensitivity analysis was subsequently conducted in which both intermittent missing data and missing waves were imputed, and the main analyses were conducted again using that version of the data (S7 Text).

## Results

### Sample characteristics

Characteristics of the weighted sample (*n* = 11,336) are presented in Table 2. Characteristics of the unweighted sample are presented in Table A in S5 Text. At baseline (1996), participants were aged 45 to 50 years (mean: 49.5 years; standard deviation [SD] 1.5). Three quarters of participants were born in Australia and almost two thirds had up to high school education only. Most participants were employed, married, or in a de facto relationship, and more than half lived with children (60.5%). Participants were relatively equally distributed across area-level socioeconomic status. More than half lived in regional areas and nearly a third in major cities. One in 8 were lifetime risky drinkers, almost one third were heavy episodic drinkers, and almost 1 in 6 were current smokers. Forty five percent was overweight or obese.

**Table 2:** Descriptive statistics of the weighted analytical sample of Australian women at baseline.

Variable	Categories	Mean (SD)/ *n* (%) (*N* = 11,336)
**Age**		49.5 (1.8)
**Highest level of education**	High school or less	6,878 (60.0%)
	Trade/apprentice/certificate/ diploma	2,280 (20.0%)
	University	1,968 (20.0%)
**Country of birth**	Australia	7,953 (70.0%)
	Other	3,155 (30.0%)
**Employment status**	Not employed	2,130 (20.0%)
	Employed	8,976 (79.2%)
**Marital status**	Married/de facto	9,094 (80.0%)
	Separated/divorced/never married	1,813 (20.0%)
	Widowed	257 (0.0%)
**Live with children under 18 years**	No	6,693 (70.0%)
	Yes	3,476 (30.0%)
**Live with children aged 18+ years**	No	5,928 (60.0%)
	Yes	4,299 (40.0%)
**Area-level SES (IRSD)**	Score for lowest tertile	939 (72)
	Score for middle tertile	985 (24)
	Score for highest tertile	1,071 (82)
**Remoteness (ARIA+)**	Major city	7,277 (70.0%)
	Regional	3,680 (30.0%)
	Remote	235 (0.0%)
**Lifetime risky drinking** ^**a**^	No	8,814 (90.0%)
	Yes	1,395 (10.0%)
**Heavy episodic drinking** ^**b**^	No	7,007 (70.0%)
	Yes	3,463 (30.0%)
**Smoking status**	Never smoker	5,961 (60.0%)
	Ex smoker	2,830 (30.0%)
	Current smoker	1,689 (20.0%)
**CESD-10 Depression Score**		6.2 (7.1)
**Mean stress score**		0.6 (0.7)
**BMI category**	Underweight	144 (0.0%)
	Healthy	4,877 (50.0%)
	Overweight	3,032 (30.0%)
	Obese	1,783 (20.0%)
**Ever diagnosed/treated for heart**	No	11,013 (100.0%)
**disease**	Yes	216 (0.0%)
**Ever diagnosed/treated for stroke**	No	11,148 (100.0%)
	Yes	82 (0.0%)
**Ever diagnosed/treated for cancer**	No	10,766 (100.0%)
	Yes	464 (0.0%)
**Ever diagnosed/treated for depression**	No	9,377 (80.0%)
	Yes	1,853 (20.0%)
**Ever diagnosed/treated for anxiety**	No	9,704 (90.0%)
	Yes	1,526 (10.0%)
**SF-36 scores**	Physical component score	51.14 (9.50)
	Mental component score	47.51 (15.22)
	Physical functioning	89.44 (14.27)
	Role physical	83.98 (39.39)
	Bodily pain	73.22 (27.60)
	General health	74.59 (23.38)
	Vitality	60.03 (24.95)
	Social functioning	84.27 (27.07)
	Role emotional	79.61 (43.49)
	Mental health	73.77 (21.42)

Note: percentages calculated as percent of total sample; percentages do not add to 100% due to missing data within variables.

^a^Lifetime risky alcohol consumption defined as >10 alcoholic drinks/week based on the 2020 National Health Medical Research Council guidelines [34].

^b^Heavy episodic alcohol consumption defined as >4 alcoholic drinks on an occasion at least once a month [34].

ARIA+, Accessibility-Remoteness Index of Australia Plus; BMI, body mass index; CESD-10, 10-item Centre for Epidemiological Studies Depression Scale; IRSD, Index of Relative Socio-economic Disadvantage; SD, standard deviation; SF-36: 36-item Medical Outcomes Study short-form survey.

### Main analyses

**Fig 2:**
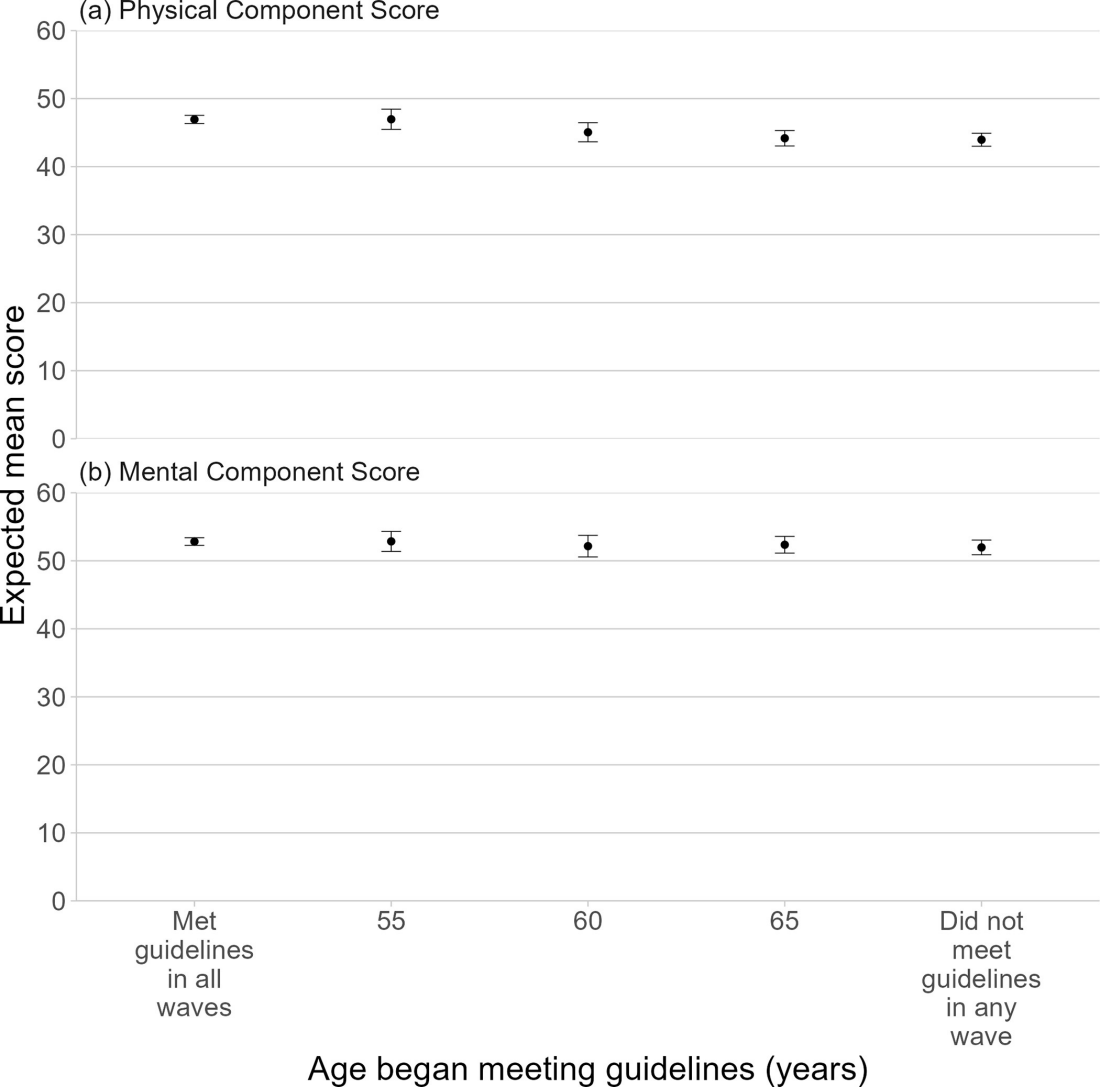
Primary analysis of the effect of age at which started to meet physical activity guidelines on SF-36 component scores. Abbreviations: SF-36, 36-item Medical Outcomes Study short-form survey. This figure shows the effect of meeting physical activity upon reaching a particular age (“starters”) but not prior to that, with a range of age thresholds considered (55, 60, and 65 years) on the physical health component and mental health component scores at survey 9. The points represent the estimates and the bars the 99.5% confidence intervals. Models were adjusted for: highest level of education, country of birth, age, employment status, living with children, marital status, SEIFA IRSD, geographical remoteness (ARIA+), history of coronary heart disease diagnosis/treatment, history of stroke diagnosis/treatment, history of arthritis diagnosis/treatment, history of any cancer diagnosis/treatment, history of anxiety diagnosis/treatment, and history of depression diagnosis/treatment, CES-D scale, stress, SF-36 subscale scores, BMI, lifetime risky alcohol consumption based on the 2020 National Health Medical Research Council guidelines, heavy episodic alcohol consumption, smoking status, vegetable intake, and fruit intake. ARIA+, Accessibility-Remoteness Index of Australia Plus; BMI, body mass index; CES-D, Center for Epidemiological Studies-Depression; SEIFA IRSD, Socio-economic Index for Areas Index of Relative Socio-economic Disadvantage.

Consistent adherence to physical activity guidelines (PCS: 46.93 [99.5% CI: 46.32, 47.54]) was associated with three-point higher PCS (mean difference: 3.0 [99.5% CI: 1.8, 4.1]) than the control (consistent non-adherence, PCS: 43.90 [42.79, 45.01]) (Fig 2 and Table A in S6 Text). While starting to meet guidelines upon reaching 55 years was associated with a similarly higher PCS (46.96 [45.53, 48.40]; mean difference versus control: 3.0 [99.5% CI: 1.2, 4.8]), starting to meet guidelines upon reaching 60 (mean difference versus control: 1.1 [99.5% CI: −0.6, 2.8]) and 65 (mean difference versus control: 0.2 [99.5% CI: −0.9, 1.3]) were not associated with statistically different PCS compared with the control. In terms of subscales (Tables B and J and Fig A in S6 Text), analyses show 5- to 9-point differences between consistent adherence to guidelines and consistent non-adherence in all 4 physical subscales, with the largest effect observed for “physical functioning” (mean difference: 9.3 [99.5% CI: 6.2, 12.3]). For all 4 physical health subscales, there was a similar magnitude of difference for starting to meet guidelines on reaching age 55 compared with the control, but no evidence for meaningful difference for starting to meet the guidelines on reaching age 60 and 65.

We found no statistically significant differences in MCS between any hypothetical intervention and the control. In terms of mental subscales, consistent adherence to guidelines resulted in statistically higher scores for “vitality” (5-point difference). We also found a significant difference in “vitality” (around 5-point differences) linked to meeting guidelines on reaching 55 years. However, we did not find statistically significant differences between starting to meet guidelines on reaching 60 and 65, compared with the control, for any of the mental subscales.

### Sensitivity analyses

Tables A and B and Figs B and C in S6 Text outlined the potential interventions of stopping meeting guidelines at ages 55, 60, and 65. Starting the exposure period meeting the guidelines, but then stopping meeting them on reaching age 65 was associated with nearly a three-point higher PCS than consistent non-adherence. The higher PCS was similar in magnitude to that associated with consistent adherence. Stopping meeting guidelines on reaching 55 or 60 years was not associated with statistically different PCS from the control. There were no statistically significant differences in MCS between any hypothetical intervention and the control.

In sensitivity analyses using a lower threshold of 75 min per week of physical activity, patterns for the primary (Table C and Figs D and E in S6 Text) and secondary analyses (Table D and Figs F and G in S6 Text) were similar, but differences were less marked. In sensitivity analyses using a higher threshold of 300 min per week of physical activity, patterns for the primary (Table E and Figs H and I in S6 Text) and secondary analyses were similar (Table F and Figs J and K in S6 Text). Findings from sensitivity analyses excluding confounders in surveys where they were completely missing (Tables G and H and Figs L–O in S6 Text) were similar to those from the main analyses.

The results of the E-Value analysis suggest that a moderate amount of unmeasured confounding would be required to alter the interpretation of the results (Tables I and J in S6 Text), with unmeasured confounding needing to double (or halve) the risk of the exposure in order to alter the significant findings reported.

Post hoc sensitivity analyses excluding mental health confounders returned very similar results to the primary analysis, suggesting that results were not due to overadjustment (Figs P–S in S6 Text). The unplanned post hoc sensitivity analyses where both intermittent missing data and missing waves were imputed showed findings similar to those from the main analyses (Table A in S7 Text).

## Discussion

Overall, the findings from this study highlight the importance of being physically active for as long as possible to gain the most benefits for physical health. Our findings show that consistent adherence to physical activity guidelines over 15 years was associated with better health-related quality of life in the physical health domain (three-point higher PCS on a scale of 1 to 100), compared with not adhering to guidelines in all surveys. Even partial adherence to guidelines during the 15 years, namely starting to meet the guidelines on reaching age 55 and maintaining meeting the guidelines from the start of the exposure period until reaching 65, was associated with better physical health-related quality of life at around age 70. Consistent or partial adherence to physical activity guidelines seemed to have weaker effects on the mental health domain, with some significant differences observed in subscales only, but not with the overall MCS.

The magnitude of benefits regarding the physical health domain, either from consistent adherence to or earlier adoption or longer-term maintenance of meeting physical activity guidelines, is small but meaningful. Physical functioning is critical for maintaining independent functional ability, which is a predictor of various health outcomes including disability, hospitalization, and mortality [44,45]. For example, a 10-point difference in physical functioning scores may be interpreted as the difference between those with and without mild osteoarthritis [46]. A recent meta-analysis has found that even a one-unit increases in SF-36 physical functioning and the PCS are associated with lower risks of mortality in the general population [15].

To date, few studies have examined longitudinal associations between physical activity at multiple time points and health-related quality of life [16,47]. Previous analyses of data from the 1946 to 1951 ALSWH cohort, using the 6-year period from surveys 3 to 5, found small improvements in both PCS and MCS, but meaningful improvements in physical functioning and vitality with increases in total physical activity from 2001 to 2007 [16]. Analyses of data from the Nurses’ Health Study also showed associations between increasing physical activity over 10 years from 1986 to 1996 and improvements in health-related quality of life scores (eight-point improvement in physical function, four-point improvement in vitality, and a two-point improvement in the mental health subscale) [47]. The magnitude of the differences in health-related quality of life scores in our data were similar in the mental domain to those reported in these earlier papers [16,47] and similar in the physical domain to those reported from the Nurses’ Health Study [47]. The differences in PCS and physical functioning scores were higher in our study than in the previous 6-year analysis of data from this ALSWH cohort [16,47]. However, it is important to note that our analyses were not directly comparable with these earlier studies because we did not compare women with different physical activity patterns, but rather, the expected differences in health-related quality of life if all women followed a specific intervention during the exposure period. Therefore, using a causal inference framework, our analyses assumed that women with different physical activity interventions and the control are the same (“exchangeable”) with respect to observed confounders, except for their physical activity levels.

Our modeling of dynamic interventions defined by women changing physical activity in midlife is based on the consideration that some women may not have been physically active enough until their fifties. It is therefore important to understand whether adopting an active lifestyle in midlife still conveys benefits. Our findings suggest that to maintain good physical health-related quality of life at around age 70, one may be able to “make up” for not being active earlier by becoming active in the mid-50s. This finding supports public health initiatives for messaging around “turning back the clock” in midlife through lifestyle changes such as physical activity. The nonsignificant findings about being physically active only at age 60 or 65 may not be interpreted as the window opportunity being closed, but rather, that there had not been sufficient accumulation of physical activity for the health benefits to be evident by around age 70.

The main strengths of the study are the large cohort and the repeated physical activity measures over a long exposure period. Another strength is that we accounted for a wide range of potential time-varying confounders, using robust causal inference methods. Provided structural assumptions hold (positivity, no interference, consistency, and no unmeasured confounding), these methods allow us to estimate causal effects rather than associations, although even if those assumptions cannot be relied upon, these methods provide at least as reliable estimates as methods that only examine associations. In this case, the assumptions of positivity, no interference and consistency at least appeared reasonable. Meeting physical activity guidelines is common enough that there were no positivity violations, and TMLE is robust to near-positivity violations. Because the study uses data from a population cohort, and is based on clear and unambiguous definitions of treatment/control, there is little chance of violations of either no interference or consistency. Unmeasured confounding is possible despite adjusting for health variables and conditions, and excluding data from women with poor physical functioning, we cannot guarantee that the study controls for all confounders for the relationship between physical activity and health-related quality of life, such as underlying health conditions and/or other unmeasured or insufficiently measured confounders. However, E-Value analysis suggests that the key differences observed would require fairly substantial unmeasured confounding to result in different conclusions (requiring a 50%+ change in exposure and outcome in order to affect the conclusions in most cases). Another limitation is the use of self-reported physical activity; however, this measure has good reliability and validity [26]. In this study, we could not emulate all possible interventions. Finally, although the ALSWH included nationally representative samples at baseline [21], healthier women have remained in the study, so the findings may not be generalizable to all mid-age Australian women [48].

In conclusion, this study contributes to our understanding of the prospective associations between physical activity and health-related quality of life in mid-age women. An important public health message is that being active for as many years as possible, even if women start to meet physical activity guidelines in their mid-50s, could have important health benefits in terms of physical health, especially in physical functioning. Our study contributes to growing evidence of the health benefits of maintaining or adopting an active lifestyle in mid-age. Such public health messages should be used to encourage middle-aged women to become and stay active.

## Supporting information

S1 STROBE Checklist Strengthening the Reporting of Observational Studies in Epidemiology (STROBE) checklist. (DOCX)

S1 Text Model estimands. (DOCX)

S2 Text Confounding variables. (DOCX)

S3 Text Missing data. (DOCX)

S4 Text E-value analysis. (DOCX)

S5 Text Descriptive statistics of the unweighted analytical sample. (DOCX)

S6 Text Additional results. (DOCX)

S7 Text Post hoc sensitivity analysis findings. (DOCX)

S1 Fig Directed acyclic graph. (DOCX)

## Abbreviations

ALSWH: Australian Longitudinal Study on Women’s Health
ARIA+: Accessibility-Remoteness Index of Australia Plus
BMI: body mass index
CES-D: Center for Epidemiological Studies-Depression
CI: confidence interval
DAG: directed acyclic graph
IRSD: Index of Relative Socio-economic Disadvantage
MCS: mental health component summary
PCS: physical health component summary
RCT: randomized controlled tria
SD: standard deviation
TMLE: targeted maximum likelihood estimation
WHO: World Health Organization
