# Supplementary material for: Physical activity across midlife and health-related quality of life in Australian women: A target trial emulation using a longitudinal cohort
Source: PLoS Med. 2024 May 2;21(5):e1004384. doi: 10.1371/journal.pmed.1004384 (PMC11065283; doi:10.1371/journal.pmed.1004384)
Supplement: S1 STROBE Checklist — (DOCX) [file pmed.1004384.s001.docx]

# S1 STROBE Checklist

Strengthening the Reporting of Observational Studies in Epidemiology (STROBE) checklist.

| **Item** | **Item No.** | **Recommendation** | **Section** |
| --- | --- | --- | --- |
| Title and abstract | 1 | (*a*) Indicate the study’s design with a commonly used term in the title or the abstract | Title page |
|  |  | (*b*) Provide in the abstract an informative and balanced summary of what was done and what was found | Abstract |
| Introduction | | | |
| Background/rationale | 2 | Explain the scientific background and rationale for the investigation being reported | Paragraphs 1 and 2 |
| Objectives | 3 | State specific objectives, including any prespecified hypotheses | Paragraph 3 |
| Methods | | | |
| Study design | 4 | Present key elements of study design early in the paper | Paragraph 1 |
| Setting | 5 | Describe the setting, locations, and relevant dates, including periods of recruitment, exposure, follow-up, and data collection | Study population |
| Participants | 6 | (*a*) Give the eligibility criteria, and the sources and methods of selection of participants. Describe methods of follow-up | Study population |
|  |  | (*b*) For matched studies, give matching criteria and number of exposed and unexposed |  |
| Variables | 7 | Clearly define all outcomes, exposures, predictors, potential confounders, and effect modifiers. Give diagnostic criteria, if applicable | Outcome variables, exposure measurement, confounders |
| Data sources/ measurement | 8* | For each variable of interest, give sources of data and details of methods of assessment (measurement). Describe comparability of assessment methods if there is more than one group | Same sections as above |
| Bias | 9 | Describe any efforts to address potential sources of bias | Statistical analysis |
| Study size | 10 | Explain how the study size was arrived at | Study population |
| Quantitative variables | 11 | Explain how quantitative variables were handled in the analyses. If applicable, describe which groupings were chosen and why | Outcome variables, exposure measurement, confounders, statistical analysis |
| Statistical methods | 12 | (*a*) Describe all statistical methods, including those used to control for confounding | Statistical analysis |
|  |  | (*b*) Describe any methods used to examine subgroups and interactions |  |
|  |  | (*c*) Explain how missing data were addressed | Statistical analysis |
|  |  | (*d*) If applicable, explain how loss to follow-up was addressed | Statistical analysis |
|  |  | (*e*) Describe any sensitivity analyses | Statistical analysis |
| Results | | | |
| Participants | 13* | (a) Report numbers of individuals at each stage of study—eg numbers potentially eligible, examined for eligibility, confirmed eligible, included in the study, completing follow-up, and analysed | Figure 1 |
|  |  | (b) Give reasons for non-participation at each stage | Figure 1 |
|  |  | (c) Consider use of a flow diagram | Figure 1 |
| Descriptive data | 14* | (a) Give characteristics of study participants (eg demographic, clinical, social) and information on exposures and potential confounders | Sample characteristics |
|  |  | (b) Indicate number of participants with missing data for each variable of interest | S3 Text |
|  |  | (c) Summarise follow-up time (eg, average and total amount) |  |
| Outcome data | 15* | Report numbers of outcome events or summary measures over time |  |
| Main results | 16 | (*a*) Give unadjusted estimates and, if applicable, confounder-adjusted estimates and their precision (eg, 95% confidence interval). Make clear which confounders were adjusted for and why they were included | Results (paragraphs 2 and 3), Tables and Figures, S6 Text |
|  |  | (*b*) Report category boundaries when continuous variables were categorized |  |
|  |  | (*c*) If relevant, consider translating estimates of relative risk into absolute risk for a meaningful time period |  |
| Other analyses | 17 | Report other analyses done—eg analyses of subgroups and interactions, and sensitivity analyses | Sensitivity analyses |
| Discussion | | | |
| Key results | 18 | Summarise key results with reference to study objectives | Paragraph 1 |
| Limitations | 19 | Discuss limitations of the study, taking into account sources of potential bias or imprecision. Discuss both direction and magnitude of any potential bias | Strengths and limitations |
| Interpretation | 20 | Give a cautious overall interpretation of results considering objectives, limitations, multiplicity of analyses, results from similar studies, and other relevant evidence | Discussion |
| Generalisability | 21 | Discuss the generalisability (external validity) of the study results | Strengths and limitations |
| Other information |  | | |
| Funding | 22 | Give the source of funding and the role of the funders for the present study and, if applicable, for the original study on which the present article is based | Funding statement |
