# Supplementary material for: Physical activity across midlife and health-related quality of life in Australian women: A target trial emulation using a longitudinal cohort
Source: PLoS Med. 2024 May 2;21(5):e1004384. doi: 10.1371/journal.pmed.1004384 (PMC11065283; doi:10.1371/journal.pmed.1004384)
Supplement: S1 Text — (DOCX) [file pmed.1004384.s002.docx]

# S1 Text

# Model estimands

## Counterfactuals

The primary estimands will be the expectations of the outcomes, under each counterfactual pattern of exposure based on each treatment regime d, marginal with respect to observed confounders. That is, for each regime t, we estimate the intervention specific mean $E_{0}\left( Y_{d} \right)$. We can then contrast intervention specific means under different regimes, $d$ and $d'$, to calculate additive effects equivalent to average treatment effects:

$ATE=E_{0}\left( Y_{d} \right)-E_{0}\left( Y_{d^{'}} \right)$. (*C-1*)

Initially, a static counterfactual exposure pattern will be evaluated – namely, the effect of meeting physical activity guidelines in all six waves of the exposure period, versus meeting guidelines in none of the six waves.

Subsequent to that, we will evaluate dynamic treatment effects, based on age thresholds in the wave prior to each exposure, for example:

$d\left( A_{t}=1 \right)=age_{t-1}>\theta$, *(C-2)*

where theta is the age threshold.

That is, we will evaluate the counterfactual effect of meeting physical activity guidelines upon reaching a particular age, but not prior to that, with a range of age thresholds considered (50, 55, 60, and 65 years). Because of the age of the cohort during the study, the initial static exposure pattern can also be considered in this framework as age thresholds at the minimum age in wave 2 (44.6 years), and the maximum age in wave 7 (68.4 years).

## Targeted maximum likelihood estimation

Targeted maximum likelihood estimation (TMLE) is a method for estimating causal effects [1] that is ‘doubly robust’ because it uses two component models but requires only one of the two to be correctly specified.

TMLE starts by estimating an initial expectation $Q_{n}^{0}\left( A,L \right)$of the outcome using maximum likelihood [2,3], similar to that created for the G-computation procedure (formula C-3), and a conditional expectation of the probability of exposure $g_{n}^{0}\left( A|L \right)$, equivalent to the propensity score model used by IPW (formula C-4).

$Q_{n}^{0}\left( A,L \right)=E\left( Y|A,L \right)$, (C-3)

$g_{n}^{0}\left( A|L \right)=logit\left( \frac{P\left( A|L \right)}{1-P\left( A|L \right)} \right)$, (C-4)

The propensity model is then used to create a ‘clever’ covariate $h$:

$h\left( A,L \right)=\frac{I\left( A=1 \right)}{g_{n}^{0}\left( 1 | L \right)}-\frac{I\left( A=0 \right)}{g_{n}^{0}\left( 0 | L \right)}$. (C-5)

The initial estimate of the outcome expectation $E\left( Y|A,L \right)$ is then ‘updated’ based on a function of the initial estimate and the clever covariate:

$Q_{n}^{*}\left( A,L \right)=Q_{n}^{0}\left( A,L \right)+\epsilon_{n}h\left( A,L \right)$, (C-6)

where $\epsilon_{n}$ is the coefficient of $h\left( A,L \right)$, and the coefficient of $Q_{n}^{0}\left( A,L \right)$ is constrained to be equal to one. The TMLE is then estimated by evaluating the final model at different treatment values,

$\theta_{n}^{TMLE}=\frac{1}{n}\sum_{i=1}^{n} Q_{n}^{*}\left( 1,L \right)-Q_{n}^{*}\left( 0,L \right)$. (C-7)

This estimator is equivalent to the G-computation estimator, except that the updated $Q_{n}^{*}\left( A,L \right)$ in place of the initial estimate $Q_{n}^{0}\left( A,L \right)$. In other words, TMLE is similar to the doubly robust method of using an IPTW outcome model to standardize the mean outcome via G-computation. This provides a targeted estimation of the target parameter (the effect of exposure), at the expense of bias in other parameters in the model (which are treated as nuisance parameters, and thus not reported).

Because TMLE is a substitution estimator, where initial models are used as information in the final model, but not directly used in the model itself, TMLE can be estimated using machine learning methods, and is commonly estimated using the Super Learner ensemble machine learning algorithm [4]. For this analysis, we estimated Q and g using a range of prediction algorithms: means, generalized linear models, generalized additive models [5], and random forests [6].

## Assumptions for Causal Inference

Marginal structural models, estimated via TMLE or otherwise, provide valid causal inference, under a set of structural assumption. These are:

1. Conditional exchangeability – often called ‘no unmeasured confounding’, this assumption requires that exposure assignment is conditional only on measured confounders [7];
2. Positivity – requires that all participants had at least some possibility of being exposed [8], although Targeted Maximum Likelihood Estimation has been shown to be more robust to at least near violations of positivity [9];
3. Consistency – that there is no case where the observed outcome and the potential outcome under the observed exposure are difference, which typically only occurs when the exposure is defined ambiguously [10]; and
4. No interference – that the exposure of every participant is independent from the outcome of the other participants [11].

References

1. van der Laan MJ, Rubin DB. Targeted maximum likelihood learning. The International Journal of Biostatistics. 2006;2(1).

2. Bembom O, Petersen ML, Rhee SY, Fessel WJ, Sinisi SE, Shafer RW, et al. Biomarker discovery using targeted maximum-likelihood estimation: application to the treatment of antiretroviral-resistant HIV infection. Statistics in Medicine. 2009;28(1):152-72.

3. Arnold B, Arana B, Mausezahl D, Hubbard A, Colford JM. Evaluation of a pre-existing, 3-year household water treatment and handwashing intervention in rural Guatemala. International Journal of Epidemiology. 2009;38(6):1651-61.

4. van der Laan MJ, Polley EC, Hubbard AE. Super learner. Stat Appl Genet Mol Biol. 2007;6:Article25.

5. Hastie TJ, Tibshirani RJ. Generalized Additive Models: Chapman and Hall/CRC; 1990.

6. Breiman L. Random Forests. Machine Learning. 2001;45(1):5-32.

7. Greenland S, Pearl J, Robins JM. Causal diagrams for epidemiologic research. Epidemiology. 1999;10(1):37-48.

8. Petersen ML, Porter KE, Gruber S, Wang Y, van der Laan MJ. Diagnosing and responding to violations in the positivity assumption. Statistical Methods in Medical Research. 2012;21(1):31-54.

9. Porter KE. The Relative Performance of Targeted Maximum Likelihood Estimators Under Violations of the Positivity Assumption: UC Berkeley; 2011.

10. Cole SR, Frangakis CE. Commentary: the consistency statement in causal inference: a definition or an assumption? Epidemiology. 2009;20(1):3-5.

11. Liu L, Hudgens MG, Becker-Dreps S. On inverse probability-weighted estimators in the presence of interference. Biometrika. 2016;103(4):829-42.
