# Supplementary material for: Physical activity across midlife and health-related quality of life in Australian women: A target trial emulation using a longitudinal cohort
Source: PLoS Med. 2024 May 2;21(5):e1004384. doi: 10.1371/journal.pmed.1004384 (PMC11065283; doi:10.1371/journal.pmed.1004384)
Supplement: S2 Text — (DOCX) [file pmed.1004384.s003.docx]

# S2 Text

## Time-varying confounders

### Employment status

At baseline, participants were asked: “Which of the following best describes your main employment status?”, with responses: in full time paid work, in part time or casual paid work, work without pay (e.g. in a family business), home duties only - no paid work, studying – no paid work, unemployed – looking for work, unpaid voluntary work, retired, unable to work due to sickness or injury, other (please specify online). From this variable, we derived a binary variable coded as: 1) employed and 2) not employed.

### Area-level socioeconomic status

We classified the area participants lived in based on Socio-Economic Indexes for Areas (SEIFA) Index of Relative Socio-Economic Disadvantage (IRSD) [1], a population index of the relative disadvantage of geographic areas, based on the Australian Census, and accounting for a range of different socioeconomic variables including household income, educational levels, occupational levels, rent and mortgage repayments, dwelling size and number of occupants and disability rates within the area [2].

### Remoteness

We defined geographical remoteness using the Accessibility-Remoteness Index of Australia Plus (ARIA+) [3], which is a remoteness classification system based on the The Australian statistical geography standard (ASGS) from the Australian Census [4]. Participants were classified based on their postcode of residence into three categories: urban; regional; or remote.

### Living with children

Participants were asked how many people they currently lived with, with separate responses for numbers of partner/spouse, children, parents/in-laws, other relatives, or other adults. From this question, we created two binary variables (no/yes) of whether the participant lived with children under 18 years of age, or aged 18+.

### Marital status

Participants were asked “What is your present marital status?”, with responses: married, de facto, separated, divorced, widowed, never married. From this variable, we coded a three-level categorical variable, coded: 1) married/de facto; 2) separated/divorced/never married; and 3) widowed.

### History of diagnosis/treatment

At baseline, participants were asked if they had ever been diagnosed or treated for a range of conditions including coronary heart disease, stroke, various forms of cancer, depression, and anxiety. In subsequent waves, they were asked a similar question about whether they had been diagnosed or treated in the last three years (equating to the time since the previous wave of the study). From these responses, we created binary variables (no/yes) of being diagnosed or treated for: 1) coronary heart disease; 2) stroke; 3) any form of cancer; 4) depression; or 5) anxiety. An additional variable about diagnosis/treatment for arthritis was included from wave 3 onwards (participants were not asked about arthritis at baseline).

### Depression

To assess symptoms of depression, participants were asked a shortened version of the Center for Epidemiologic Studies Depression (CES-D) Scale [5]. The reduced scale contains 10 items compared to the original 20, and has been shown to have equivalent reliability and validity [6]. The CESD-10 contains 10 items about symptoms of depression, each scored from 0 to 3, and summed to create a scale ranging from 0 to 30.

### Stress

Participants’ levels of stress was assessed using the Perceived Stress Questionnaire, a scale for the assessment of stress that was developed specifically for the younger cohort of the ALSWH [7] and adapted to be suitable in the older cohorts. The PSQ for this cohort contains 10 items, each of which is scored from ‘not at all stressed’ (0) to ‘extremely stressed’ (4). The mean of the 10 items is calculated to create a mean stress score ranging from 0 to 4.

### Body mass index

Participants were asked to self-report their height and weight. Responses to these questions were used to calculate BMI, based on the formula:

$BMI=\frac{kg}{m^{2}}$. (B-1)

We then used the calculated BMI to classify participants into one of four categories, based on World Health Organization guidelines: underweight (BMI<18.5), healthy weight (18.5≤BMI≤24.9), overweight (25.0≤BMI≤29.9), and obese (BMI≥30.0).

### Risky alcohol consumption

Participants were asked “how often do you usually drink alcohol?” and “on a day when you drink alcohol, how many drinks do you usually have?”. These variables were used to estimate average quantity of alcohol consumed each week, which was in turn used to create a dichotomous variable for lifetime risky alcohol consumption, based on the 2020 National Health Medical Research Council guidelines that state that alcohol consumption should be greater than 10 drinks per week to avoid risk of harm over the lifetime [8]. Participants were also asked “How often do you have five or more drinks of alcohol on one occasion?”, which was used to create a dichotomous variable for heavy episodic drinking (none/any).

### Smoking status

Participants were asked a series of questions about their current and past smoking behaviour, including “How often do you currently smoke cigarettes or any tobacco products?”. These questions were used to create a composite smoking variable with three levels: never smoker, ex-smoker, and current smoker.

### Fruit and vegetable intake

Participants were asked “How many serves of vegetables do you usually eat each day?”, and “How many serves of fruit do you usually eat each day?”, with possible responses of none, 1 serve, 2-3 served, 4 serves, and 5 serves or more. From these variables, two dichotomous variables were created for vegetable and fruit consumption. Vegetable consumption was dichotomized at 4+ serves a day (none to 3; 4 or more), while fruit consumption was dichotomized at 2+ serves a day (none to 1; 2 or more).

### Age

Because the ALSWH is a birth cohort study, age (or more precisely, date of birth) was obtained in order to assess eligibility to participate in the study. From this, participants’ age at each survey wave, at the date of survey completion. The was entered into analysis as a continuous variable (not integer rounded), to account for the fact that the gap between waves was not exactly the same for all participants.

## Time-constant confounders

### Education

At baseline, participants were asked the highest level of qualification they had completed, with responses from ‘no formal qualification’ through to ‘higher university degree’. These responses were coded into a three-level categorical variable, coded: 0) Less than high school; 1) Trade/apprentice/certificate/diploma; and 2) University. Because of the age of the cohort, which make it likely that only minimal changes in education would occur over time, education was considered a time-constant confounder.

### Country of birth

Participants were asked which country they were born in. From this, we coded a binary item based on whether the participant was born in Australia or not.

References

1. Australian Bureau of Statistics. Technical Paper: Socio-Economic Indexes for Areas (SEIFA). 2016.

2. Pink B. Information paper: An introduction to Socio-Economic Indexes for Areas (SEIFA) 2006. Information paper: An introduction to Socio-Economic Indexes for Areas (SEIFA) 2006. Canberra: Australian Bureau of Statistics, Commonwealth of Australia; 2008. Contract No.: ABS Catalogue No. 2039.0.

3. Glover JD, Tennant SK. Remote areas statistical geography in Australia: notes on the Accessibility/Remoteness Index for Australia (ARIA+ version): Public Health Information Development Unit, The University of Adelaide; 2003.

4. Australian Bureau of Statistics. The Australian statistical geography standard (ASGS) remoteness structure. Australian Bureau of Statistics Canberra; 2018.

5. Radloff LS. The CES-D scale: A self-report depression scale for research in the general population. Applied psychological measurement. 1977;1(3):385-401.

6. Andresen EM, Malmgren JA, Carter WB, Patrick DL. Screening for depression in well older adults: Evaluation of a short form of the CES-D. American journal of preventive medicine. 1994;10(2):77-84.

7. Bell S, Lee C. Development of the Perceived Stress Questionnaire for Young Women. Psychology, Health & Medicine. 2002;7(2):189-201.

8. Australian Government National Health and Medical Research Council. Australian guidelines to reduce health risks from drinking alcohol. 2020 [Available from: <https://www.nhmrc.gov.au/file/15923/download?token=t0Hrxdvq>.
