# Supplementary material for: Physical activity across midlife and health-related quality of life in Australian women: A target trial emulation using a longitudinal cohort
Source: PLoS Med. 2024 May 2;21(5):e1004384. doi: 10.1371/journal.pmed.1004384 (PMC11065283; doi:10.1371/journal.pmed.1004384)
Supplement: S3 Text — (DOCX) [file pmed.1004384.s004.docx]

# S3 Text

There were a number of different mechanisms for missing data in the ALSWH dataset:

- 1. Loss to follow-up;
  2. Questions not asked in some waves; and
  3. Intermittent missingness due to refusal to answer, not knowing, etc.

The amount of missing data in each analysis variable is shown in Table F1. The most common patterns of missing data are shown in Figure F1. Data was confirmed to be not missing completely at random via Little’s test. Because of this, we assumed the data was missing at random. Because ignoring missingness on both the outcome and exposure variables can introduce bias [1], we conducted all analyses primary using multiple imputation.

Based on past research [2], we first attempted to impute using the ‘just another variable’ approach, in which data is imputed in ‘wide’ form, with one record per individual, and each repeated measurement of the same variable imputed as a separate variable. However, this approach failed to converge.

Because of this, we imputed the data using chained equations with the data in long form, using the R package ‘mice’ [3]. To handle possible complexity in the data, we imputed all variables using random forests, from the package ‘ranger’ [4]. Based on the proportion of missing information in the data, we used M=40 imputations [5].

Analyses were then conducted on each imputed dataset, and combined using Rubin’s rules with the R package ‘Amelia’ [6].

As a sensitivity analysis, we also conducted analyses with variable imputed, but excluding those variables that were not asked in a specific wave. For example, questions about alcohol were not asked in wave 3 – in the primary analysis, this data is imputed, but in the sensitivity analysis, alcohol variables are included from wave 4 onwards, but not wave 3.

Table A Summary of missing data in each analysis variable.

| **Variable** | | **Missing data in variable (% of wave)** | | | | | | | |
| --- | --- | --- | --- | --- | --- | --- | --- | --- | --- |
|  |  | **Wave 1/2** | **Wave 3** | **Wave 4** | **Wave 5** | **Wave 6** | **Wave 7** | **Wave 8** | **Wave 9** |
| Outcomes | SF-36 Physical component score | 181 (2%) | 1723 (15%) | 1999 (18%) | 2209 (19%) | 2521 (22%) | 3341 (29%) | 3766 (33%) | 4299 (38%) |
|  | SF-36 Mental component score | 181 (2%) | 1723 (15%) | 1999 (18%) | 2209 (19%) | 2521 (22%) | 3341 (29%) | 3766 (33%) | 4299 (38%) |
|  | SF-36 Physical functioning | 65 (1%) | 1399 (12%) | 1722 (15%) | 1951 (17%) | 2451 (22%) | 3107 (27%) | 3571 (32%) | 4095 (36%) |
|  | SF-36 Role physical | 32 (0%) | 1373 (12%) | 1671 (15%) | 1922 (17%) | 2433 (21%) | 3133 (28%) | 3576 (32%) | 4137 (36%) |
|  | SF-36 Bodily pain | 17 (0%) | 1332 (12%) | 1634 (14%) | 1889 (17%) | 2399 (21%) | 3099 (27%) | 3573 (32%) | 4105 (36%) |
|  | SF-36 Vitality | 31 (0%) | 1373 (12%) | 1659 (15%) | 1915 (17%) | 2407 (21%) | 3114 (27%) | 3577 (32%) | 4116 (36%) |
|  | SF-36 General health | 76 (1%) | 1592 (14%) | 1843 (16%) | 2084 (18%) | 2404 (21%) | 3268 (29%) | 3698 (33%) | 4221 (37%) |
|  | SF-36 Role emotional | 42 (0%) | 1372 (12%) | 1689 (15%) | 1936 (17%) | 2436 (21%) | 3134 (28%) | 3616 (32%) | 4148 (37%) |
|  | SF-36 Social functioning | 16 (0%) | 1329 (12%) | 1630 (14%) | 1888 (17%) | 2398 (21%) | 3096 (27%) | 3573 (32%) | 4101 (36%) |
|  | SF-36 Mental health | 31 (0%) | 1368 (12%) | 1663 (15%) | 1917 (17%) | 2405 (21%) | 3108 (27%) | 3579 (32%) | 4114 (36%) |
| Exposure | Physical activity | - | 1759 (16%) | 2245 (20%) | 2440 (22%) | 2961 (26%) | 3383 (30%) | 3861 (34%) | - |
| Time-varying confounders | Vegetable intake^a^ | 11336 (100%) | 11336 (100%) | 1663 (15%) | 1938 (17%) | 2431 (21%) | 11336 (100%) | - | - |
|  | Fruit intake^a^ | 11336 (100%) | 11336 (100%) | 1649 (15%) | 1926 (17%) | 2414 (21%) | 11336 (100%) | - | - |
|  | Alcohol - frequency^b^ | 979 (9%) | 11336 (100%) | 1950 (17%) | 2177 (19%) | 2656 (23%) | 3407 (30%) | - | - |
|  | Alcohol - binge drinking^b^ | 697 (6%) | 11336 (100%) | 1778 (16%) | 1951 (17%) | 2434 (21%) | 3192 (28%) | - | - |
|  | BMI | 1345 (12%) | 1961 (17%) | 2184 (19%) | 2123 (19%) | 2603 (23%) | 3372 (30%) | - | - |
|  | Employment status | 132 (1%) | 1953 (17%) | 1900 (17%) | 2091 (18%) | 2616 (23%) | 3219 (28%) | - | - |
|  | CES-D | 812 (7%) | 1724 (15%) | 1836 (16%) | 2273 (20%) | 2632 (23%) | 3183 (28%) | - | - |
|  | SEIFA | 67 (1%) | 1383 (12%) | 1752 (15%) | 1982 (17%) | 2465 (22%) | 3134 (28%) | - | - |
|  | Marital status | 69 (1%) | 1362 (12%) | 1764 (16%) | 1954 (17%) | 2448 (22%) | 3163 (28%) | - | - |
|  | Smoking status | 681 (6%) | 1366 (12%) | 1653 (15%) | 1922 (17%) | 2411 (21%) | 3154 (28%) | - | - |
|  | Live with children - under 18 | 969 (9%) | 1371 (12%) | 1653 (15%) | 1909 (17%) | 2419 (21%) | 3153 (28%) | - | - |
|  | Live with children - over 18 | 931 (8%) | 1367 (12%) | 1653 (15%) | 1908 (17%) | 2419 (21%) | 3151 (28%) | - | - |
|  | Mean stress | 683 (6%) | 1357 (12%) | 1680 (15%) | 1917 (17%) | 2419 (21%) | 3135 (28%) | - | - |
|  | ARIA | 60 (1%) | 1377 (12%) | 1637 (14%) | 1905 (17%) | 2402 (21%) | 3116 (27%) | - | - |
|  | Age | 0 (0%) | 1314 (12%) | 1626 (14%) | 1882 (17%) | 2386 (21%) | 3091 (27%) | - | - |
|  | 3-year diagnosis/treatment for heart disease | 11336 (100%) | 1422 (13%) | 1890 (17%) | 1999 (18%) | 2481 (22%) | 3127 (28%) | - | - |
|  | 3-year diagnosis/treatment for stroke | 11336 (100%) | 1422 (13%) | 1890 (17%) | 1999 (18%) | 2481 (22%) | 3127 (28%) | - | - |
|  | 3-year diagnosis/treatment for cancer | 11336 (100%) | 1422 (13%) | 1890 (17%) | 1999 (18%) | 2481 (22%) | 3157 (28%) | - | - |
|  | 3-year diagnosis/treatment for arthritis | 11336 (100%) | 1422 (13%) | 1890 (17%) | 1999 (18%) | 2481 (22%) | 3151 (28%) | - | - |
|  | 3-year diagnosis/treatment for depression | 11336 (100%) | 1422 (13%) | 1890 (17%) | 1999 (18%) | 2481 (22%) | 3131 (28%) | - | - |
|  | 3-year diagnosis/treatment for anxiety | 11336 (100%) | 1422 (13%) | 1890 (17%) | 1999 (18%) | 2481 (22%) | 3131 (28%) | - | - |
| Baseline/time-constant confounders | Baseline - ever diagnosis/treatment for heart disease | 0 (0%) | - | - | - | - | - | - | - |
|  | Baseline - ever diagnosis/treatment for stroke | 0 (0%) | - | - | - | - | - | - | - |
|  | Baseline - ever diagnosis/treatment for cancer | 0 (0%) | - | - | - | - | - | - | - |
|  | Baseline - ever diagnosis/treatment for depression | 0 (0%) | - | - | - | - | - | - | - |
|  | Baseline - ever diagnosis/treatment for anxiety | 0 (0%) | - | - | - | - | - | - | - |
|  | Baseline - country of birth | 121 (1%) | - | - | - | - | - | - | - |
|  | Baseline - education | 94 (1%) | - | - | - | - | - | - | - |

Note: outcomes occurred in wave 9, but outcome variables were also used as time-varying confounders of exposure. Exposure was drawn from Waves 3-8; time-varying confounders were drawn from Waves 2-7; baseline/time-constant confounders were drawn from waves 1 and 2. ^a^ This was not asked in Waves 3 and 7; ^b^ This was not asked in Wave 3.

Fig A Most common patterns of missing data.


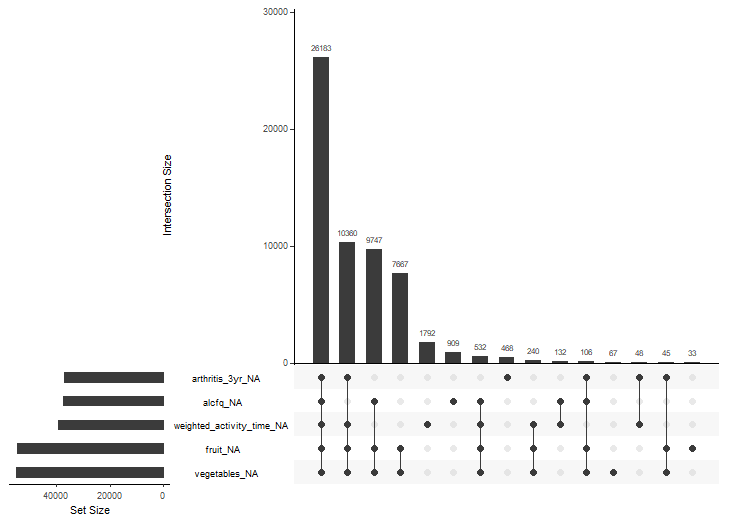


Dots indicate the variable(s) that is/are missing. The lines joining the dots together represent the set of variables that are missing in the given pattern.

References

1. Hughes RA, Heron J, Sterne JAC, Tilling K. Accounting for missing data in statistical analyses: multiple imputation is not always the answer. International Journal of Epidemiology. 2019;48(4):1294-304.

2. Huque MH, Carlin JB, Simpson JA, Lee KJ. A comparison of multiple imputation methods for missing data in longitudinal studies. BMC Medical Research Methodology. 2018;18(1):168.

3. van Buuren S, Groothuis-Oudshoorn K. mice: multivariate imputation by chained equations in R. Journal of Statistical Software. 2011;45(3).

4. Wright MN, Wager S, Probst P. Ranger: A fast implementation of random forests. R package version 012. 2020;1.

5. Graham JW, Olchowski AE, Gilreath TD. How many imputations are really needed? Some practical clarifications of multiple imputation theory. Prevention Science. 2007;8(3):206-13.

6. Honaker J, King G, Blackwell M. Amelia II: a program for missing data. Journal of Statistical Software. 2011;45(7):47.
