# Supplementary material for: Physical activity across midlife and health-related quality of life in Australian women: A target trial emulation using a longitudinal cohort
Source: PLoS Med. 2024 May 2;21(5):e1004384. doi: 10.1371/journal.pmed.1004384 (PMC11065283; doi:10.1371/journal.pmed.1004384)
Supplement: S4 Text — (DOCX) [file pmed.1004384.s005.docx]

# S4 Text

# E-value analysis

Regarding continuous outcomes, the function uses the effect-size conversions in Chinn (2000) and VanderWeele (2017) to approximately convert the mean difference between the exposed versus unexposed groups to the odds ratio that would arise from dichotomizing the continuous outcome.

For example, if resulting E-value is 2, this means that unmeasured confounder(s) would need to double the probability of a subject's being exposed versus not being exposed, and would also need to double the probability of being high versus low on the outcome, in which the cutoff for "high" versus "low" is arbitrary subject to some distributional assumptions (Chinn, 2000).

References

1. Chinn S. A simple method for converting an odds ratio to effect size for use in meta-analysis. Statistics in Medicine. 2000;19(22):3127-3131.

2. VanderWeele TJ. On a square-root transformation of the odds ratio for a common outcome. Epidemiology 2017;28(6):e58.
