# Supplementary material for: Physical activity across midlife and health-related quality of life in Australian women: A target trial emulation using a longitudinal cohort
Source: PLoS Med. 2024 May 2;21(5):e1004384. doi: 10.1371/journal.pmed.1004384 (PMC11065283; doi:10.1371/journal.pmed.1004384)
Supplement: S5 Text — (DOCX) [file pmed.1004384.s006.docx]

**S5 Text**

**Descriptive statistics of the unweighted analytical sample**

**Table A** Descriptive statistics of the unweighted analytical sample of Australian women at baseline.

| **Variable** | **Categories** | **Mean (SD) /**  **n (%) (N=11,336)** |
| --- | --- | --- |
| **Age** |  | 49.5 (1.5) |
| **Highest level of education** | High school or less | 7301 (64.4%) |
|  | Trade/apprentice/certificate/  diploma | 2258 (19.9%) |
|  | University | 1683 (14.8%) |
| **Country of birth** | Australia | 8638 (76.2%) |
|  | Other | 2577 (22.7%) |
| **Employment status** | Not employed | 2228 (19.7%) |
|  | Employed | 8976 (79.2%) |
| **Marital status** | Married/de facto | 9391 (82.8%) |
|  | Separated/divorced/never married | 1624 (14.3%) |
|  | Widowed | 252 (2.2%) |
| **Live with children under 18 years** | No | 7021 (61.9%) |
|  | Yes | 3346 (29.5%) |
| **Live with children aged 18+ years** | No | 6777 (59.8%) |
|  | Yes | 3628 (32.0%) |
| **Area-level SES (IRSD)** | Score for lowest tertile | 941 (25) |
|  | Score for middle tertile | 985 (11) |
|  | Score for highest tertile | 1062 (42) |
| **Remoteness (ARIA+)** | Major city | 3723 (32.8%) |
|  | Regional | 6986 (61.6%) |
|  | Remote | 567 (5.0%) |
| **Lifetime risky drinking^a^** | No | 8946 (78.9%) |
|  | Yes | 1411 (12.4%) |
| **Heavy episodic drinking^b^** | No | 7106 (62.7%) |
|  | Yes | 3533 (31.2%) |
| **Smoking status** | Never smoker | 6036 (53.2%) |
|  | Ex smoker | 2873 (25.3%) |
|  | Current smoker | 1746 (15.4%) |
| **CESD-10 Depression Score** |  | 6.2 (5.4) |
| **Mean Stress Score** |  | 0.6 (0.5) |
| **BMI Category** | Underweight | 138 (1.2%) |
|  | Healthy | 4779 (42.2%) |
|  | Overweight | 3139 (27.7%) |
|  | Obese | 1935 (17.1%) |
| **Ever diagnosed/treated for heart** | No | 11110 (98.0%) |
| **disease** | Yes | 226 (2.0%) |
| **Ever diagnosed/treated for stroke** | No | 11255 (99.3%) |
|  | Yes | 81 (0.7%) |
| **Ever diagnosed/treated for cancer** | No | 10903 (96.2%) |
|  | Yes | 433 (3.8%) |
| **Ever diagnosed/treated for depression** | No | 9477 (83.6%) |
|  | Yes | 1859 (16.4%) |
| **Ever diagnosed/treated for anxiety** | No | 9849 (86.9%) |
|  | Yes | 1487 (13.1%) |
| **SF-36 scores** | Physical Component Score | 51.08 (7.26) |
|  | Mental Component Score | 47.89 (11.68) |
|  | Physical functioning | 89.50 (11.24) |
|  | Role physical | 84.36 (31.05) |
|  | Bodily pain | 73.40 (21.70) |
|  | General health | 74.77 (18.14) |
|  | Vitality | 60.55 (19.82) |
|  | Social functioning | 84.68 (21.19) |
|  | Role emotional | 80.35 (33.71) |
|  | Mental health | 74.39 (16.89) |

Abbreviations: ARIA+, Accessibility-Remoteness Index of Australia Plus; BMI, body mass index; CESD-10, 10-item Centre for Epidemiological Studies Depression Scale; IRSD, Index of Relative Socio-Economic Disadvantage; SD, standard deviation; SF-36: 36-item Medical Outcomes Study short-form survey.

Note: percentages calculated as percent of total sample; percentages do not add to 100% due to missing data within variables.

^a^ Lifetime risky alcohol consumption defined as >10 alcoholic drinks/week based on the 2020 National Health Medical Research Council guidelines [34].

^b^ Heavy episodic alcohol consumption defined as >4 alcoholic drinks on an occasion at least once a month [34].
