# Supplementary material for: Physical activity across midlife and health-related quality of life in Australian women: A target trial emulation using a longitudinal cohort
Source: PLoS Med. 2024 May 2;21(5):e1004384. doi: 10.1371/journal.pmed.1004384 (PMC11065283; doi:10.1371/journal.pmed.1004384)
Supplement: S6 Text — (DOCX) [file pmed.1004384.s007.docx]

**S6 Text**

**Table A** Primary analysis of the effect of age at which started/stopped meeting physical activity guidelines on SF-36 component scores.

| **Counterfactual** | | **Expected Mean (99.5% CI)** | |
| --- | --- | --- | --- |
|  |  | **Physical Component Score** | **Mental Component Score** |
| Age started meeting guidelines (years) | Met guidelines in all waves | 46.93^a^  (46.32, 47.54) | 52.84  (52.27, 53.42) |
|  | 55 | 46.96^b^  (45.53, 48.40) | 52.92  (51.66, 54.17) |
|  | 60 | 45.12  (43.55, 46.70) | 52.18  (50.71, 53.65) |
|  | 65 | 44.14  (42.91, 45.37) | 52.30  (51.12, 53.47) |
|  | Did not meet guidelines in any wave | 43.90  (42.79, 45.01) | 52.02  (50.99, 53.06) |
| Age stopped meeting guidelines (years) | Did not meet guidelines in any wave | 43.90  (42.79, 45.01) | 52.02  (50.99, 53.06) |
|  | 55 | 43.88  (41.49, 46.26) | 51.82  (49.67, 53.97) |
|  | 60 | 45.13  (43.52, 46.74) | 52.10  (50.54, 53.67) |
|  | 65 | 46.46^c^  (45.69, 47.23) | 52.54  (51.76, 53.31) |
|  | Met guidelines in all waves | 46.93^a^  (46.32, 47.54) | 52.84  (52.27, 53.42) |

Abbreviations: CI, confidence interval; SF-36, 36-item Medical Outcomes Study short-form survey.

^a^ Significantly different from the reference group (did not meet guidelines in any wave).

^b^ Significantly different from the reference group (did not meet guidelines in any wave).

^c^ Significantly different from the reference group (did not meet guidelines in any wave).

**Table B** Secondary analysis of the effect of age at which started/stopped meeting physical activity guidelines on SF-36 subscales.

| **Counterfactual** | | **Expected Mean (99.5% CI)** | | | | | | | |
| --- | --- | --- | --- | --- | --- | --- | --- | --- | --- |
|  |  | **General Health** | **Physical functioning** | **Role physical** | **Bodily pain** | **Vitality** | **Social functioning** | **Role emotional** | **Mental health** |
| Age started meeting guidelines (years) | Met guidelines in all waves | 73.39^a^  (72.27, 74.51) | 80.60^a^  (79.29, 81.91) | 76.05^a^  (73.58, 78.52) | 69.00^a^  (67.59, 70.41) | 66.22^a^  (65.01, 67.42) | 86.73^a^  (85.33, 88.13) | 87.32  (85.24, 89.41) | 79.93^a^  (79.00, 80.86) |
|  | 55 | 72.47^b^  (69.38, 75.56) | 79.91 ^b^  (77.17, 82.65) | 77.61^b^  (71.22, 84.00) | 69.21  (65.44, 72.99) | 65.62^b^  (63.24, 68.00) | 87.68^b^  (84.34, 91.01) | 89.41^b^  (84.95, 93.87) | 79.37  (76.95, 81.78) |
|  | 60 | 70.66  (68.00, 73.32) | 73.93  (70.12, 77.73) | 74.21  (67.91, 80.51) | 66.59  (63.36, 69.81) | 63.48  (60.82, 66.13) | 84.25  (80.89, 87.62) | 84.27  (79.07, 89.47) | 78.91  (76.66, 81.17) |
|  | 65 | 69.07  (66.74, 71.39) | 72.98  (70.33, 75.63) | 70.06  (64.85, 75.28) | 64.39  (61.50, 67.28) | 61.53  (59.11, 63.94) | 84.10  (81.47, 86.72) | 85.91  (82.45, 89.36) | 78.18  (76.27, 80.08) |
|  | Did not meet guidelines in any wave | 68.39  (66.20, 70.59) | 71.23  (68.94, 73.52) | 70.52  (65.77, 75.28) | 64.26  (61.60, 66.92) | 60.96  (58.83, 63.09) | 82.86  (80.36, 85.36) | 83.99  (80.50, 87.48) | 78.00  (76.40, 79.60) |
| Age stopped meeting guidelines (years) | Did not meet guidelines in any wave | 68.39  (66.20, 70.59) | 71.23  (68.94, 73.52) | 70.52  (65.77, 75.28) | 64.26  (61.60, 66.92) | 60.96  (58.83, 63.09) | 82.86  (80.36, 85.36) | 83.99  (80.50, 87.48) | 78.00  (76.40, 79.60) |
|  | 55 | 68.96  (65.65, 72.27) | 73.87  (68.32, 79.42) | 67.11  (58.43, 75.79) | 63.69  (59.01, 68.36) | 60.91  (56.74, 65.07) | 82.75  (78.15, 87.36) | 83.43  (76.17, 90.69) | 78.12  (75.09, 81.15) |
|  | 60 | 70.29  (67.62, 72.95) | 76.06^c^  (72.59, 79.54) | 70.98  (65.74, 76.23) | 65.68  (62.66, 68.71) | 63.37  (60.79, 65.95) | 84.01  (80.79, 87.22) | 83.68  (79.07, 88.29) | 78.73  (75.99, 81.47) |
|  | 65 | 72.63^d^  (70.99, 74.27) | 79.79^d^  (78.21, 81.37) | 74.09  (71.09, 77.10) | 67.95^d^  (66.02, 69.89) | 65.32^d^  (63.76, 66.88) | 86.15^d^  (84.48, 87.82) | 86.00  (83.41, 88.59) | 79.41  (78.20, 80.62) |
|  | Met guidelines in all waves | 73.39^a^  (72.27, 74.51) | 80.60^a^  (79.29, 81.91) | 76.05^a^  (73.58, 78.52) | 69.00^a^  (67.59, 70.41) | 66.22^a^  (65.01, 67.42) | 86.73^a^  (85.33, 88.13) | 87.32  (85.24, 89.41) | 79.93^a^  (79.00, 80.86) |

Abbreviation: CI, confidence interval; SF-36, 36-item Medical Outcomes Study short-form survey.

^a^ Significantly different from reference group (did not meet guidelines in any wave).

^b^ Significantly different from reference group (did not meet guidelines in any wave).

^c^ Significantly different from reference group (did not meet guidelines in any wave).

^d^ Significantly different from reference group (did not meet guidelines in any wave).

**Fig A** Secondary analysis of the effect of age at which started to meet physical activity guidelines on SF-36 subscales.


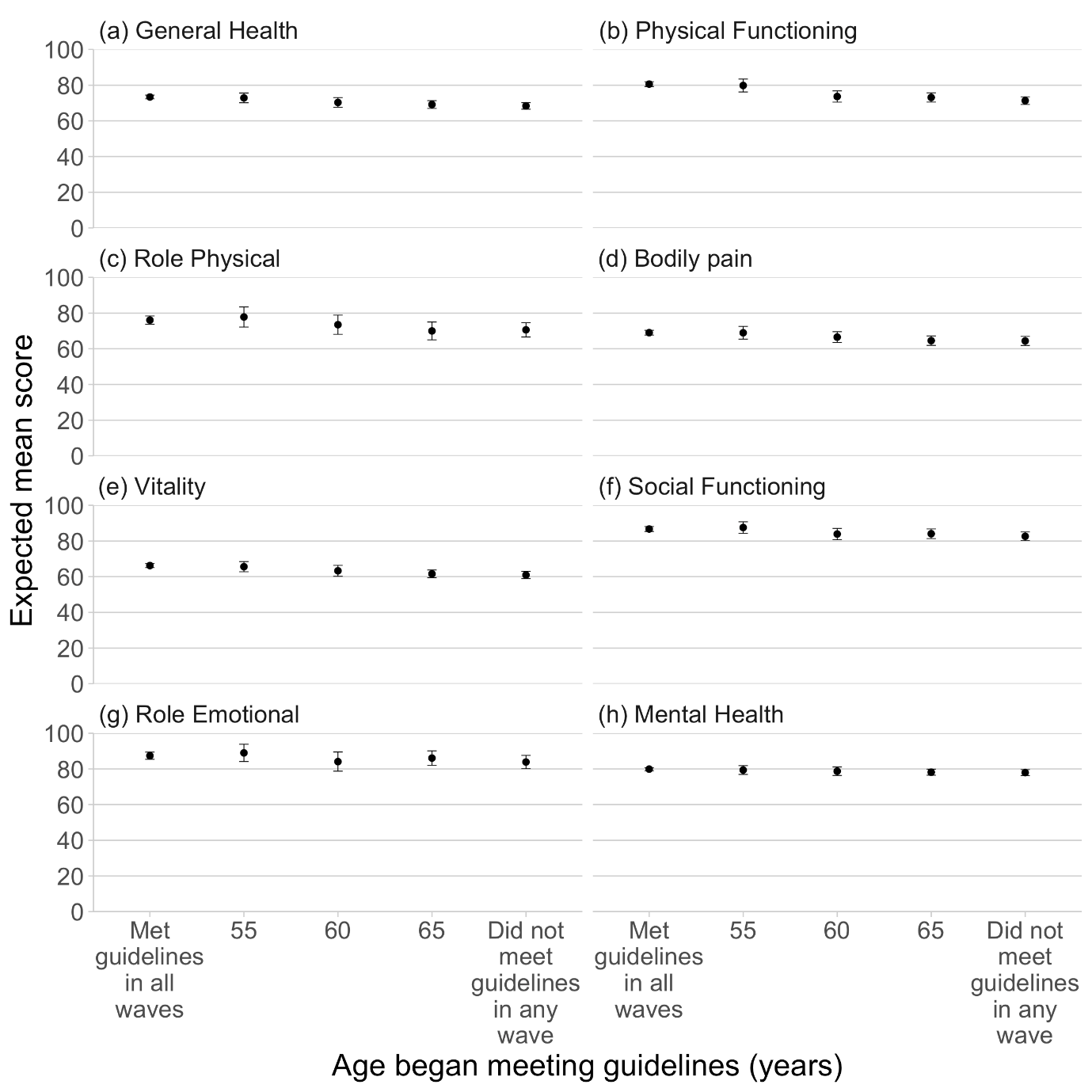
 Abbreviations: SF-36, 36-item Medical Outcomes Study short-form survey.

This figure shows the effect of meeting physical activity upon reaching a particular age (‘starters’) but not prior to that, with a range of age thresholds considered (55, 60, and 65 years), on SF-36 subscale scores at survey 9. The points represent the estimates and the bars the 99.5% confidence intervals.

Models were adjusted for: highest level of education, country of birth, age, employment status, living with children, marital status, Socio-Economic Index For Areas Index of Relative Socio-Economic Disadvantage (SEIFA IRSD), geographical remoteness (Accessibility-Remoteness Index of Australia Plus, ARIA+), history of coronary heart disease diagnosis/treatment, history of stroke diagnosis/treatment, history of arthritis diagnosis/treatment, history of any cancer diagnosis/treatment, history of anxiety diagnosis/treatment, and history of depression diagnosis/treatment, Center for Epidemiological Studies-Depression (CES-D) scale, stress, SF-36 subscale scores, body mass index, lifetime risky alcohol consumption based on the 2020 National Health Medical Research Council guidelines, heavy episodic alcohol consumption, smoking status, vegetable intake, and fruit intake.

**Fig B** Primary analysis of the effect of age at which ceased to meet physical activity guidelines on SF-36 component scores.


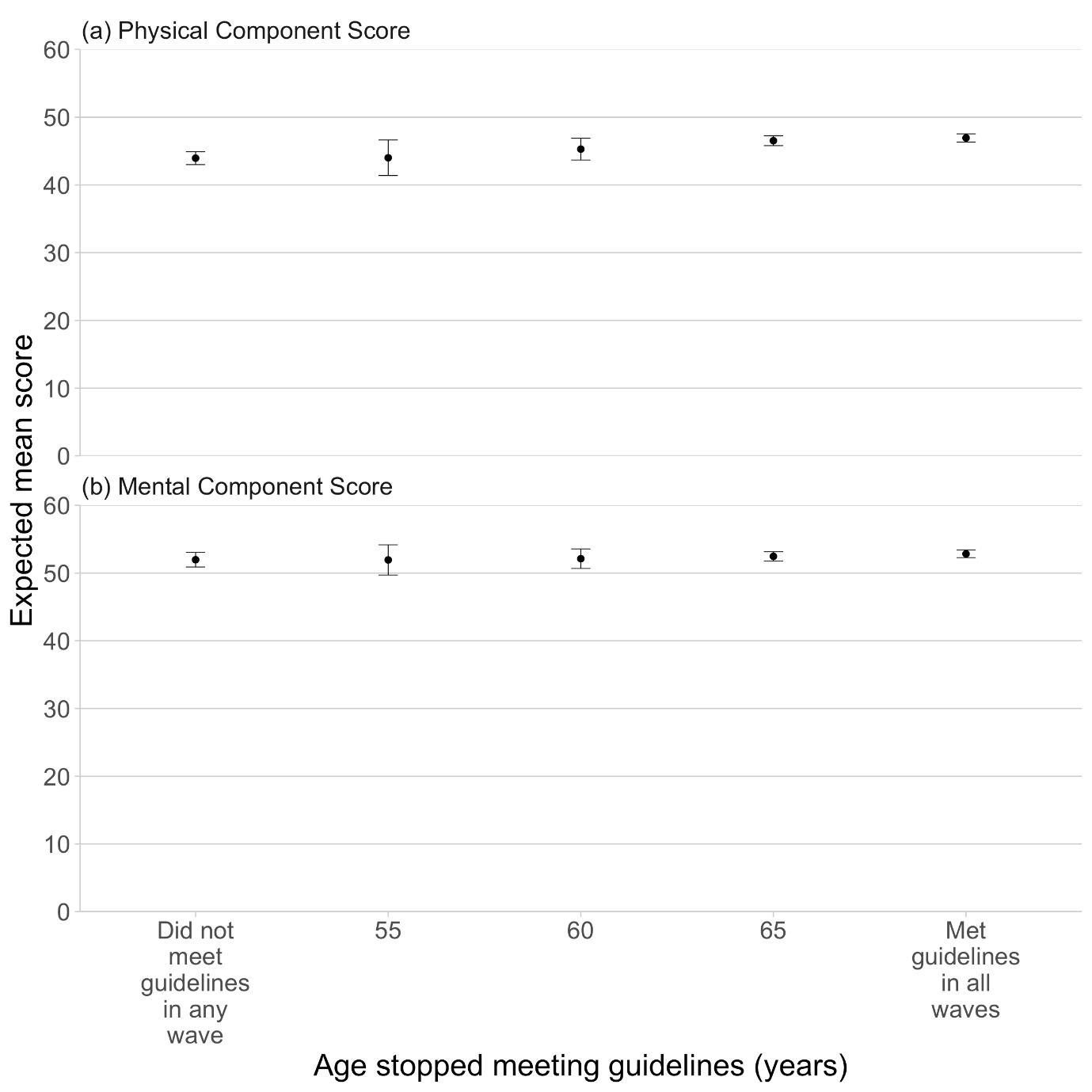
 Abbreviations: SF-36, 36-item Medical Outcomes Study short-form survey.

This figure shows the effect of meeting physical activity guidelines up to a particular age (55, 60, or 65 years), and then ceasing to meet guidelines thereafter (‘non-sustainers’), on the physical health component and mental health component scores at survey 9.

Models were adjusted for: highest level of education, country of birth, age, employment status, living with children, marital status, Socio-Economic Index For Areas Index of Relative Socio-Economic Disadvantage (SEIFA IRSD), geographical remoteness (Accessibility-Remoteness Index of Australia Plus, ARIA+), history of coronary heart disease diagnosis/treatment, history of stroke diagnosis/treatment, history of arthritis diagnosis/treatment, history of any cancer diagnosis/treatment, history of anxiety diagnosis/treatment, and history of depression diagnosis/treatment, Center for Epidemiological Studies-Depression (CES-D) scale, stress, SF-36 subscale scores, body mass index, lifetime risky alcohol consumption based on the 2020 National Health Medical Research Council guidelines, heavy episodic alcohol consumption, smoking status, vegetable intake, and fruit intake.

**Fig C** Secondary analysis of the effect of age at which ceased to meet physical activity guidelines on SF-36 subscales.**
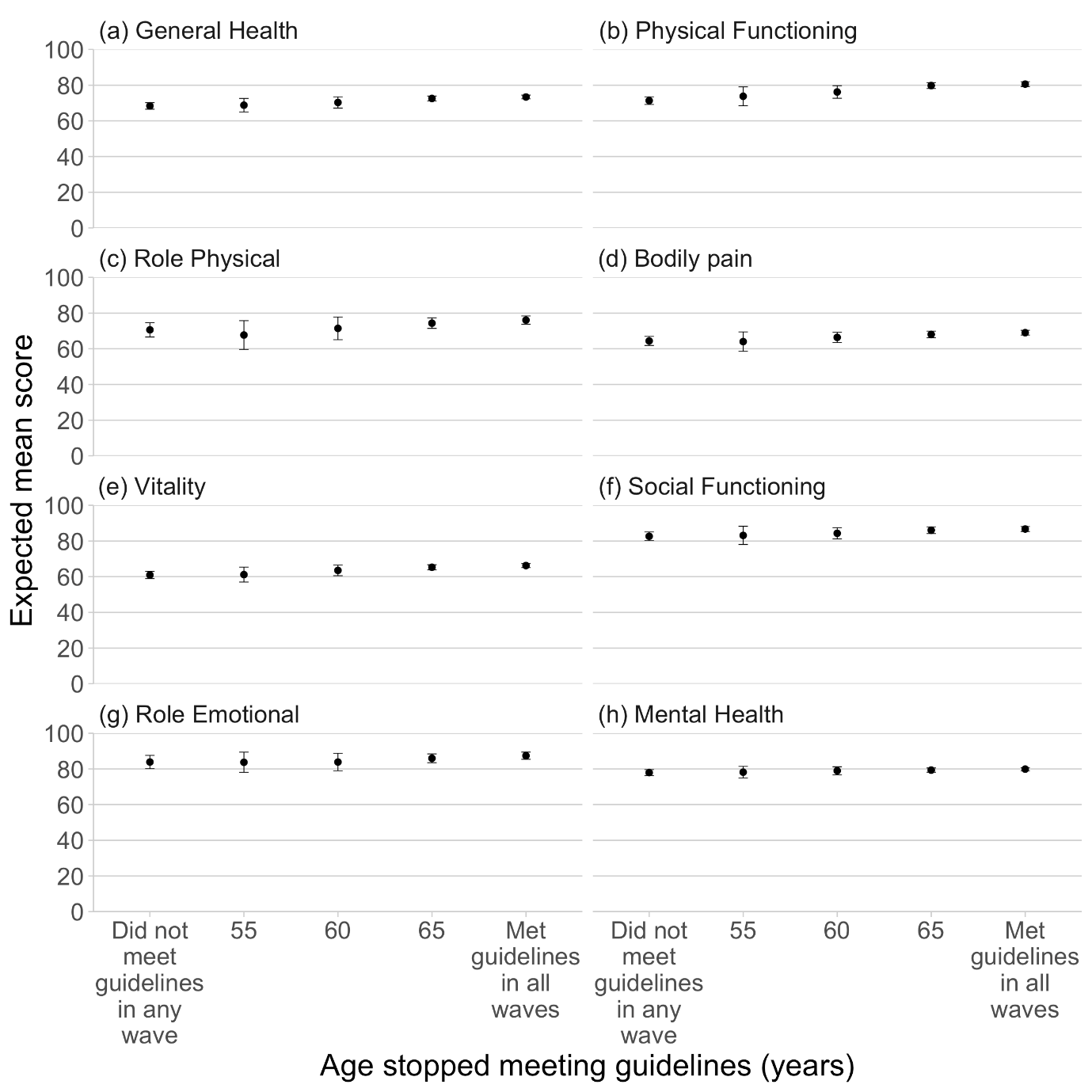
**

This figure shows the effect of meeting physical activity guidelines up to a particular age (55, 60, or 65 years), and then ceasing to meet guidelines thereafter (‘non-sustainers’), on SF-36 subscale scores at survey 9. The points represent the estimates and the bars the 99.5% confidence intervals.

Models were adjusted for: highest level of education, country of birth, age, employment status, living with children, marital status, Socio-Economic Index For Areas Index of Relative Socio-Economic Disadvantage (SEIFA IRSD), geographical remoteness (Accessibility-Remoteness Index of Australia Plus, ARIA+), history of coronary heart disease diagnosis/treatment, history of stroke diagnosis/treatment, history of arthritis diagnosis/treatment, history of any cancer diagnosis/treatment, history of anxiety diagnosis/treatment, and history of depression diagnosis/treatment, Center for Epidemiological Studies-Depression (CES-D) scale, stress, SF-36 subscale scores, body mass index, lifetime risky alcohol consumption based on the 2020 National Health Medical Research Council guidelines, heavy episodic alcohol consumption, smoking status, vegetable intake, and fruit intake.

**Table C** Analysis of the effect of age at which started/ceased to meet physical activity guidelines on SF-36 component scores– sensitivity analysis using lower threshold of 75 minutes per week for physical activity.

| **Counterfactual** | | **Expected Mean (99.5% CI)** | |
| --- | --- | --- | --- |
|  |  | **Physical Component Score** | **Mental Component Score** |
| Age started meeting guidelines (years) | Met guidelines in all waves | 46.72^a^  (46.25, 47.19) | 52.78^a^  (52.33, 53.23) |
|  | 55 | 46.01  (44.32, 47.69) | 52.58^b^  (51.36, 53.80) |
|  | 60 | 45.07  (42.99, 47.15) | 52.81^c^  (50.84, 54.78) |
|  | 65 | 43.65  (42.10, 45.20) | 50.96  (49.29, 52.64) |
|  | Did not meet guidelines in any wave | 44.33  (42.61, 46.05) | 50.34  (48.69, 51.99) |
| Age stopped meeting guidelines (years) | Did not meet guidelines in any wave | 44.33  (42.61, 46.05) | 50.34  (48.69, 51.99) |
|  | 55 | 43.43  (41.19, 45.66) | 52.80^b^  (50.79, 54.81) |
|  | 60 | 45.09  (43.55, 46.63) | 52.32^c^  (50.40, 54.24) |
|  | 65 | 46.14^d^  (45.58, 46.71) | 52.41^d^  (51.85, 52.97) |
|  | Met guidelines in all waves | 46.72^a^  (46.25, 47.19) | 52.78^a^  (52.33, 53.23) |

Abbreviation: CI, confidence interval; SF-36, 36-item Medical Outcomes Study short-form survey.

^a^ Significantly different from the reference group (did not meet guidelines in any wave).

^b^ Significantly different from the reference group (did not meet guidelines in any wave).

^c^ Significantly different from the reference group (did not meet guidelines in any wave).

^d^ Significantly different from the reference group (did not meet guidelines in any wave).

**Fig D** Analysis of the effect of age at which started to meet physical activity guidelines on SF-36 component scores – sensitivity analysis using lower threshold of 75 minutes per week for physical activity.**
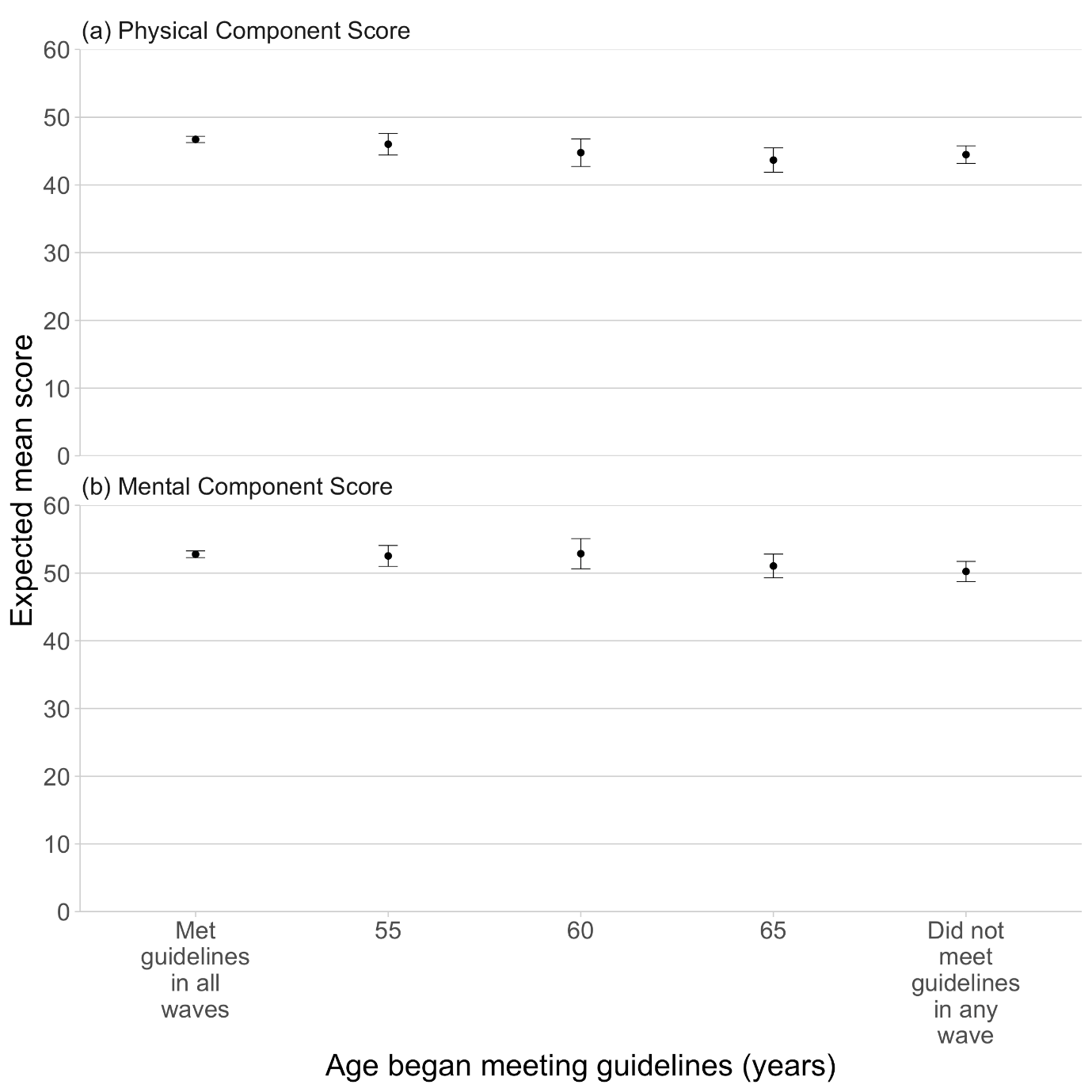
**

This figure shows the effect of meeting physical activity upon reaching a particular age (‘starters’) but not prior to that, with a range of age thresholds considered (55, 60, and 65 years), on the physical health component and mental health component scores at survey 9. The points represent the estimates and the bars the 99.5% confidence intervals.

Models were adjusted for: highest level of education, country of birth, age, employment status, living with children, marital status, Socio-Economic Index For Areas Index of Relative Socio-Economic Disadvantage (SEIFA IRSD), geographical remoteness (Accessibility-Remoteness Index of Australia Plus, ARIA+), history of coronary heart disease diagnosis/treatment, history of stroke diagnosis/treatment, history of arthritis diagnosis/treatment, history of any cancer diagnosis/treatment, history of anxiety diagnosis/treatment, and history of depression diagnosis/treatment, Center for Epidemiological Studies-Depression (CES-D) scale, stress, SF-36 subscale scores, body mass index, lifetime risky alcohol consumption based on the 2020 National Health Medical Research Council guidelines, heavy episodic alcohol consumption, smoking status, vegetable intake, and fruit intake.

**Fig E** Analysis of the effect of age at which ceased to meet physical activity guidelines on SF-36 component scores – sensitivity analysis using lower threshold of 75 minutes per week for physical activity.**
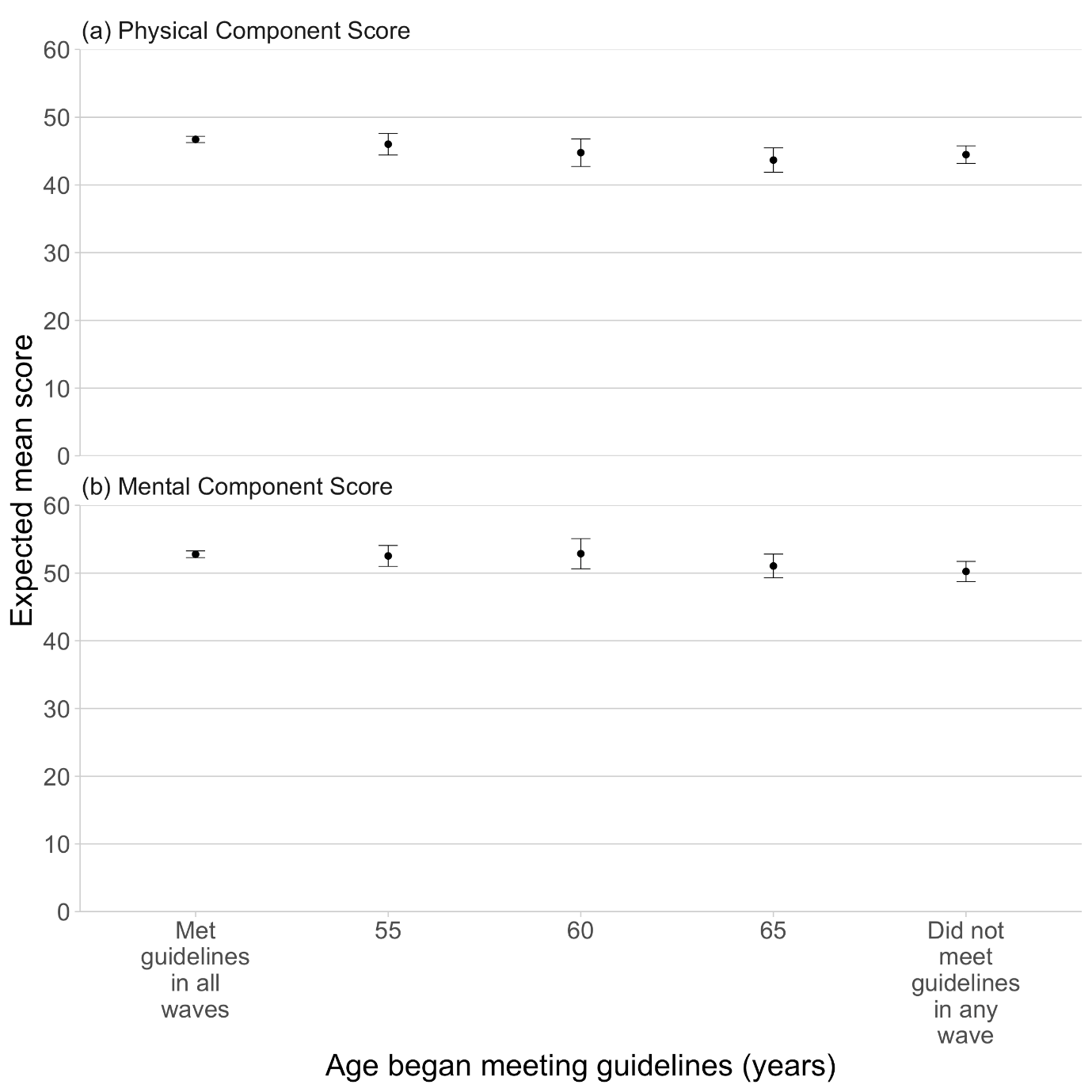
**

This figure shows the effect of meeting physical activity guidelines up to a particular age (55, 60, or 65 years), and then ceasing to meet guidelines thereafter (‘non-sustainers’), on the physical health component and mental health component scores at survey 9. The points represent the estimates and the bars the 99.5% confidence intervals.

Models were adjusted for: highest level of education, country of birth, age, employment status, living with children, marital status, Socio-Economic Index For Areas Index of Relative Socio-Economic Disadvantage (SEIFA IRSD), geographical remoteness (Accessibility-Remoteness Index of Australia Plus, ARIA+), history of coronary heart disease diagnosis/treatment, history of stroke diagnosis/treatment, history of arthritis diagnosis/treatment, history of any cancer diagnosis/treatment, history of anxiety diagnosis/treatment, and history of depression diagnosis/treatment, Center for Epidemiological Studies-Depression (CES-D) scale, stress, SF-36 subscale scores, body mass index, lifetime risky alcohol consumption based on the 2020 National Health Medical Research Council guidelines, heavy episodic alcohol consumption, smoking status, vegetable intake, and fruit intake.

**Table D** Secondary analysis of the effect of age at which started/ceased to meet physical activity guidelines on SF-36 subscales – sensitivity analyses using lower threshold of 75 minutes per week for physical activity.

| **Counterfactual** | | **Expected Mean (99.5% CI)** | | | | | | | |
| --- | --- | --- | --- | --- | --- | --- | --- | --- | --- |
|  |  | **General Health** | **Physical functioning** | **Role physical** | **Bodily pain** | **Vitality** | **Social functioning** | **Role emotional** | **Mental health** |
| Age started meeting guidelines (years) | Met guidelines in all waves | 72.99^a^  (72.10, 73.89) | 80.01^a^  (79.03, 80.98) | 75.48^a^  (73.58, 77.38) | 68.75  (67.59, 69.91) | 65.88^a^  (64.96, 66.80) | 86.29^a^  (85.24, 87.35) | 87.49^a^  (85.94, 89.04) | 79.66^a^  (78.92, 80.41) |
|  | 55 | 70.94^b^  (67.84, 74.05) | 77.89^b^  (75.05, 80.73) | 74.25  (67.48, 81.02) | 67.89  (64.36, 71.41) | 63.73^b^  (60.60, 66.87) | 87.04^b^  (83.73, 90.35) | 87.60^b^  (83.37, 91.82) | 78.92^b^  (76.65, 81.20) |
|  | 60 | 71.29^c^  (67.39, 75.18) | 73.40  (68.09, 78.71) | 75.68  (67.31, 84.06) | 67.31  (63.18, 71.44) | 63.66^c^  (59.38, 67.93) | 85.89  (80.98, 90.80) | 86.47^c^  (79.38, 93.57) | 79.46^c^  (76.34, 82.57) |
|  | 65 | 66.75  (63.33, 70.16) | 70.14  (66.59, 73.69) | 67.59  (61.03, 74.14) | 65.15  (61.40, 68.90) | 59.16  (55.58, 62.74) | 81.49  (77.93, 85.06) | 82.64  (77.25, 88.03) | 75.63  (73.08, 78.19) |
|  | Did not meet guidelines in any wave | 66.99  (63.38, 70.60) | 70.68  (67.44, 73.92) | 67.78  (60.90, 74.66) | 66.28  (62.28, 70.29) | 58.46  (55.62, 61.30) | 81.79  (78.10, 85.49) | 78.56  (73.44, 83.68) | 75.45  (73.16, 77.74) |
| Age stopped meeting guidelines (years) | Did not meet guidelines in any wave | 66.99  (63.38, 70.60) | 70.68  (67.44, 73.92) | 67.78  (60.90, 74.66) | 66.28  (62.28, 70.29) | 58.46  (55.62, 61.30) | 81.79  (78.10, 85.49) | 78.56  (73.44, 83.68) | 75.45  (73.16, 77.74) |
|  | 55 | 69.87  (66.09, 73.66) | 72.07  (67.12, 77.03) | 67.60  (58.22, 76.98) | 63.65  (59.04, 68.25) | 62.04  (58.14, 65.94) | 83.10  (79.01, 87.19) | 84.09  (77.12, 91.07) | 79.76^b^  (76.39, 83.13) |
|  | 60 | 69.77  (67.15, 72.40) | 75.41^c^  (72.47, 78.34) | 73.38  (68.50, 78.26) | 65.83  (62.77, 68.90) | 63.34^c^  (61.06, 65.61) | 84.41  (80.99, 87.84) | 85.34^c^  (80.60, 90.09) | 78.76^c^  (76.12, 81.40) |
|  | 65 | 71.96^d^  (70.76, 73.17) | 78.76^d^  (77.54, 79.98) | 73.46  (71.10, 75.83) | 67.93  (66.33, 69.53) | 64.87^d^  (63.75, 65.99) | 85.28  (83.93, 86.62) | 85.81^d^  (83.84, 87.78) | 79.26^d^  (78.34, 80.17) |
|  | Met guidelines in all waves | 72.99^a^  (72.10, 73.89) | 80.01^a^  (79.03, 80.98) | 75.48^a^  (73.58, 77.38) | 68.75  (67.59, 69.91) | 65.88^a^  (64.96, 66.80) | 86.29^a^  (85.24, 87.35) | 87.49^a^  (85.94, 89.04) | 79.66^a^  (78.92, 80.41) |

Abbreviation: CI, confidence interval; SF-36, 36-item Medical Outcomes Study short-form survey.

^a^ Significantly different from reference group (did not meet guidelines in any wave).

^b^ Significantly different from reference group (did not meet guidelines in any wave).

^c^ Significantly different from reference group (did not meet guidelines in any wave).

^d^ Significantly different from reference group (did not meet guidelines in any wave).

**Fig F** Analysis of the effect of age at which started to meet physical activity guidelines on SF-36 subscales – sensitivity analysis using lower threshold of 75 minutes per week for physical activity.**
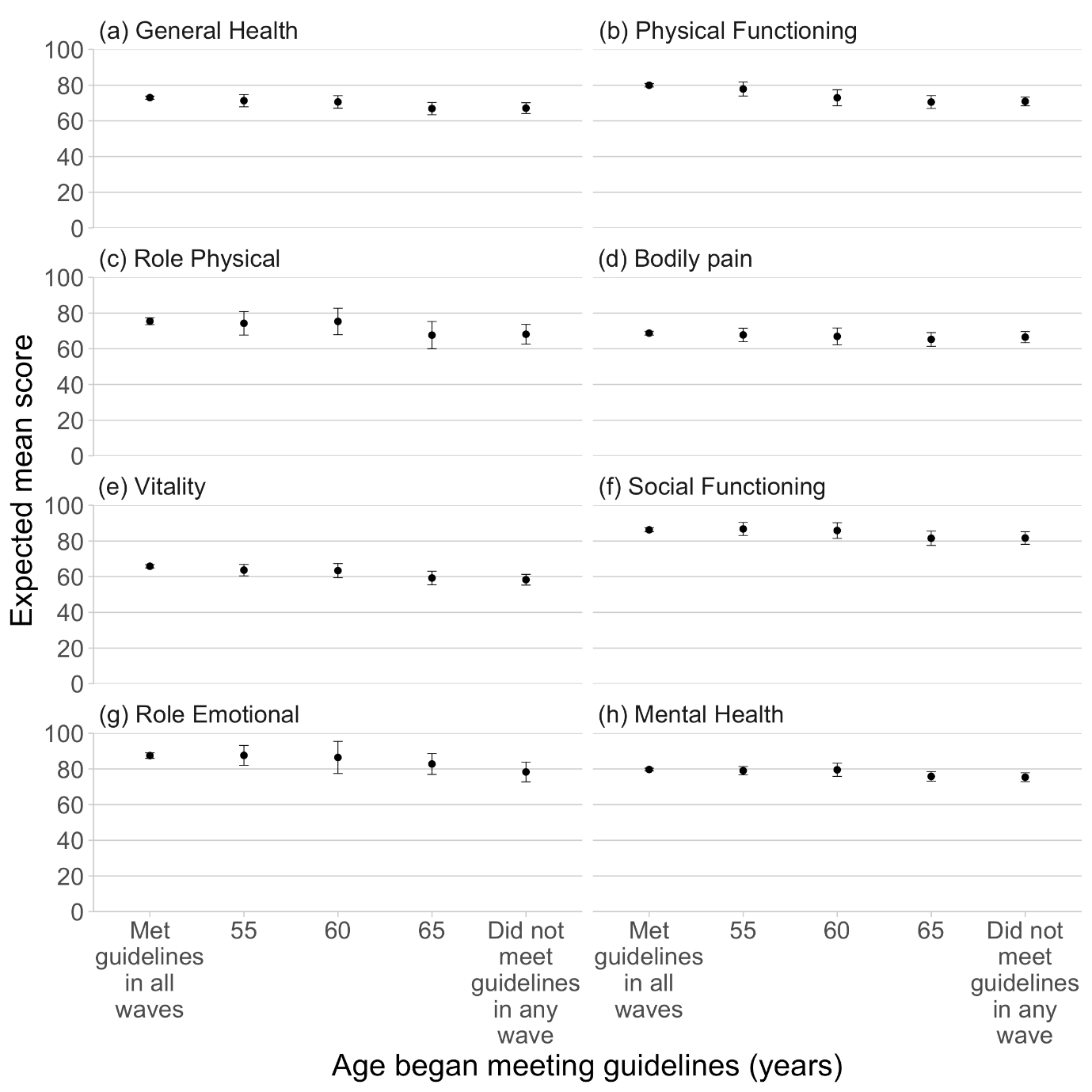
**

This figure shows the effect of meeting physical activity upon reaching a particular age (‘starters’) but not prior to that, with a range of age thresholds considered (55, 60, and 65 years), on SF-36 subscale scores at survey 9. The points represent the estimates and the bars the 99.5% confidence intervals.

Models were adjusted for: highest level of education, country of birth, age, employment status, living with children, marital status, Socio-Economic Index For Areas Index of Relative Socio-Economic Disadvantage (SEIFA IRSD), geographical remoteness (Accessibility-Remoteness Index of Australia Plus, ARIA+), history of coronary heart disease diagnosis/treatment, history of stroke diagnosis/treatment, history of arthritis diagnosis/treatment, history of any cancer diagnosis/treatment, history of anxiety diagnosis/treatment, and history of depression diagnosis/treatment, Center for Epidemiological Studies-Depression (CES-D) scale, stress, SF-36 subscale scores, body mass index, lifetime risky alcohol consumption based on the 2020 National Health Medical Research Council guidelines, heavy episodic alcohol consumption, smoking status, vegetable intake, and fruit intake.

**Fig G** Analysis of the effect of age at which ceased to meet physical activity guidelines on SF-36 subscales – sensitivity analysis using lower threshold of 75 minutes per week for physical activity.**
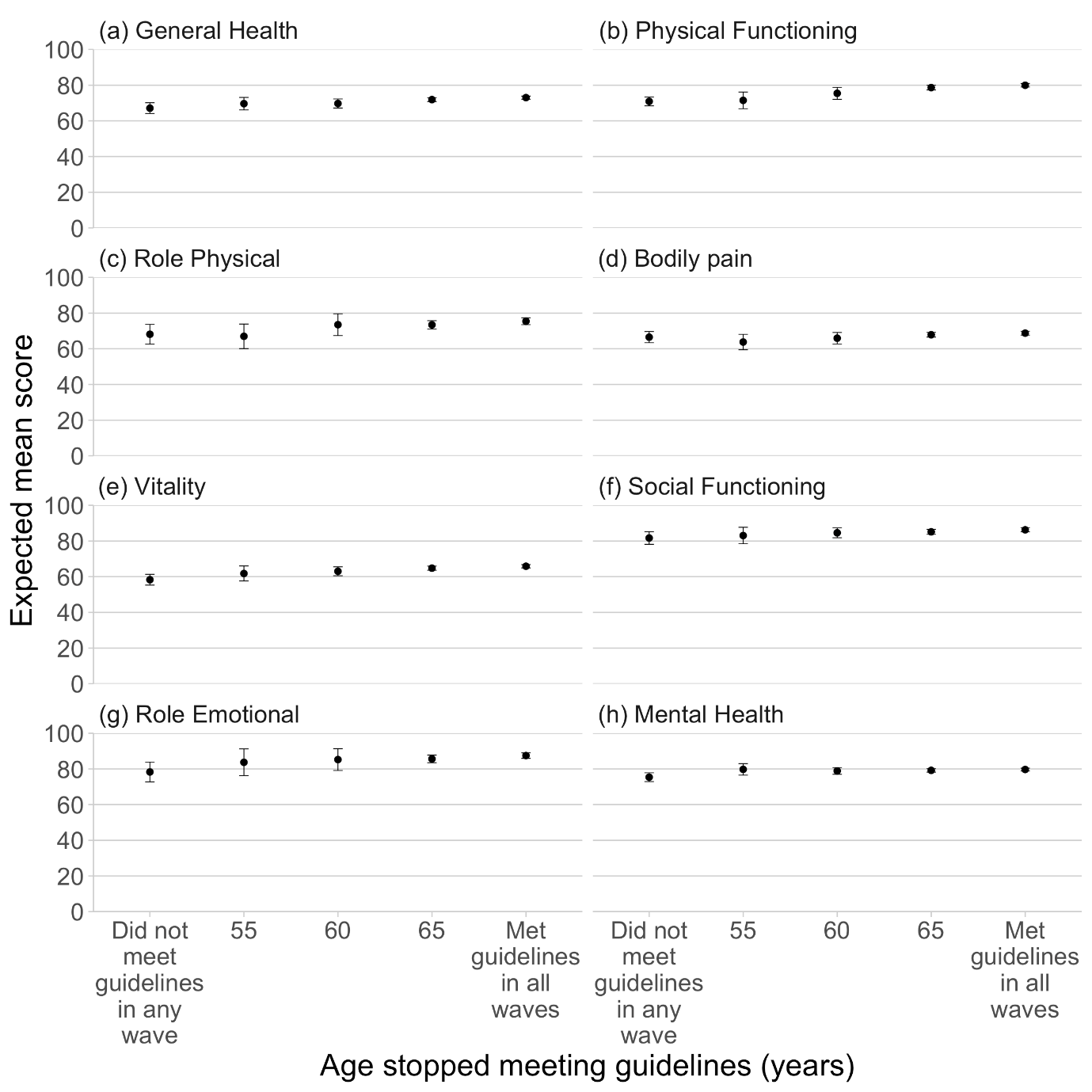
**

This figure shows the effect of meeting physical activity guidelines up to a particular age (55, 60, or 65 years), and then ceasing to meet guidelines thereafter (‘non-sustainers’), on SF-36 subscale scores at survey 9. The points represent the estimates and the bars the 99.5% confidence intervals.

Models were adjusted for: highest level of education, country of birth, age, employment status, living with children, marital status, Socio-Economic Index For Areas Index of Relative Socio-Economic Disadvantage (SEIFA IRSD), geographical remoteness (Accessibility-Remoteness Index of Australia Plus, ARIA+), history of coronary heart disease diagnosis/treatment, history of stroke diagnosis/treatment, history of arthritis diagnosis/treatment, history of any cancer diagnosis/treatment, history of anxiety diagnosis/treatment, and history of depression diagnosis/treatment, Center for Epidemiological Studies-Depression (CES-D) scale, stress, SF-36 subscale scores, body mass index, lifetime risky alcohol consumption based on the 2020 National Health Medical Research Council guidelines, heavy episodic alcohol consumption, smoking status, vegetable intake, and fruit intake.

**Table E** Analysis of the effect of age at which started/stopped meeting physical activity guidelines SF-36 component scores– sensitivity analysis using higher threshold of 300 minutes per week for physical activity.

| **Counterfactual** | | **Expected Mean (99.5% CI)** | |
| --- | --- | --- | --- |
|  |  | **Physical Component Score** | **Mental Component Score** |
| Age started meeting guidelines (years) | Met guidelines in all waves | 46.78^a^  (45.64, 47.92) | 52.94^a^  (52.05, 53.83) |
|  | 55 | 46.52^b^  (45.44, 47.59) | 53.01  (51.89, 54.14) |
|  | 60 | 46.14^c^  (45.06, 47.23) | 52.75  (51.69, 53.82) |
|  | 65 | 44.90  (44.09, 45.70) | 52.04  (51.21, 52.87) |
|  | Did not meet guidelines in any wave | 44.50  (43.83, 45.18) | 51.97  (51.30, 52.64) |
| Age stopped meeting guidelines (years) | Did not meet guidelines in any wave | 44.50  (43.83, 45.18) | 51.97  (51.30, 52.64) |
|  | 55 | 44.75  (42.77, 46.73) | 52.24  (50.29, 54.18) |
|  | 60 | 46.26  (44.26, 48.27) | 53.48  (51.59, 55.37) |
|  | 65 | 46.18^d^  (44.94, 47.42) | 53.32^d^  (52.14, 54.50) |
|  | Met guidelines in all waves | 46.78^a^  (45.64, 47.92) | 52.94^a^  (52.05, 53.83) |

Abbreviation: CI, confidence interval; SF-36, 36-item Medical Outcomes Study short-form survey.

^a^ Significantly different from the reference group (did not meet guidelines in any wave).

^b^ Significantly different from the reference group (did not meet guidelines in any wave).

^c^ Significantly different from the reference group (did not meet guidelines in any wave).

^d^ Significantly different from the reference group (did not meet guidelines in any wave).

**Fig H** Analysis of the effect of age at which started to meet physical activity guidelines on SF-36 component scores – sensitivity analysis using higher threshold of 300 minutes per week for physical activity.


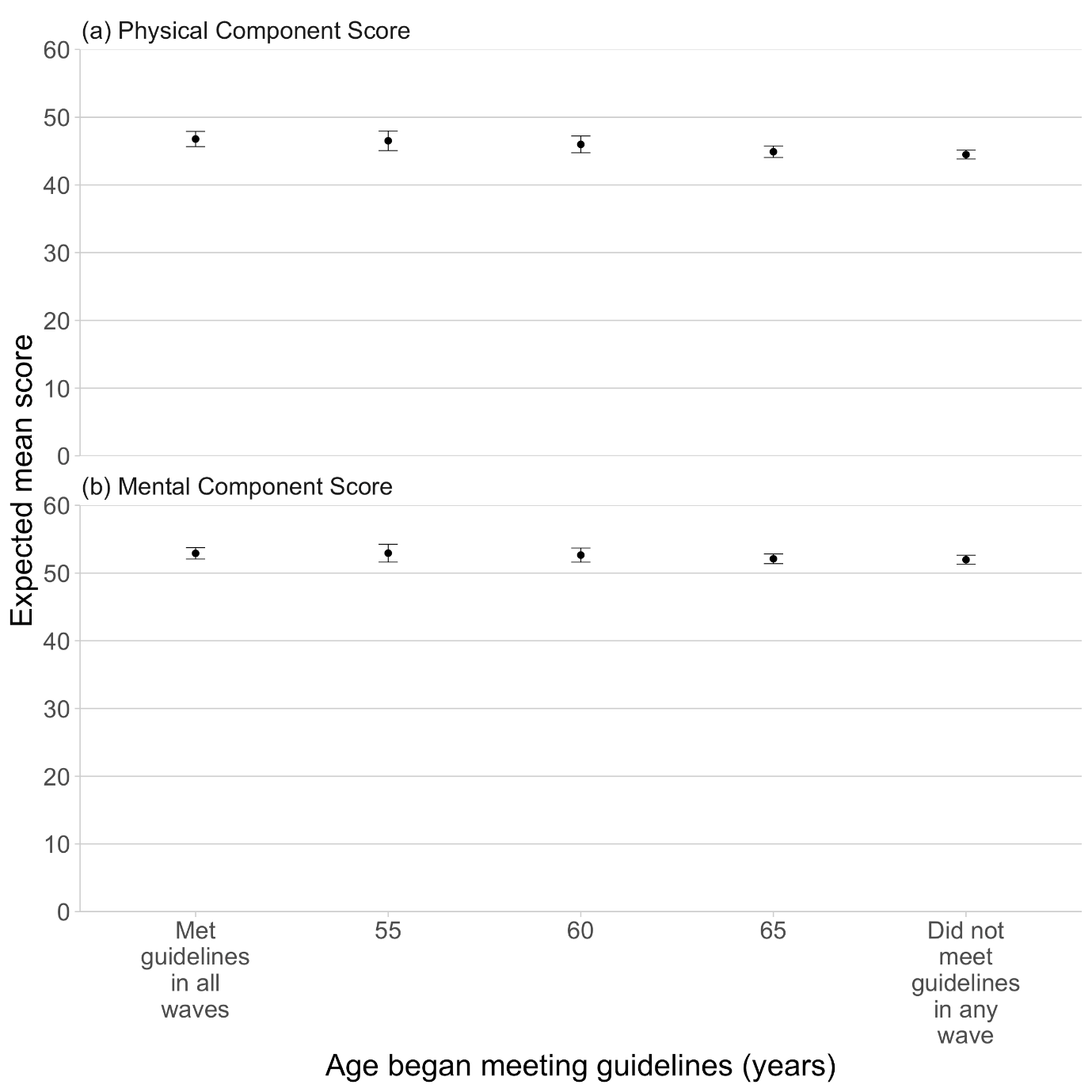


This figure shows the effect of meeting physical activity upon reaching a particular age (‘starters’) but not prior to that, with a range of age thresholds considered (55, 60, and 65 years), on the physical health component and mental health component scores at survey 9. The points represent the estimates and the bars the 99.5% confidence intervals.

Models were adjusted for: highest level of education, country of birth, age, employment status, living with children, marital status, Socio-Economic Index For Areas Index of Relative Socio-Economic Disadvantage (SEIFA IRSD), geographical remoteness (Accessibility-Remoteness Index of Australia Plus, ARIA+), history of coronary heart disease diagnosis/treatment, history of stroke diagnosis/treatment, history of arthritis diagnosis/treatment, history of any cancer diagnosis/treatment, history of anxiety diagnosis/treatment, and history of depression diagnosis/treatment, Center for Epidemiological Studies-Depression (CES-D) scale, stress, SF-36 subscale scores, body mass index, lifetime risky alcohol consumption based on the 2020 National Health Medical Research Council guidelines, heavy episodic alcohol consumption, smoking status, vegetable intake, and fruit intake.

**Fig I** Analysis of the effect of age at which ceased to meet physical activity guidelines on SF-36 component scores – sensitivity analysis using higher threshold of 300 minutes per week for physical activity.

**
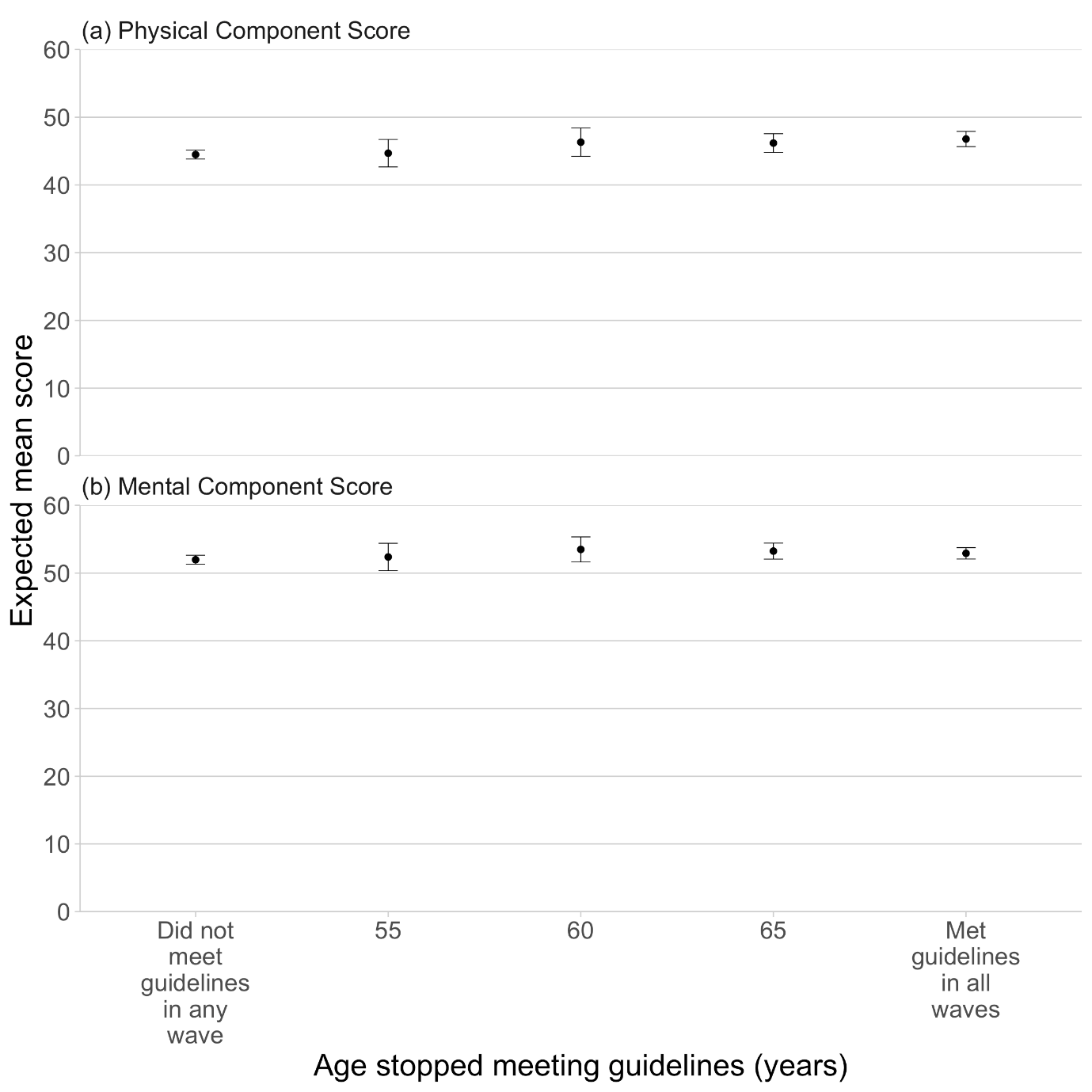
**

This figure shows the effect of meeting physical activity guidelines up to a particular age (55, 60, or 65 years), and then ceasing to meet guidelines thereafter (‘non-sustainers’), on the physical health component and mental health component scores at survey 9. The points represent the estimates and the bars the 99.5% confidence intervals.

Models were adjusted for: highest level of education, country of birth, age, employment status, living with children, marital status, Socio-Economic Index For Areas Index of Relative Socio-Economic Disadvantage (SEIFA IRSD), geographical remoteness (Accessibility-Remoteness Index of Australia Plus, ARIA+), history of coronary heart disease diagnosis/treatment, history of stroke diagnosis/treatment, history of arthritis diagnosis/treatment, history of any cancer diagnosis/treatment, history of anxiety diagnosis/treatment, and history of depression diagnosis/treatment, Center for Epidemiological Studies-Depression (CES-D) scale, stress, SF-36 subscale scores, body mass index, lifetime risky alcohol consumption based on the 2020 National Health Medical Research Council guidelines, heavy episodic alcohol consumption, smoking status, vegetable intake, and fruit intake.

**
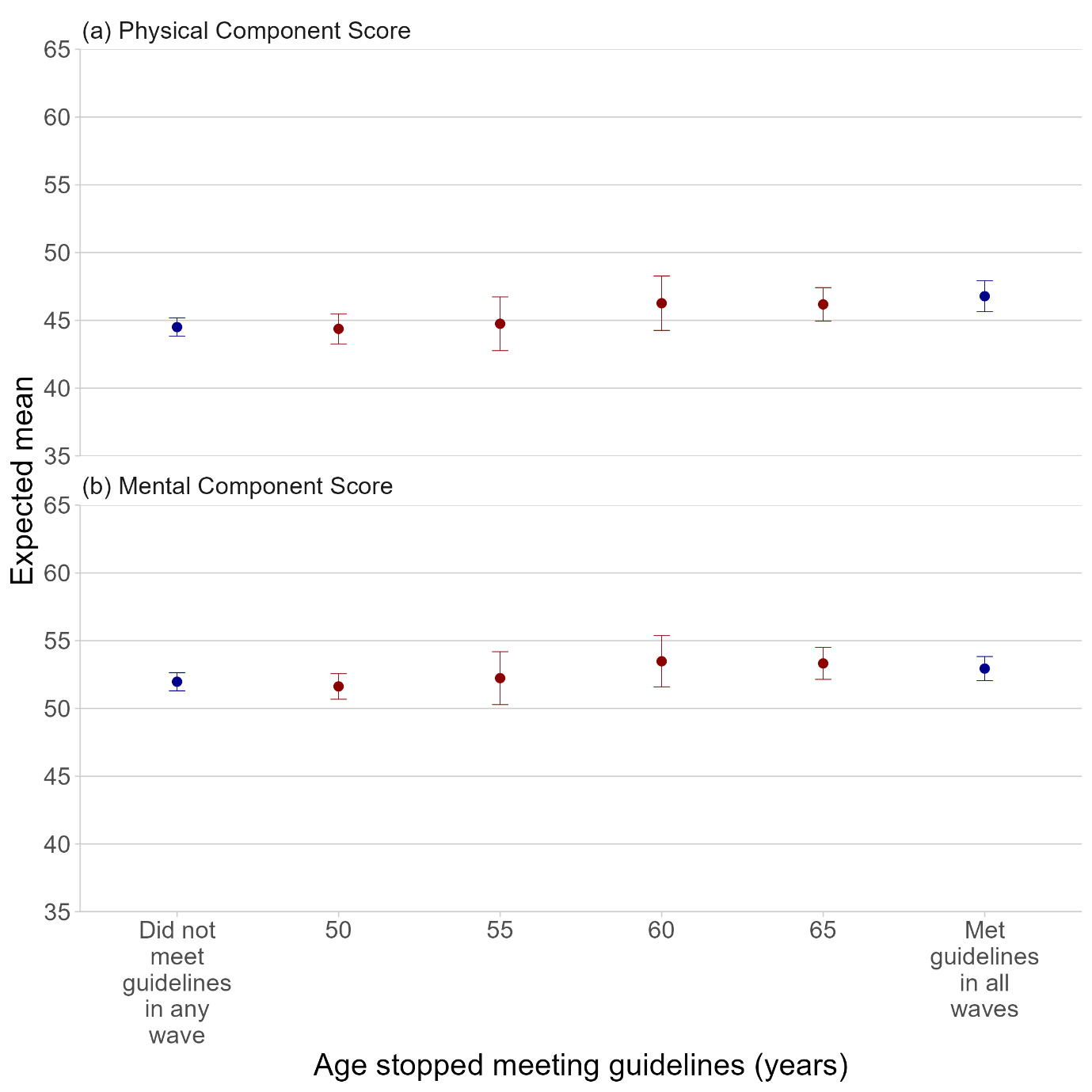
**

**Table F** Analysis of the effect of age at which started/ceased to meet physical activity guidelines SF-36 subscales – sensitivity analysis using higher threshold of 300 minutes per week for physical activity.

| **Counterfactual** | | **Expected Mean (99.5% CI)** | | | | | | | |
| --- | --- | --- | --- | --- | --- | --- | --- | --- | --- |
|  |  | **General Health** | **Physical functioning** | **Role physical** | **Bodily pain** | **Vitality** | **Social functioning** | **Role emotional** | **Mental health** |
| Age started meeting guidelines (years) | Met guidelines in all waves | 73.16^a^  (71.20, 75.12) | 81.04^a^  (78.80, 83.29) | 74.92^a^  (70.68, 79.15) | 68.55^a^  (66.10, 70.99) | 66.24^a^  (64.09, 68.40) | 85.99  (83.67, 88.32) | 89.39^a^  (86.48, 92.29) | 79.99^a^  (78.57, 81.41) |
|  | 55 | 73.83^b^  (71.15, 76.51) | 80.23^b^  (77.43, 83.04) | 75.22^b^  (71.26, 79.18) | 67.31  (64.68, 69.94) | 66.34^b^  (64.12, 68.57) | 85.79  (83.05, 88.52) | 88.64^b^  (84.99, 92.28) | 79.93  (77.93, 81.92) |
|  | 60 | 72.61^c^  (70.81, 74.42) | 77.57^c^  (75.13, 80.01) | 75.00  (69.78, 80.21) | 68.39  (65.39, 71.40) | 64.94^c^  (62.77, 67.11) | 85.79  (82.78, 88.80) | 87.33  (82.98, 91.69) | 79.34  (77.58, 81.10) |
|  | 65 | 69.86  (68.41, 71.32) | 74.48  (72.79, 76.17) | 70.81  (67.49, 74.13) | 66.24  (64.22, 68.25) | 62.32  (60.79, 63.86) | 84.38  (82.47, 86.29) | 84.67  (82.11, 87.23) | 78.13  (76.84, 79.43) |
|  | Did not meet guidelines in any wave | 69.05  (67.83, 70.28) | 72.98  (71.49, 74.46) | 70.32  (67.65, 72.98) | 65.98  (64.34, 67.61) | 61.51  (60.23, 62.79) | 84.02  (82.60, 85.43) | 83.94  (81.78, 86.10) | 77.99  (76.90, 79.08) |
| Age stopped meeting guidelines (years) | Did not meet guidelines in any wave | 69.05  (67.83, 70.28) | 72.98  (71.49, 74.46) | 70.32  (67.65, 72.98) | 65.98  (64.34, 67.61) | 61.51  (60.23, 62.79) | 84.02  (82.60, 85.43) | 83.94  (81.78, 86.10) | 77.99  (76.90, 79.08) |
|  | 55 | 68.49  (65.24, 71.74) | 76.82^b^  (73.14, 80.50) | 68.97  (61.36, 76.59) | 66.00  (61.11, 70.89) | 62.67  (59.18, 66.16) | 83.94  (79.30, 88.58) | 85.85  (80.11, 91.59) | 78.79  (75.93, 81.65) |
|  | 60 | 73.79^c^  (69.91, 77.66) | 78.57^c^  (74.30, 82.83) | 76.01  (67.61, 84.40) | 68.13  (63.87, 72.39) | 66.77^c^  (62.98, 70.55) | 87.29  (83.22, 91.37) | 87.19  (81.64, 92.74) | 80.56  (77.66, 83.46) |
|  | 65 | 72.87^d^  (70.43, 75.31) | 79.80^d^  (76.99, 82.62) | 75.02^d^  (70.59, 79.45) | 67.39  (64.65, 70.13) | 66.00^d^  (63.52, 68.49) | 86.83^d^  (84.19, 89.47) | 89.90^d^  (86.11, 93.69) | 80.27^d^  (78.38, 82.16) |
|  | Met guidelines in all waves | 73.16^a^  (71.20, 75.12) | 81.04^a^  (78.80, 83.29) | 74.92^a^  (70.68, 79.15) | 68.55^a^  (66.10, 70.99) | 66.24^a^  (64.09, 68.40) | 85.99  (83.67, 88.32) | 89.39^a^  (86.48, 92.29) | 79.99^a^  (78.57, 81.41) |

Abbreviation: CI, confidence interval; SF-36, 36-item Medical Outcomes Study short-form survey.

^a^ Significantly different from reference group (did not meet guidelines in any wave).

^b^ Significantly different from reference group (did not meet guidelines in any wave).

^c^ Significantly different from reference group (did not meet guidelines in any wave).

^d^ Significantly different from reference group (did not meet guidelines in any wave).

**Fig J** Analysis of the effect of age at which started to meet physical activity guidelines on SF-36 subscales – sensitivity analysis using higher threshold of 300 minutes per week for physical activity.


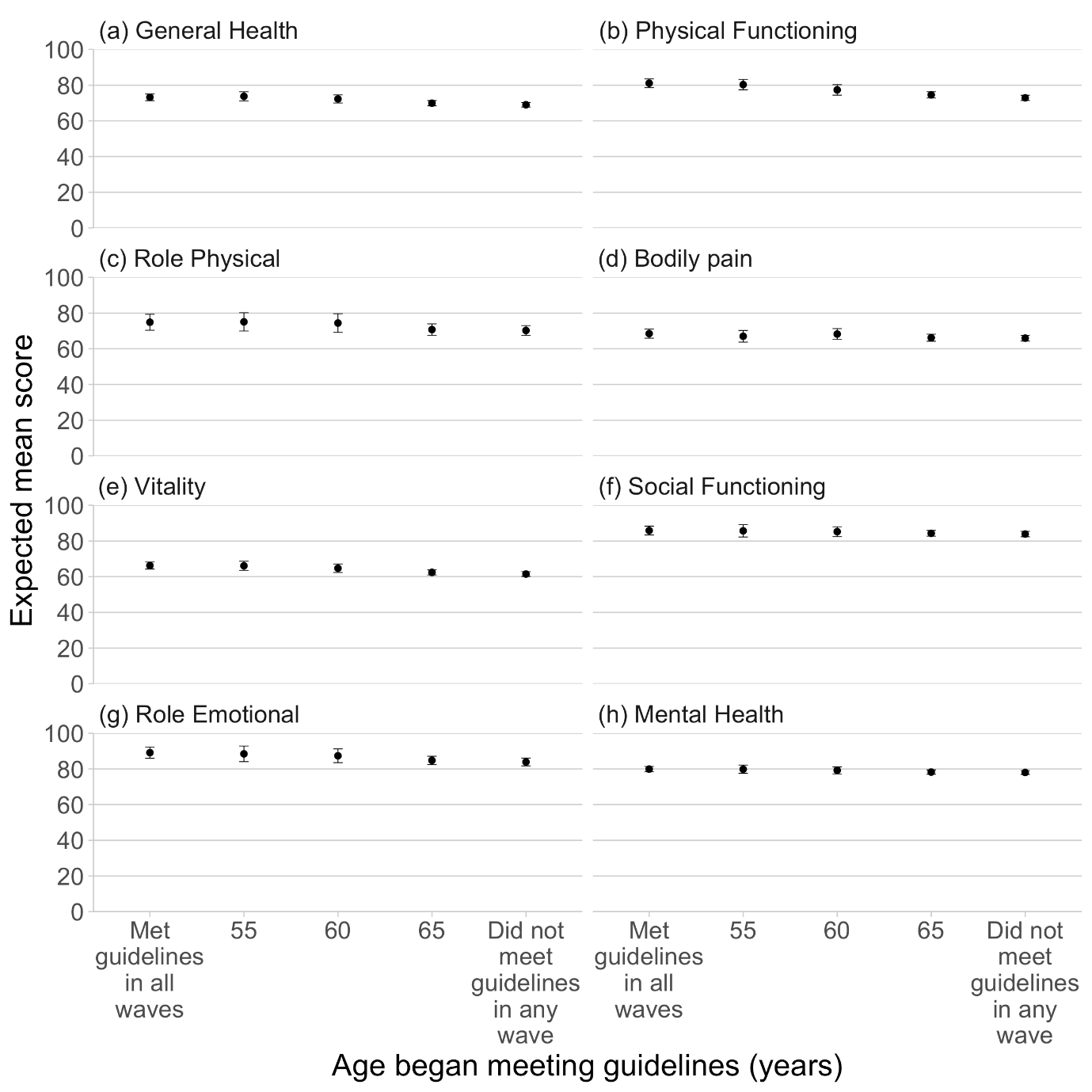


This figure shows the effect of meeting physical activity upon reaching a particular age (‘starters’) but not prior to that, with a range of age thresholds considered (55, 60, and 65 years), on SF-36 subscale scores at survey 9. The points represent the estimates and the bars the 99.5% confidence intervals.

Models were adjusted for: highest level of education, country of birth, age, employment status, living with children, marital status, Socio-Economic Index For Areas Index of Relative Socio-Economic Disadvantage (SEIFA IRSD), geographical remoteness (Accessibility-Remoteness Index of Australia Plus, ARIA+), history of coronary heart disease diagnosis/treatment, history of stroke diagnosis/treatment, history of arthritis diagnosis/treatment, history of any cancer diagnosis/treatment, history of anxiety diagnosis/treatment, and history of depression diagnosis/treatment, Center for Epidemiological Studies-Depression (CES-D) scale, stress, SF-36 subscale scores, body mass index, lifetime risky alcohol consumption based on the 2020 National Health Medical Research Council guidelines, heavy episodic alcohol consumption, smoking status, vegetable intake, and fruit intake.

**Fig K** Analysis of the effect of age at which ceased to meet physical activity guidelines on SF-36 subscales – sensitivity analysis using higher threshold of 300 minutes per week for physical activity.


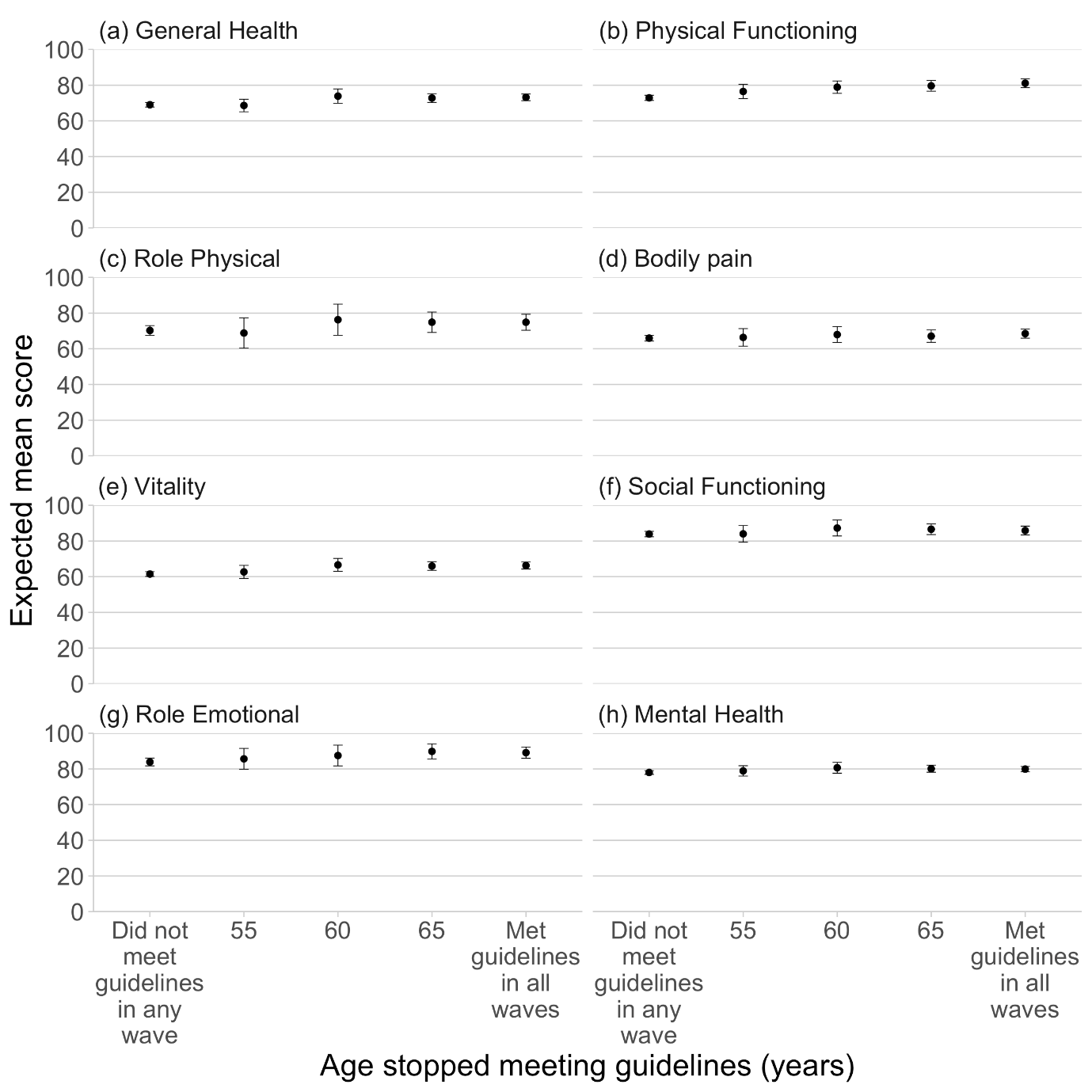


This figure shows the effect of meeting physical activity guidelines up to a particular age (55, 60, or 65 years), and then ceasing to meet guidelines thereafter (‘non-sustainers’), on the SF-36 subscale scores at survey 9. The points represent the estimates and the bars the 99.5% confidence intervals.

Models were adjusted for: highest level of education, country of birth, age, employment status, living with children, marital status, Socio-Economic Index For Areas Index of Relative Socio-Economic Disadvantage (SEIFA IRSD), geographical remoteness (Accessibility-Remoteness Index of Australia Plus, ARIA+), history of coronary heart disease diagnosis/treatment, history of stroke diagnosis/treatment, history of arthritis diagnosis/treatment, history of any cancer diagnosis/treatment, history of anxiety diagnosis/treatment, and history of depression diagnosis/treatment, Center for Epidemiological Studies-Depression (CES-D) scale, stress, SF-36 subscale scores, body mass index, lifetime risky alcohol consumption based on the 2020 National Health Medical Research Council guidelines, heavy episodic alcohol consumption, smoking status, vegetable intake, and fruit intake.

**Table G** Analysis of the effect of age at which started/stopped meeting physical activity guidelines SF-36 component scores– sensitivity analysis excluding covariates in waves where they were completely missing.

| **Counterfactual** | | **Expected Mean (99.5% CI)** | |
| --- | --- | --- | --- |
|  |  | **Physical Component Score** | **Mental Component Score** |
| Age started meeting guidelines (years) | Met guidelines in all waves | 46.93^a^  (46.32, 47.54) | 52.84  (52.25, 53.43) |
|  | 55 | 46.97^b^  (45.55, 48.38) | 52.92  (51.64, 54.19) |
|  | 60 | 45.13  (43.56, 46.69) | 52.19  (50.73, 53.64) |
|  | 65 | 44.15  (42.92, 45.37) | 52.30  (51.13, 53.46) |
|  | Did not meet guidelines in any wave | 43.91  (42.79, 45.02) | 52.03  (50.99, 53.06) |
| Age stopped meeting guidelines (years) | Did not meet guidelines in any wave | 43.91  (42.79, 45.02) | 52.03  (50.99, 53.06) |
|  | 55 | 43.88  (41.54, 46.22) | 51.82  (49.63, 54.01) |
|  | 60 | 45.13  (43.48, 46.79) | 52.09  (50.52, 53.66) |
|  | 65 | 46.45^d^  (45.68, 47.22) | 52.54  (51.74, 53.34) |
|  | Met guidelines in all waves | 46.93^a^  (46.32, 47.54) | 52.84  (52.25, 53.43) |

Abbreviation: CI, confidence interval; SF-36, 36-item Medical Outcomes Study short-form survey.

^a^ Significantly different from the reference group (did not meet guidelines in any wave).

^b^ Significantly different from the reference group (did not meet guidelines in any wave).

^c^ Significantly different from the reference group (did not meet guidelines in any wave).

^d^ Significantly different from the reference group (did not meet guidelines in any wave).

**Fig L** Analysis of the effect of age at which started to meet physical activity guidelines on SF-36 component scores – sensitivity analysis excluding covariates in waves where they were completely missing.


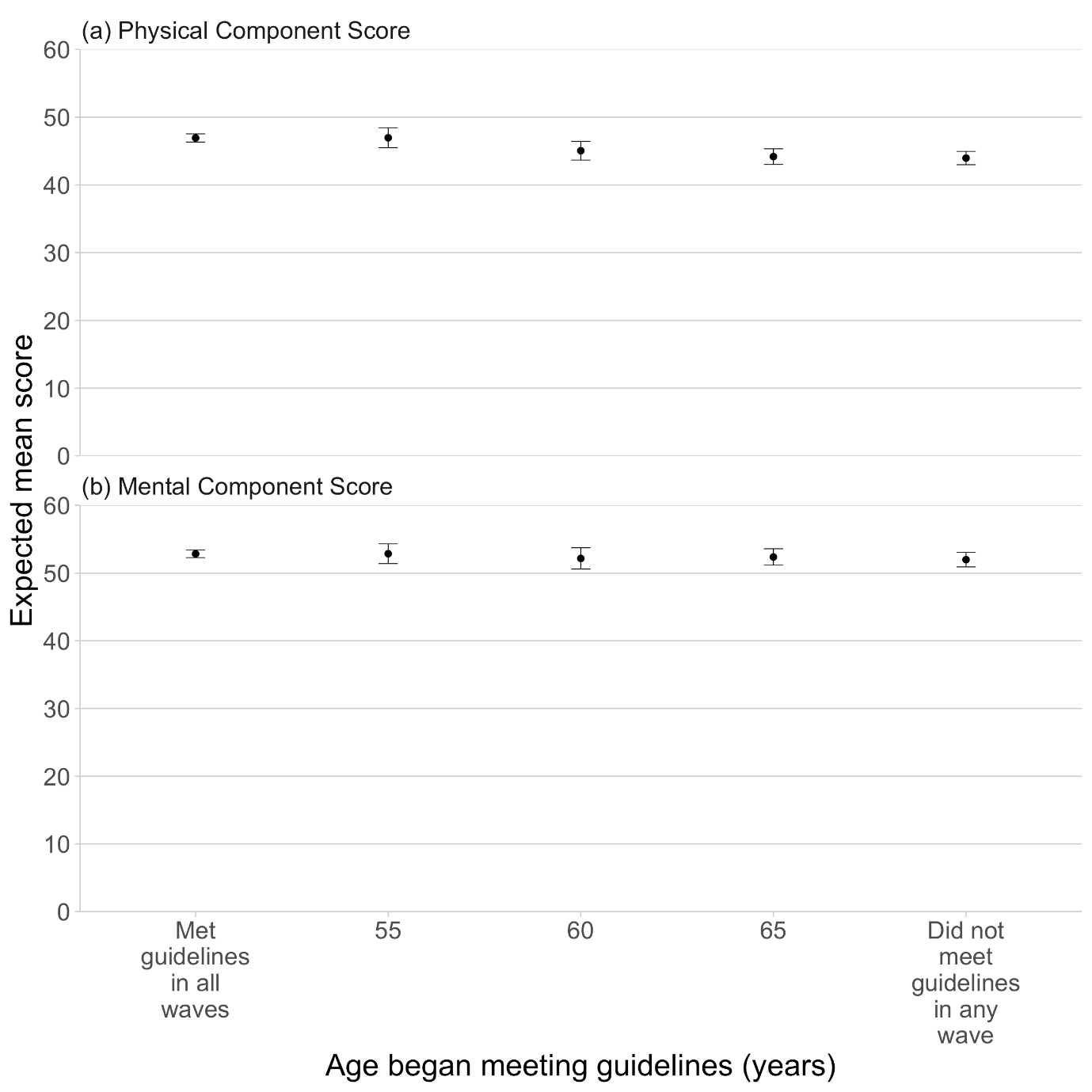


This figure shows the effect of meeting physical activity upon reaching a particular age (‘starters’) but not prior to that, with a range of age thresholds considered (55, 60, and 65 years), on the physical health component and mental health component scores at survey 9. The points represent the estimates and the bars the 99.5% confidence intervals.

Models were adjusted for: highest level of education, country of birth, age, employment status, living with children, marital status, Socio-Economic Index For Areas Index of Relative Socio-Economic Disadvantage (SEIFA IRSD), geographical remoteness (Accessibility-Remoteness Index of Australia Plus, ARIA+), history of coronary heart disease diagnosis/treatment, history of stroke diagnosis/treatment, history of arthritis diagnosis/treatment, history of any cancer diagnosis/treatment, history of anxiety diagnosis/treatment, and history of depression diagnosis/treatment, Center for Epidemiological Studies-Depression (CES-D) scale, stress, SF-36 subscale scores, body mass index, lifetime risky alcohol consumption based on the 2020 National Health Medical Research Council guidelines, heavy episodic alcohol consumption, smoking status, vegetable intake, and fruit intake.

**Fig M** Analysis of the effect of age at which ceased to meet physical activity guidelines on SF-36 component scores – sensitivity analysis excluding covariates in waves where they were completely missing.

**
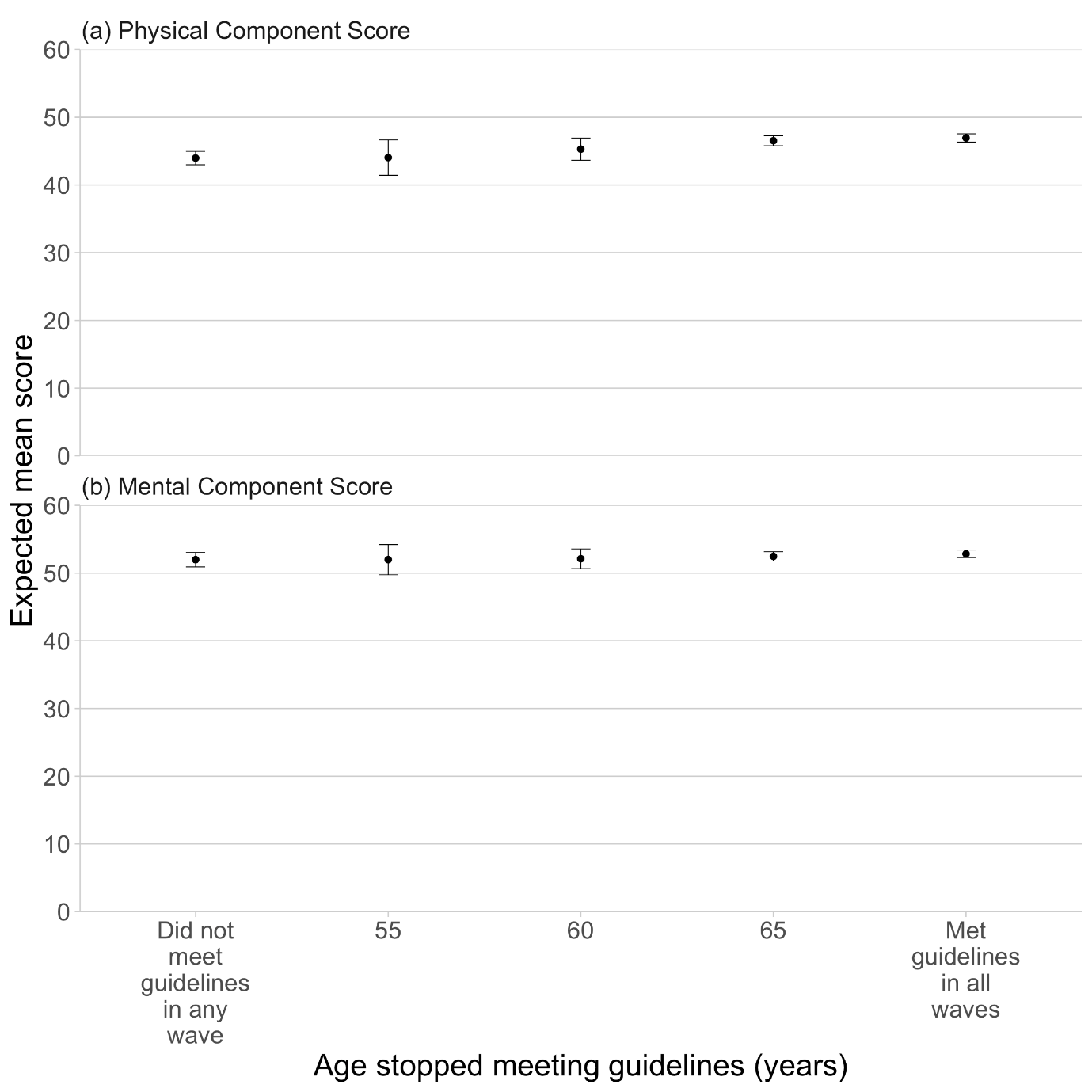
**

This figure shows the effect of meeting physical activity guidelines up to a particular age (55, 60, or 65 years), and then ceasing to meet guidelines thereafter (‘non-sustainers’), on the physical health component and mental health component scores at survey 9. The points represent the estimates and the bars the 99.5% confidence intervals.

Models were adjusted for: highest level of education, country of birth, age, employment status, living with children, marital status, Socio-Economic Index For Areas Index of Relative Socio-Economic Disadvantage (SEIFA IRSD), geographical remoteness (Accessibility-Remoteness Index of Australia Plus, ARIA+), history of coronary heart disease diagnosis/treatment, history of stroke diagnosis/treatment, history of arthritis diagnosis/treatment, history of any cancer diagnosis/treatment, history of anxiety diagnosis/treatment, and history of depression diagnosis/treatment, Center for Epidemiological Studies-Depression (CES-D) scale, stress, SF-36 subscale scores, body mass index, lifetime risky alcohol consumption based on the 2020 National Health Medical Research Council guidelines, heavy episodic alcohol consumption, smoking status, vegetable intake, and fruit intake.

**
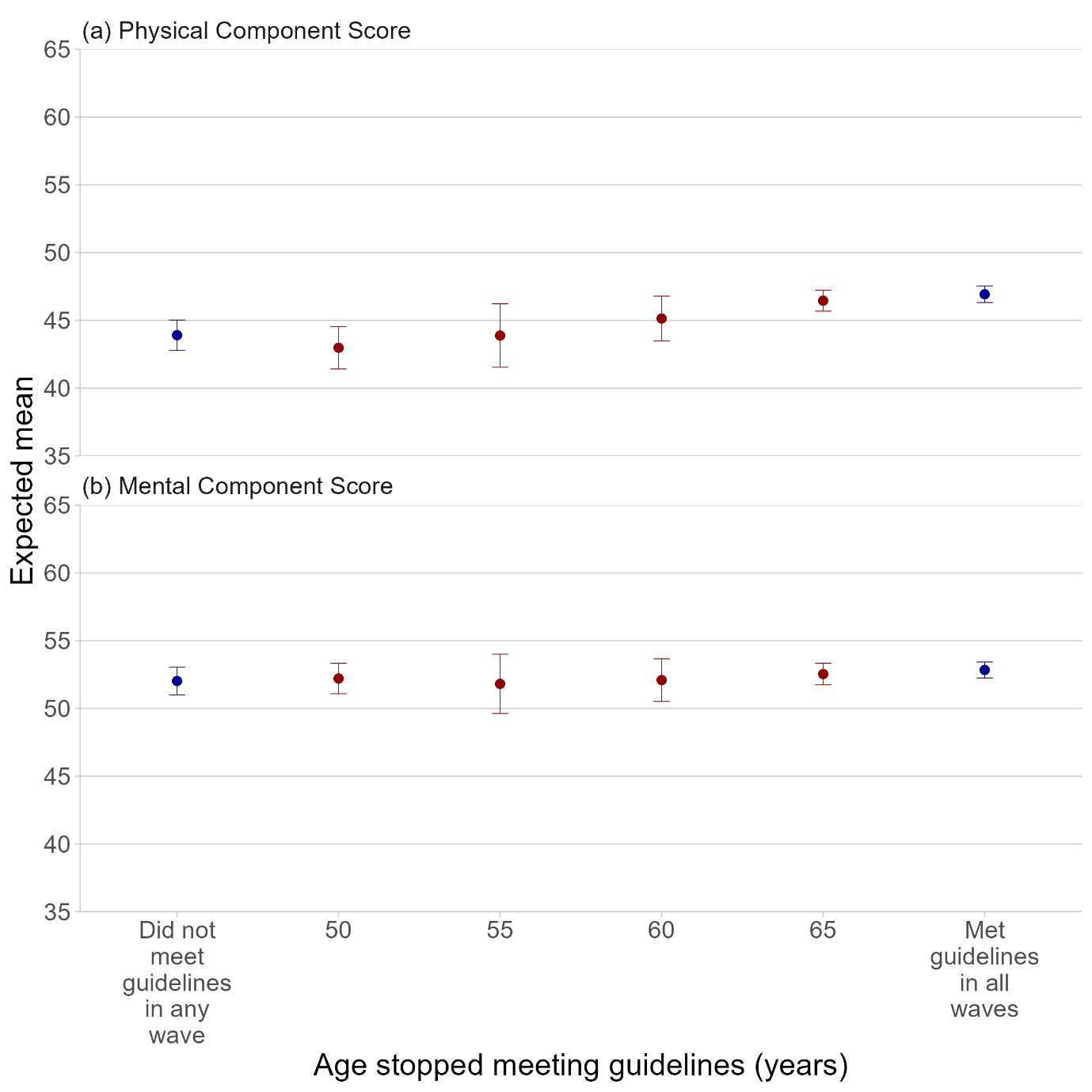
**

**Table H** Analysis of the effect of age at which started/ceased to meet physical activity guidelines SF-36 subscales – sensitivity analysis excluding covariates in waves where they were completely missing.

| **Counterfactual** | | **Expected Mean (99.5% CI)** | | | | | | | |
| --- | --- | --- | --- | --- | --- | --- | --- | --- | --- |
|  |  | **General Health** | **Physical functioning** | **Role physical** | **Bodily pain** | **Vitality** | **Social functioning** | **Role emotional** | **Mental health** |
| Age started meeting guidelines (years) | Met guidelines in all waves | 73.39^a^  (72.27, 74.51) | 80.60^a^  (79.31, 81.89) | 76.05^a^  (73.55, 78.55) | 69.01^a^  (67.60, 70.41) | 66.21^a^  (65.00, 67.42) | 86.73^a^  (85.31, 88.15) | 87.31  (85.18, 89.43) | 79.93^a^  (78.99, 80.86) |
|  | 55 | 72.50^b^  (69.40, 75.60) | 79.90^b^  (77.12, 82.67) | 77.60^b^  (71.15, 84.06) | 69.23^b^  (65.47, 72.98) | 65.64^b^  (63.28, 68.00) | 87.68^b^  (84.33, 91.04) | 89.43^b^  (84.96, 93.89) | 79.37  (76.94, 81.79) |
|  | 60 | 70.67  (68.07, 73.26) | 73.96  (70.21, 77.70) | 74.20  (67.77, 80.63) | 66.59  (63.33, 69.85) | 63.49  (60.83, 66.15) | 84.27  (80.91, 87.63) | 84.34  (79.12, 89.56) | 78.92  (76.61, 81.23) |
|  | 65 | 69.09  (66.82, 71.36) | 72.97  (70.29, 75.66) | 70.09  (64.93, 75.26) | 64.39  (61.57, 67.20) | 61.54  (59.11, 63.97) | 84.10  (81.51, 86.69) | 85.94  (82.44, 89.44) | 78.16  (76.21, 80.11) |
|  | Did not meet guidelines in any wave | 68.42  (66.28, 70.56) | 71.24  (68.95, 73.53) | 70.57  (65.85, 75.28) | 64.26  (61.66, 66.87) | 60.97  (58.84, 63.10) | 82.87  (80.37, 85.38) | 84.01  (80.41, 87.60) | 78.00  (76.36, 79.63) |
| Age stopped meeting guidelines (years) | Did not meet guidelines in any wave | 68.42  (66.28, 70.56) | 71.24  (68.95, 73.53) | 70.57  (65.85, 75.28) | 64.26  (61.66, 66.87) | 60.97  (58.84, 63.10) | 82.87  (80.37, 85.38) | 84.01  (80.41, 87.60) | 78.00  (76.36, 79.63) |
|  | 55 | 68.96  (65.69, 72.24) | 73.84  (68.27, 79.42) | 67.07  (58.45, 75.70) | 63.66  (59.04, 68.28) | 60.92  (56.77, 65.07) | 82.75  (78.08, 87.43) | 83.43  (76.15, 90.71) | 78.13  (75.07, 81.18) |
|  | 60 | 70.31  (67.68, 72.93) | 76.06  (72.59, 79.53) | 71.01  (65.87, 76.16) | 65.71  (62.70, 68.73) | 63.36  (60.70, 66.02) | 84.02  (80.78, 87.27) | 83.67  (79.18, 88.16) | 78.74  (76.03, 81.45) |
|  | 65 | 72.63^d^  (71.00, 74.26) | 79.79  (78.22, 81.37) | 74.08  (71.04, 77.13) | 67.95^d^  (66.01, 69.89) | 65.32^d^  (63.77, 66.86) | 86.15^d^  (84.48, 87.82) | 85.99  (83.34, 88.63) | 79.41  (78.18, 80.64) |
|  | Met guidelines in all waves | 73.39^a^  (72.27, 74.51) | 80.60^a^  (79.31, 81.89) | 76.05^a^  (73.55, 78.55) | 69.01^a^  (67.60, 70.41) | 66.21^a^  (65.00, 67.42) | 86.73^a^  (85.31, 88.15) | 87.31  (85.18, 89.43) | 79.93^a^  (78.99, 80.86) |

Abbreviation: CI, confidence interval; SF-36, 36-item Medical Outcomes Study short-form survey..

^a^ Significantly different from reference group (did not meet guidelines in any wave).

^b^ Significantly different from reference group (did not meet guidelines in any wave).

^c^ Significantly different from reference group (did not meet guidelines in any wave).

^d^ Significantly different from reference group (did not meet guidelines in any wave).

**Fig N** Analysis of the effect of age at which started to meet physical activity guidelines on SF-36 subscales – sensitivity analysis excluding covariates in waves where they were completely missing.


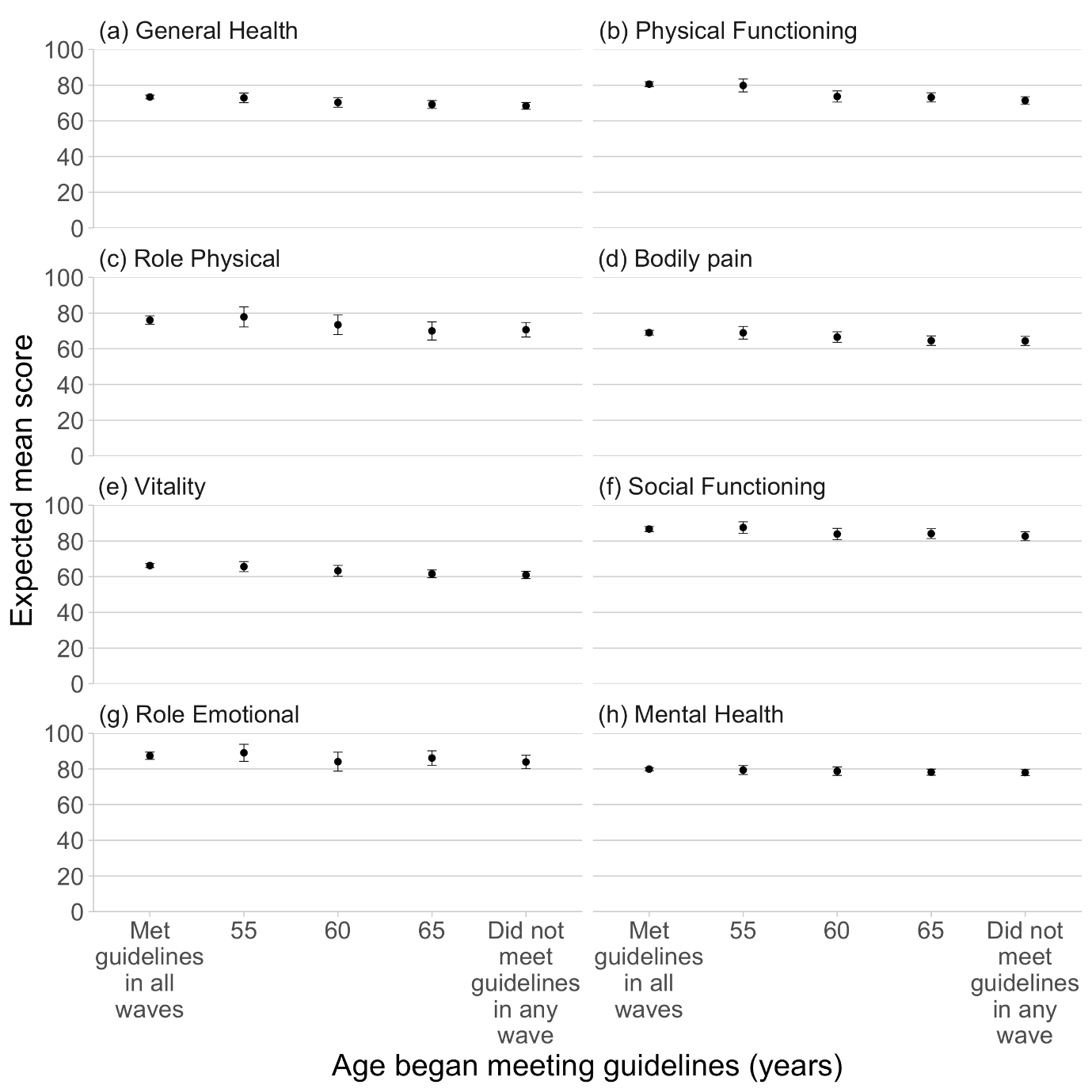

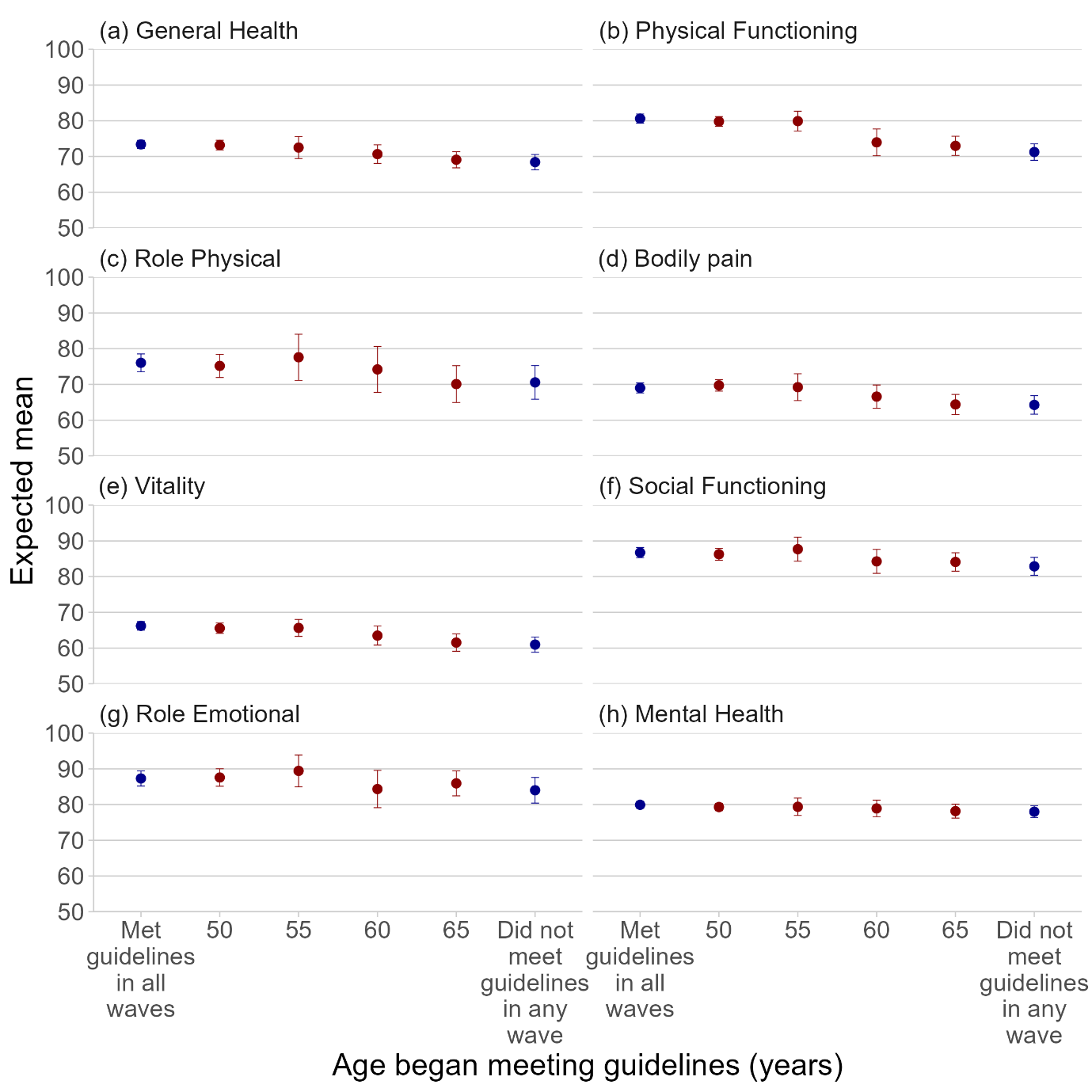


This figure shows the effect of meeting physical activity upon reaching a particular age (‘starters’) but not prior to that, with a range of age thresholds considered (55, 60, and 65 years), on SF-36 subscale scores at survey 9. The points represent the estimates and the bars the 99.5% confidence intervals.

Models were adjusted for: highest level of education, country of birth, age, employment status, living with children, marital status, Socio-Economic Index For Areas Index of Relative Socio-Economic Disadvantage (SEIFA IRSD), geographical remoteness (Accessibility-Remoteness Index of Australia Plus, ARIA+), history of coronary heart disease diagnosis/treatment, history of stroke diagnosis/treatment, history of arthritis diagnosis/treatment, history of any cancer diagnosis/treatment, history of anxiety diagnosis/treatment, and history of depression diagnosis/treatment, Center for Epidemiological Studies-Depression (CES-D) scale, stress, SF-36 subscale scores, body mass index, lifetime risky alcohol consumption based on the 2020 National Health Medical Research Council guidelines, heavy episodic alcohol consumption, smoking status, vegetable intake, and fruit intake.

**Fig O** Analysis of the effect of age at which ceased to meet physical activity guidelines on SF-36 subscales – sensitivity analysis excluding covariates in waves where they were completely missing.


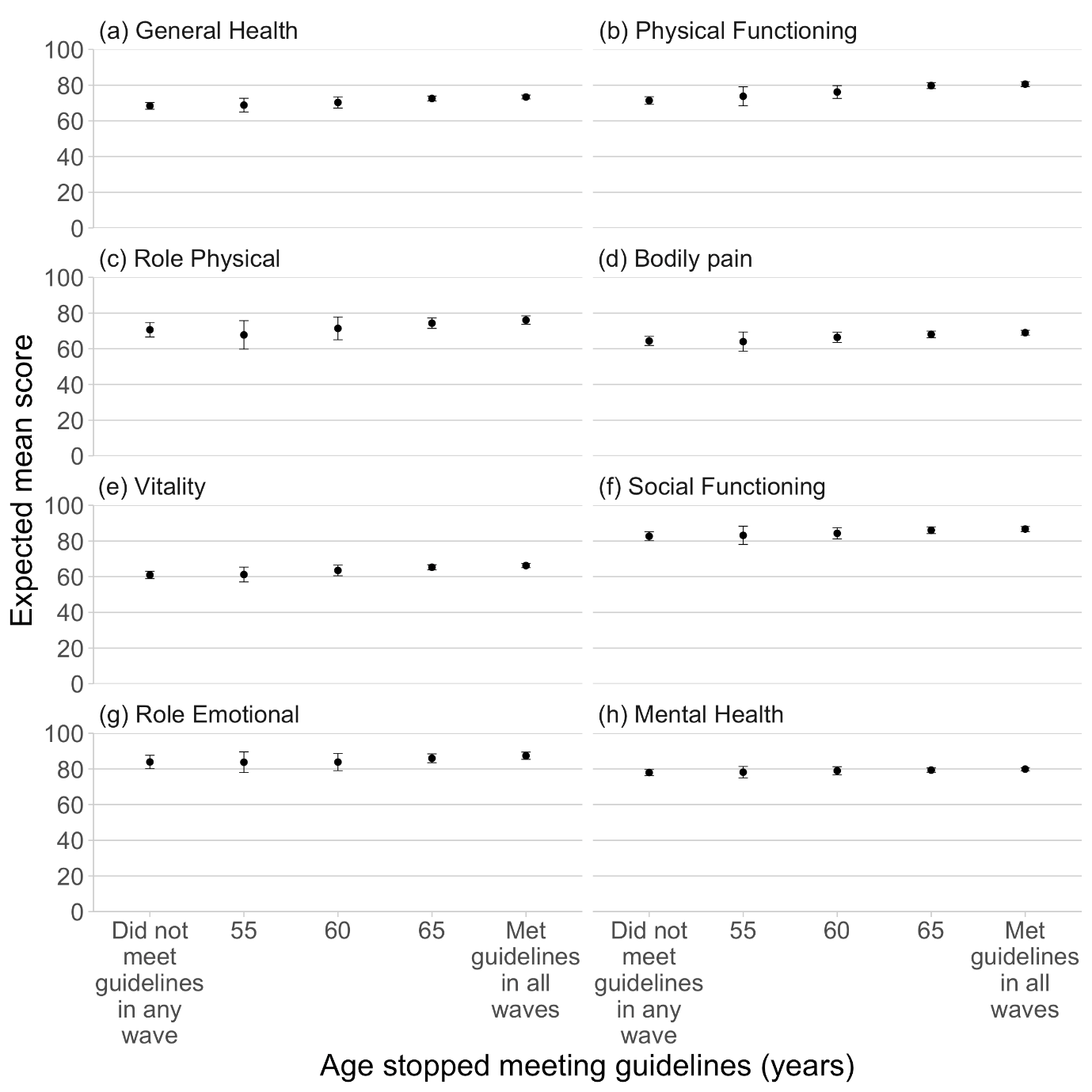

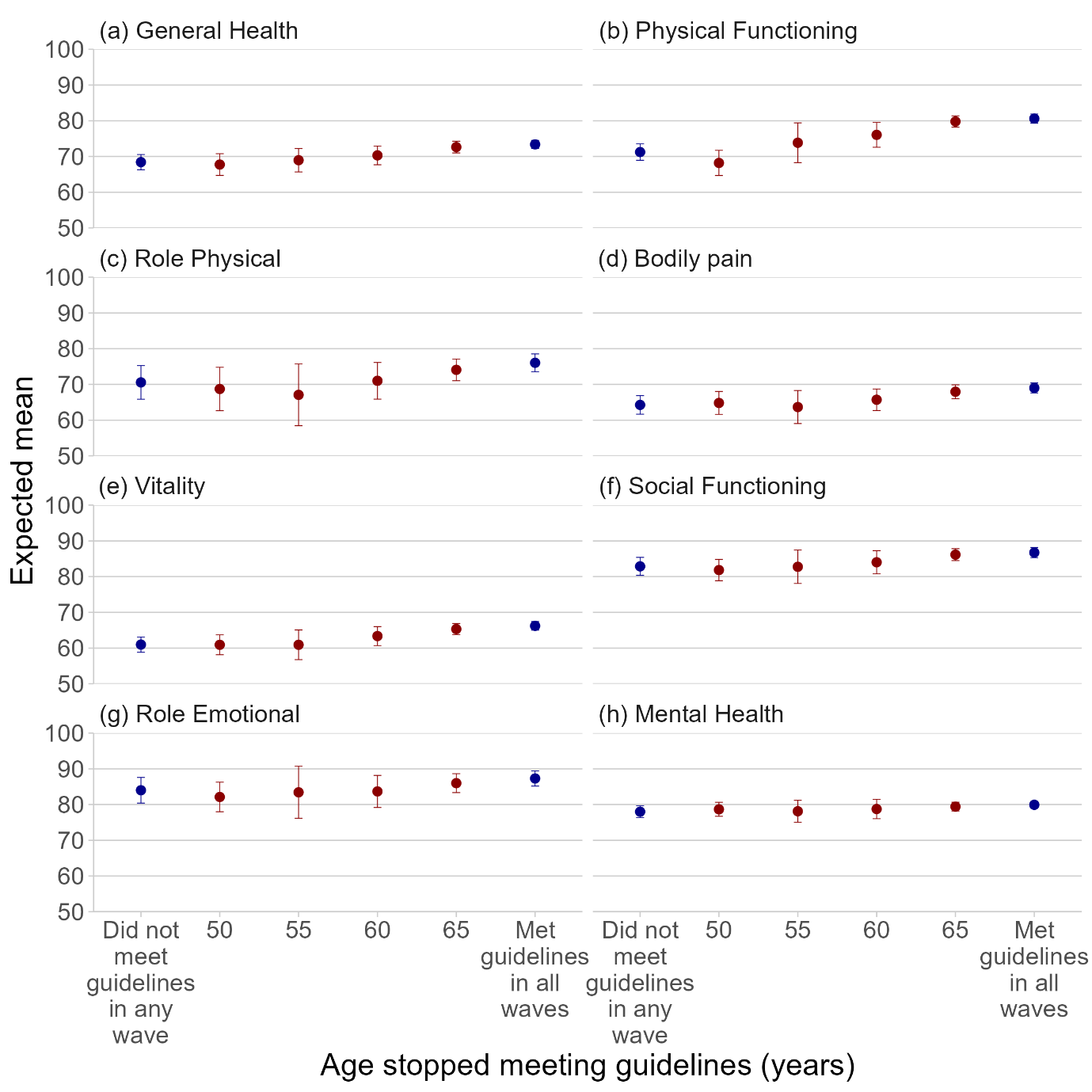


This figure shows the effect of meeting physical activity guidelines up to a particular age (55, 60, or 65 years), and then ceasing to meet guidelines thereafter (‘non-sustainers’), on SF-36 subscales at survey 9. The points represent the estimates and the bars the 99.5% confidence intervals.

Models were adjusted for: highest level of education, country of birth, age, employment status, living with children, marital status, Socio-Economic Index For Areas Index of Relative Socio-Economic Disadvantage (SEIFA IRSD), geographical remoteness (Accessibility-Remoteness Index of Australia Plus, ARIA+), history of coronary heart disease diagnosis/treatment, history of stroke diagnosis/treatment, history of arthritis diagnosis/treatment, history of any cancer diagnosis/treatment, history of anxiety diagnosis/treatment, and history of depression diagnosis/treatment, Center for Epidemiological Studies-Depression (CES-D) scale, stress, SF-36 subscale scores, body mass index, lifetime risky alcohol consumption based on the 2020 National Health Medical Research Council guidelines, heavy episodic alcohol consumption, smoking status, vegetable intake, and fruit intake.

**Table I** Mean differences, 99.5% confidence intervals and E-Value analysis of the physical component and mental component scales.

| **Counterfactual** | | **Physical Component Score** | | |  | **Mental Component Score** | |  |  |
| --- | --- | --- | --- | --- | --- | --- | --- | --- | --- |
|  |  | **Mean difference (99.5% CI)** | **p-value** | **E-Value** | | **Mean difference (99.5% CI)** | **p-value** | **E-Value** | |
|  |  |  |  | **At estimate** | **At CI bound** |  |  | **At estimate** | **At CI bound** |
| Age started meeting guidelines (years) | Met guidelines in all waves | 3.0 (1.8, 4.1) | p<0.001 | 1.9 | 1.6 | 0.9 (-0.3, 2.0) | p=0.036 | 1.4 | 1.0 |
|  | 55 | 3.0 (1.2, 4.8) | p<0.001 | 1.9 | 1.5 | 0.9 (-0.8, 2.6) | p=0.143 | 1.4 | 1.0 |
|  | 60 | 1.1 (-0.6, 2.8) | p=0.076 | 1.4 | 1.0 | 0.2 (-1.7, 2.1) | p=0.783 | 1.2 | 1.0 |
|  | 65 | 0.2 (-0.9, 1.3) | p=0.589 | 1.2 | 1.0 | 0.4 (-0.6, 1.4) | p=0.269 | 1.2 | 1.0 |
|  | Did not meet guidelines in any wave | REF |  |  |  | REF |  |  | 0.0 |
| Age stopped meeting guidelines (years) | Did not meet guidelines in any wave | REF |  |  |  | REF |  |  |  |
|  | 55 | 0.1 (-2.9, 3.0) | p=0.959 | 1.1 | 1.0 | 0.0 (-2.6, 2.5) | p=0.973 | 1.1 | 1.0 |
|  | 60 | 1.3 (-0.5, 3.2) | p=0.046 | 1.5 | 1.0 | 0.1 (-1.6, 1.9) | p=0.812 | 1.1 | 1.0 |
|  | 65 | 2.6 (1.4, 3.8) | p<0.001 | 1.8 | 1.5 | 0.5 (-0.7, 1.7) | p=0.239 | 1.3 | 1.0 |
|  | Met guidelines in all waves | 3.0 (1.8, 4.1) | p<0.001 | 1.9 | 1.6 | 0.9 (-0.3, 2.0) | p=0.036 | 1.4 | 1.0 |

Abbreviation: CI, confidence interval.

Note: Evalue is 1.0 when CI crosses null as no additional confounding is needed to result in inconclusive findings.

**Table J** Mean differences, 99.5% confidence interval and E-Value analysis of the SF-36 subscales.

| **Counterfactual** | | **General Health** | | | | **Physical functioning** | | | | **Role physical** | | | | **Bodily pain** | | | |
| --- | --- | --- | --- | --- | --- | --- | --- | --- | --- | --- | --- | --- | --- | --- | --- | --- | --- |
|  |  | **Mean difference (99.5% CI)** | **p-value** | **E-Value** | | **Mean difference (99.5% CI)** | **p-value** | **E-Value** | | **Mean difference (99.5% CI)** | **p-value** | **E-Value** | | **Mean difference (99.5% CI)** | **p-value** | **E-Value** | |
|  |  |  |  | **At estimate** | **At CI bound** |  |  | **At estimate** | **At CI bound** |  |  | **At estimate** | **At CI bound** |  |  | **At estimate** | **At CI bound** |
| Age started meeting guidelines (years) | Met guide-lines in all waves | 5.0 (2.6, 7.4) | p<0.001 | 1.8 | 1.5 | 9.3 (6.2, 12.3) | p<0.001 | 2.3 | 1.9 | 5.5 (-0.1, 11.0) | p=0.005 | 1.5 | 1.0 | 4.6 (1.5, 7.8) | p<0.001 | 1.7 | 1.3 |
|  | 55 | 4.5 (1.2, 7.8) | p<0.001 | 1.8 | 1.3 | 8.5 (4.0, 12.9) | p<0.001 | 2.2 | 1.7 | 7.2 (-0.7, 15.1) | p=0.011 | 1.6 | 1.0 | 4.6 (0.1, 9.0) | p=0.004 | 1.7 | 1.1 |
|  | 60 | 1.9 (-1.4, 5.2) | p=0.101 | 1.4 | 1.0 | 2.3 (-1.5, 6.1) | p=0.089 | 1.4 | 1.0 | 2.9 (-4.7, 10.5) | p=0.284 | 1.3 | 1.0 | 2.1 (-2.1, 6.3) | p=0.155 | 1.4 | 1.0 |
|  | 65 | 0.8 (-1.6, 3.1) | p=0.359 | 1.2 | 1.0 | 1.8 (-0.7, 4.2) | p=0.041 | 1.4 | 1.0 | -0.6 (-5.7, 4.5) | p=0.736 | 1.1 | 1.0 | 0.2 (-2.5, 2.8) | p=0.868 | 1.1 | 1.0 |
|  | Did not meet guide-lines in any wave | REF |  |  |  | REF |  |  |  | REF |  |  |  | REF |  |  |  |
| Age stopped meeting guidelines (years) | Did not meet guide-lines in any wave | REF |  |  |  | REF |  |  |  | REF |  |  |  | REF |  |  |  |
|  | 55 | 0.4 (-3.9, 4.7) | p=0.787 | 1.2 | 1.0 | 2.4 (-3.5, 8.4) | p=0.254 | 1.5 | 1.0 | -2.9 (-12.8, 7.0) | p=0.409 | 1.3 | 1.0 | -0.4 (-6.6, 5.8) | p=0.864 | 1.1 | 1.0 |
|  | 60 | 1.9 (-1.9, 5.7) | p=0.157 | 1.4 | 1.0 | 4.8 (0.5, 9.2) | p=0.002 | 1.8 | 1.2 | 0.8 (-7.1, 8.8) | p=0.769 | 1.2 | 1.0 | 2.0 (-2.0, 6.0) | p=0.167 | 1.4 | 1.0 |
|  | 65 | 4.2 (1.6, 6.7) | p<0.001 | 1.7 | 1.4 | 8.4 (5.1, 11.8) | p<0.001 | 2.2 | 1.8 | 3.7 (-1.8, 9.3) | p=0.058 | 1.4 | 1.0 | 3.7 (0.4, 6.9) | p=0.002 | 1.6 | 1.1 |
|  | Met guide-lines in all waves | 5.0 (2.6, 7.4) | p<0.001 | 1.8 | 1.5 | 9.3 (6.2, 12.3) | p<0.001 | 2.3 | 1.9 | 5.5 (-0.1, 11.0) | p=0.005 | 1.5 | 1.0 | 4.6 (1.5, 7.8) | p<0.001 | 1.7 | 1.3 |
|  | | | | | | | | |  |  |  |  |  |  |  |  |  |
|  |  |  |  |  |  |  |  |  |  |  |  |  |  |  |  |  |  |

| **Counterfactual** | | **Vitality** | | | | **Social functioning** | | | | **Role emotional** | | | | | **Mental health** | | | |
| --- | --- | --- | --- | --- | --- | --- | --- | --- | --- | --- | --- | --- | --- | --- | --- | --- | --- | --- |
|  | | **Mean difference (99.5% CI)** | **p-value** | **E-Value** | | **Mean difference (99.5% CI)** | **p-value** | **E-Value** | | **Mean difference (99.5% CI)** | **p-value** | **E-Value** | | **Mean difference (99.5% CI)** | | **p-value** | **E-Value** | |
|  |  |  |  | **At estimate** | **At CI bound** |  |  | **At estimate** | **At CI bound** |  |  | **At estimate** | **At CI bound** |  |  |  | **At estimate** | **At CI bound** |
| Age started meeting guidelines (years) | Met guide-lines in all waves | 5.3 (2.9, 7.6) | p<0.001 | 1.9 | 1.5 | 4.1 (-0.3, 8.4) | p=0.009 | 1.6 | 1.0 | 3.5 (-3.0, 10.1) | p=0.129 | 1.5 | 1.0 | 2.0 (-0.5, 4.4) | | p=0.025 | 1.5 | 1.0 |
|  | 55 | 4.7 (1.3, 8.0) | p<0.001 | 1.8 | 1.3 | 4.9 (-0.3, 10.1) | p=0.008 | 1.7 | 1.0 | 5.1 (-3.5, 13.7) | p=0.095 | 1.6 | 1.0 | 1.5 (-1.8, 4.7) | | p=0.210 | 1.4 | 1.0 |
|  | 60 | 2.3 (-1.3, 6.0) | p=0.068 | 1.5 | 1.0 | 1.3 (-3.7, 6.3) | p=0.480 | 1.3 | 1.0 | 0.2 (-7.7, 8.1) | p=0.938 | 1.1 | 1.0 | 0.8 (-2.6, 4.1) | | p=0.510 | 1.3 | 1.0 |
|  | 65 | 0.6 (-1.3, 2.5) | p=0.352 | 1.2 | 1.0 | 1.4 (-1.8, 4.7) | p=0.219 | 1.3 | 1.0 | 2.2 (-2.8, 7.2) | p=0.213 | 1.3 | 1.0 | 0.2 (-1.6, 2.1) | | p=0.724 | 1.1 | 1.0 |
|  | Did not meet guide-lines in any wave | REF |  |  |  | REF |  |  |  | REF |  |  |  | REF | |  |  |  |
| Age stopped meeting guidelines (years) | Did not meet guide-lines in any wave | REF |  |  |  | REF |  |  |  | REF |  |  |  | REF | |  |  |  |
|  | 55 | 0.2 (-4.2, 4.6) | p=0.890 | 1.1 | 1.0 | 0.5 (-6.3, 7.3) | p=0.835 | 1.2 | 1.0 | -0.2 (-8.4, 8.1) | p=0.959 | 1.1 | 1.0 | 0.3 (-4.0, 4.5) | | p=0.868 | 1.1 | 1.0 |
|  | 60 | 2.6 (-0.9, 6.0) | p=0.039 | 1.5 | 1.0 | 1.7 (-3.6, 6.9) | p=0.371 | 1.3 | 1.0 | 0.0 (-7.6, 7.6) | p=0.995 | 1.0 | 1.0 | 1.0 (-2.2, 4.3) | | p=0.367 | 1.3 | 1.0 |
|  | 65 | 4.3 (1.9, 6.7) | p<0.001 | 1.7 | 1.4 | 3.4 (-1.0, 7.9) | p=0.030 | 1.6 | 1.0 | 2.1 (-4.7, 8.9) | p=0.392 | 1.3 | 1.0 | 1.4 (-1.0, 3.9) | | p=0.107 | 1.4 | 1.0 |
|  | Met guide-lines in all waves | 5.3 (2.9, 7.6) | p<0.001 | 1.9 | 1.5 | 4.1 (-0.3, 8.4) | p=0.009 | 1.6 | 1.0 | 3.5 (-3.0, 10.1) | p=0.129 | 1.5 | 1.0 | 2.0 (-0.5, 4.4) | | p=0.025 | 1.5 | 1.0 |

Abbreviations: CI, confidence interval; SF-36, 36-item Medical Outcomes Study short-form survey.

Note: E-value is 1.0 when CI crosses null as no additional confounding is needed to result in inconclusive findings.

**Fig P** Analysis of the effect of age at which started to meet physical activity guidelines on SF-36 component scores – sensitivity analysis excluding mental health confounders.


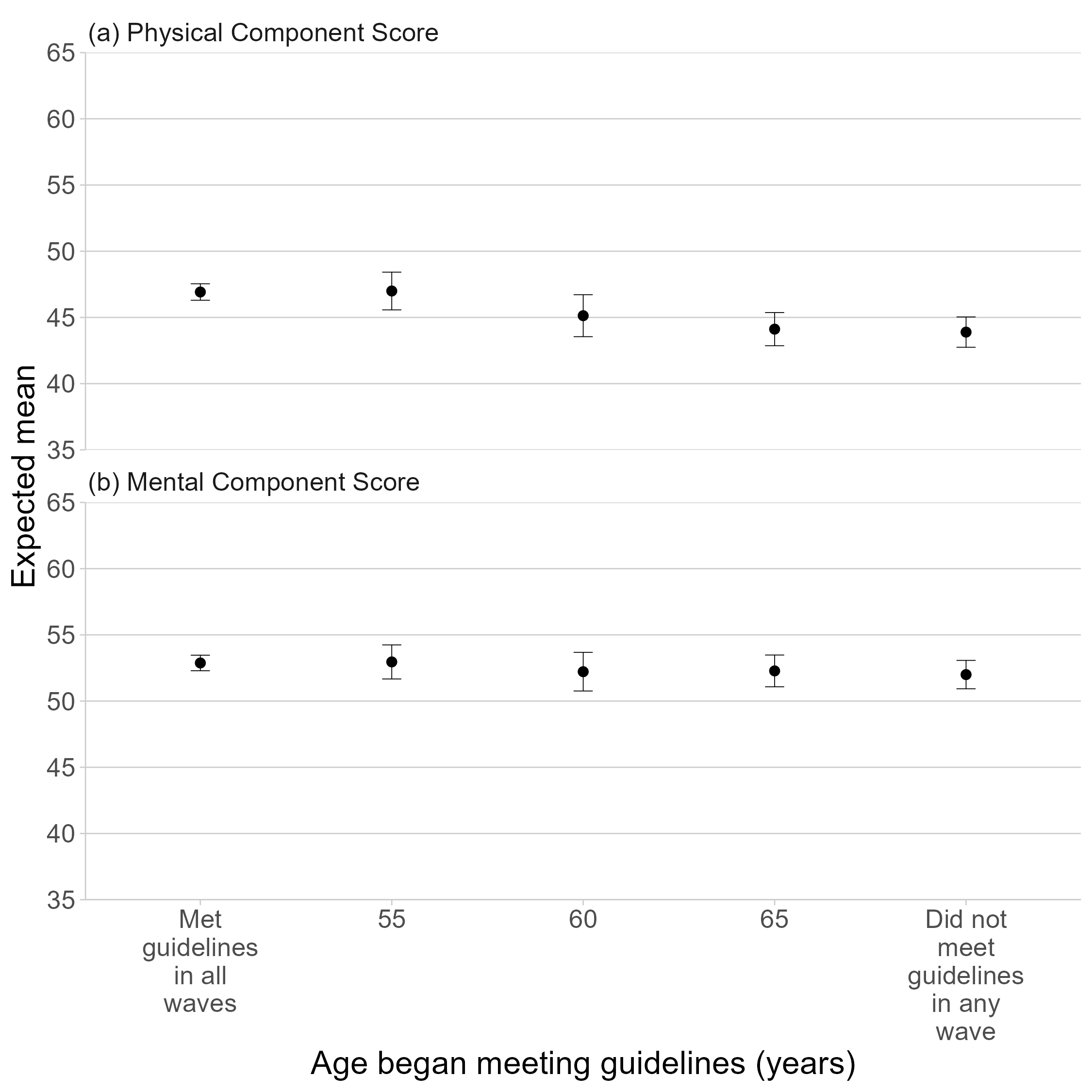


This figure shows the effect of meeting physical activity upon reaching a particular age (‘starters’) but not prior to that, with a range of age thresholds considered (55, 60, and 65 years), on the physical health component and mental health component scores at survey 9. The points represent the estimates and the bars the 99.5% confidence intervals.

Models were adjusted for: highest level of education, country of birth, age, employment status, living with children, marital status, Socio-Economic Index For Areas Index of Relative Socio-Economic Disadvantage (SEIFA IRSD), geographical remoteness (Accessibility-Remoteness Index of Australia Plus, ARIA+), history of coronary heart disease diagnosis/treatment, history of stroke diagnosis/treatment, history of arthritis diagnosis/treatment, history of any cancer diagnosis/treatment, history of anxiety diagnosis/treatment, and history of depression diagnosis/treatment, Center for Epidemiological Studies-Depression (CES-D) scale, stress, SF-36 subscale scores, body mass index, lifetime risky alcohol consumption based on the 2020 National Health Medical Research Council guidelines, heavy episodic alcohol consumption, smoking status, vegetable intake, and fruit intake.

**Fig Q** Analysis of the effect of age at which started to meet physical activity guidelines on SF-36 subscales – sensitivity analysis excluding mental health confounders.


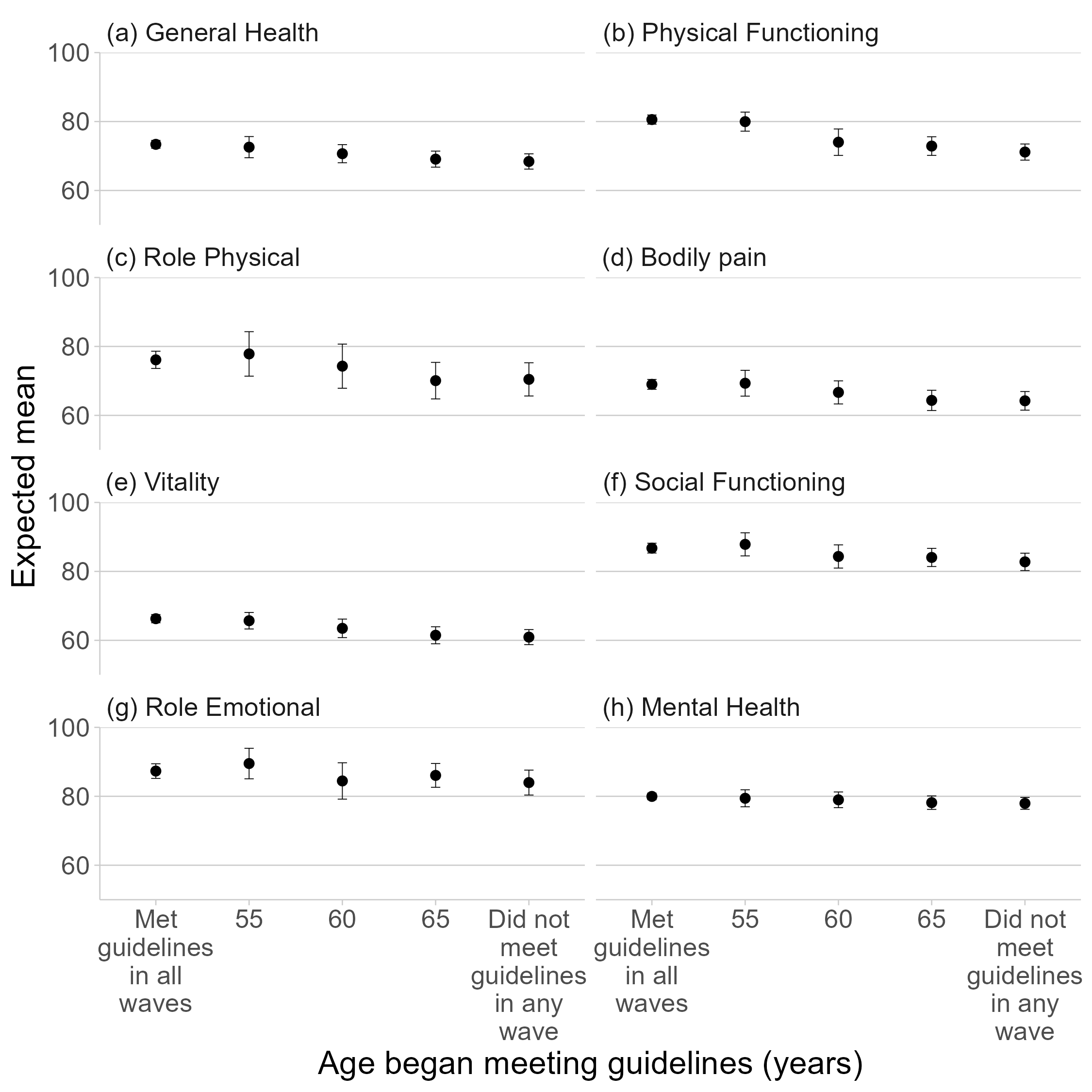


This figure shows the effect of meeting physical activity upon reaching a particular age (‘starters’) but not prior to that, with a range of age thresholds considered (55, 60, and 65 years), on SF-36 subscale scores at survey 9. The points represent the estimates and the bars the 99.5% confidence intervals.

Models were adjusted for: highest level of education, country of birth, age, employment status, living with children, marital status, Socio-Economic Index For Areas Index of Relative Socio-Economic Disadvantage (SEIFA IRSD), geographical remoteness (Accessibility-Remoteness Index of Australia Plus, ARIA+), history of coronary heart disease diagnosis/treatment, history of stroke diagnosis/treatment, history of arthritis diagnosis/treatment, history of any cancer diagnosis/treatment, history of anxiety diagnosis/treatment, and history of depression diagnosis/treatment, Center for Epidemiological Studies-Depression (CES-D) scale, stress, SF-36 subscale scores, body mass index, lifetime risky alcohol consumption based on the 2020 National Health Medical Research Council guidelines, heavy episodic alcohol consumption, smoking status, vegetable intake, and fruit intake.

**Fig R** Analysis of the effect of age at which ceased to meet physical activity guidelines on SF-36 component scores – sensitivity analysis excluding mental health confounders.


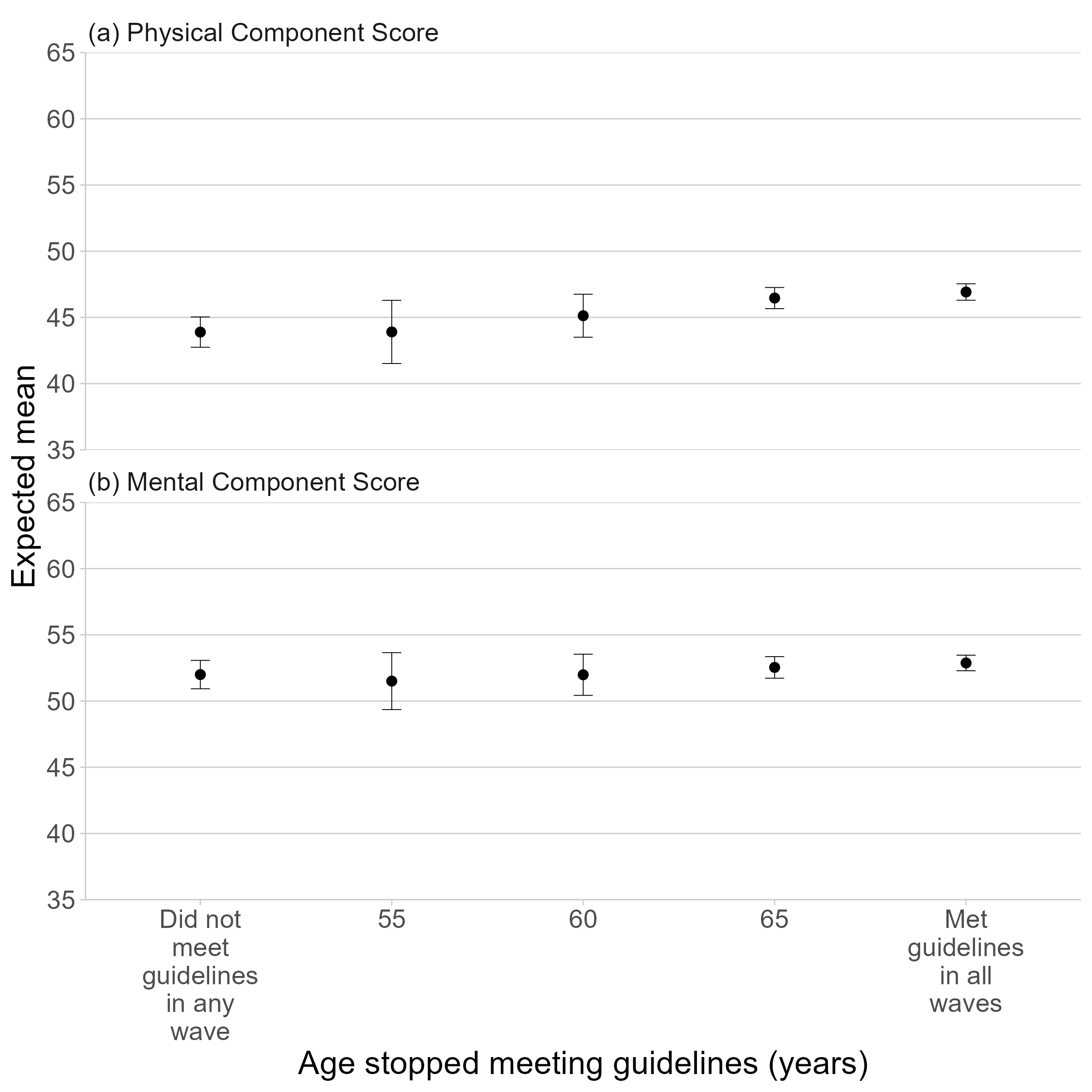


This figure shows the effect of meeting physical activity guidelines up to a particular age (55, 60, or 65 years), and then ceasing to meet guidelines thereafter (‘non-sustainers’), on the physical health component and mental health component scores at survey 9. The points represent the estimates and the bars the 99.5% confidence intervals.

Models were adjusted for: highest level of education, country of birth, age, employment status, living with children, marital status, Socio-Economic Index For Areas Index of Relative Socio-Economic Disadvantage (SEIFA IRSD), geographical remoteness (Accessibility-Remoteness Index of Australia Plus, ARIA+), history of coronary heart disease diagnosis/treatment, history of stroke diagnosis/treatment, history of arthritis diagnosis/treatment, history of any cancer diagnosis/treatment, history of anxiety diagnosis/treatment, and history of depression diagnosis/treatment, Center for Epidemiological Studies-Depression (CES-D) scale, stress, SF-36 subscale scores, body mass index, lifetime risky alcohol consumption based on the 2020 National Health Medical Research Council guidelines, heavy episodic alcohol consumption, smoking status, vegetable intake, and fruit intake.

**Fig S** Analysis of the effect of age at which ceased to meet physical activity guidelines on SF-36 subscales – sensitivity analysis excluding mental health confounders.


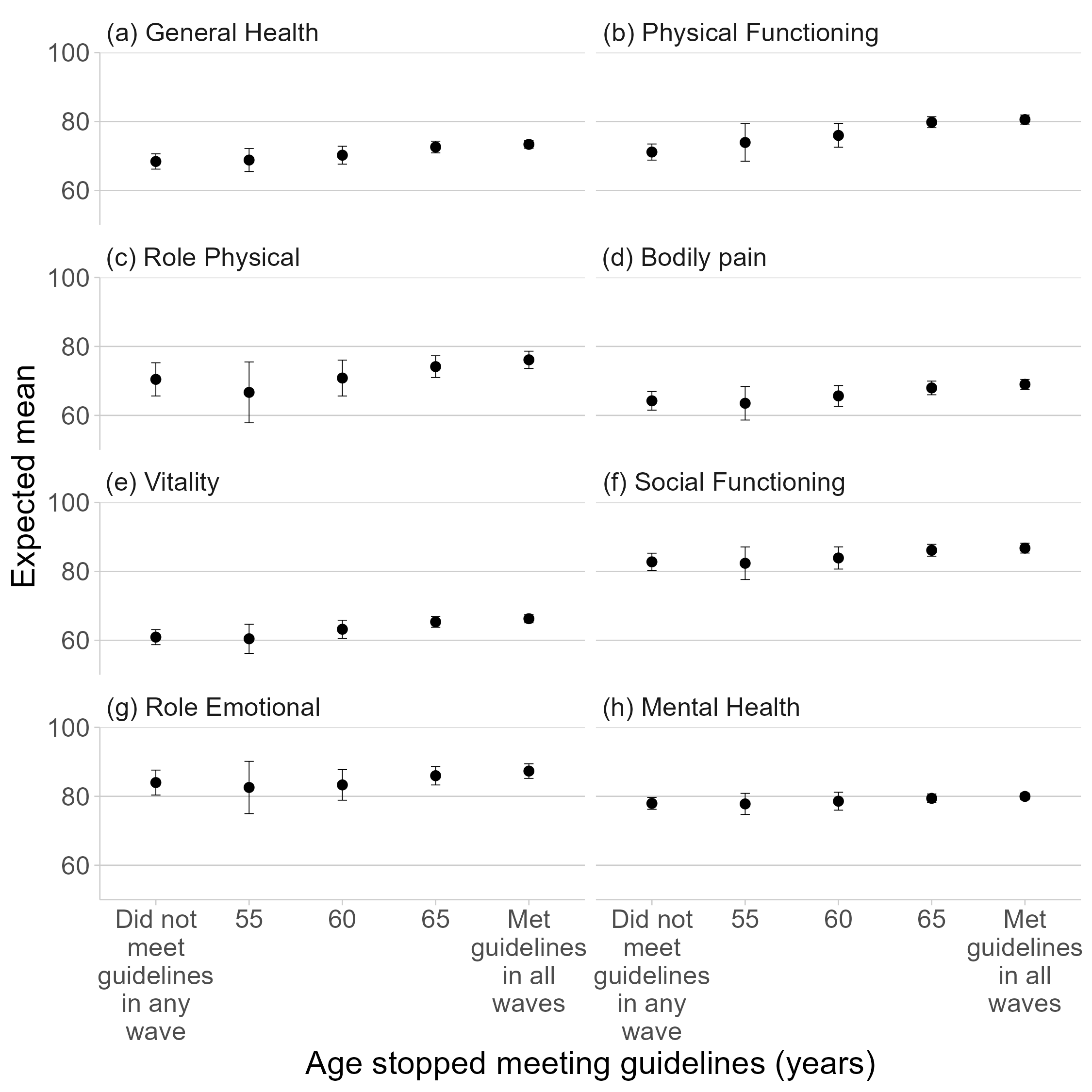


This figure shows the effect of meeting physical activity guidelines up to a particular age (55, 60, or 65 years), and then ceasing to meet guidelines thereafter (‘non-sustainers’), on SF-36 subscale scores at survey 9. The points represent the estimates and the bars the 99.5% confidence intervals.

Models were adjusted for: highest level of education, country of birth, age, employment status, living with children, marital status, Socio-Economic Index For Areas Index of Relative Socio-Economic Disadvantage (SEIFA IRSD), geographical remoteness (Accessibility-Remoteness Index of Australia Plus, ARIA+), history of coronary heart disease diagnosis/treatment, history of stroke diagnosis/treatment, history of arthritis diagnosis/treatment, history of any cancer diagnosis/treatment, history of anxiety diagnosis/treatment, and history of depression diagnosis/treatment, Center for Epidemiological Studies-Depression (CES-D) scale, stress, SF-36 subscale scores, body mass index, lifetime risky alcohol consumption based on the 2020 National Health Medical Research Council guidelines, heavy episodic alcohol consumption, smoking status, vegetable intake, and fruit intake.
