# Supplementary material for: Physical activity across midlife and health-related quality of life in Australian women: A target trial emulation using a longitudinal cohort
Source: PLoS Med. 2024 May 2;21(5):e1004384. doi: 10.1371/journal.pmed.1004384 (PMC11065283; doi:10.1371/journal.pmed.1004384)
Supplement: S7 Text — (DOCX) [file pmed.1004384.s008.docx]

**S7 Text**

**Post-hoc sensitivity analysis findings**

**Table A** Mean differences, 99.5% confidence interval and E-Value analysis of the physical component and mental component scales in post-hoc sensitivity analysis where all missing data were imputed.

| **Counterfactual** | | **Physical Component Score** | | |  | **Mental Component Score** | |  |  |
| --- | --- | --- | --- | --- | --- | --- | --- | --- | --- |
|  |  | **Mean difference (99.5% CI)** | **p-value** | **E-Value** | | **Mean difference (99.5% CI)** | **p-value** | **E-Value** | |
|  |  |  |  | **At estimate** | **At CI bound** |  |  | **At estimate** | **At CI bound** |
| Age stopped meeting guidelines (years) | Did not meet guidelines in any wave | REF |  |  |  | REF |  |  |  |
|  | 55 | 0.1 (-2.0, 2.1) | p=0.959 | 1.1 | 1.0 | 0.0 (-1.8, 1.7) | p=0.973 | 1.1 | 1.0 |
|  | 60 | 1.3 (0.0, 2.6) | p=0.046 | 1.5 | 1.0 | 0.1 (-1.1, 1.4) | p=0.812 | 1.1 | 1.0 |
|  | 65 | 2.6 (1.7, 3.4) | p<0.001 | 1.8 | 1.6 | 0.5 (-0.3, 1.3) | p=0.239 | 1.3 | 1.0 |
|  | Met guidelines in all waves | 3.0 (2.2, 3.8) | p<0.001 | 1.9 | 1.7 | 0.9 (0.1, 1.7) | p=0.036 | 1.4 | 1.1 |
| Age started meeting guidelines (years) | Met guidelines in all waves | 3.0 (2.2, 3.8) | p<0.001 | 1.9 | 1.7 | 0.9 (0.1, 1.7) | p=0.036 | 1.4 | 1.1 |
|  | 55 | 3.0 (1.7, 4.3) | p<0.001 | 1.9 | 1.6 | 0.9 (-0.3, 2.1) | p=0.143 | 1.4 | 1.0 |
|  | 60 | 1.1 (-0.1, 2.3) | p=0.076 | 1.4 | 1.0 | 0.2 (-1.1, 1.5) | p=0.783 | 1.2 | 1.0 |
|  | 65 | 0.2 (-0.6, 1.0) | p=0.589 | 1.2 | 1.0 | 0.4 (-0.3, 1.1) | p=0.269 | 1.2 | 1.0 |
|  | Did not meet guidelines in any wave | REF |  |  |  | REF |  |  |  |

Abbreviations: CI, confidence interval.

Note: Evalue is 1.0 when CI crosses null as no additional confounding is needed to result in inconclusive findings.
