# Supplementary material for: Physical activity across midlife and health-related quality of life in Australian women: A target trial emulation using a longitudinal cohort
Source: PLoS Med. 2024 May 2;21(5):e1004384. doi: 10.1371/journal.pmed.1004384 (PMC11065283; doi:10.1371/journal.pmed.1004384)
Supplement: S1 Fig — (DOCX) [file pmed.1004384.s009.docx]

**S1 Fig**

**Directed acyclic graph**

**A)** Directed acyclic graph showing the selection of observations

**B)** Directed acyclic graph showing the selection of variables


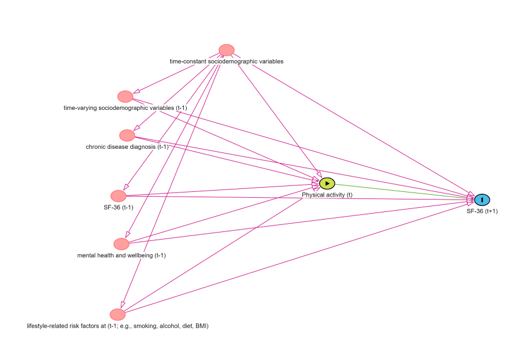


Note: Causal connections between the t-1 confounders were not drawn deliberately because they were measured contemporaneously, and thus do not meet ‘temporality’ requirements of cause-effect relationships.
